# Potential use of mealworm frass as soil amendment and pest management tool in circular economy

**DOI:** 10.1093/jee/toag057

**Published:** 2026-03-19

**Authors:** Luca Maria Girgenti, Marco Di Domenico, Rania Rachdi, Alessia Farina, Giuseppe Eros Massimino Cocuzza, Emanuele La Bella, Ferdinando Fragalà, Andrea Baglieri, Carmelo Rapisarda, Pompeo Suma

**Affiliations:** Department of Agriculture, Food and Environment, Applied Entomology Division, University of Catania, Catania, Italy; Department of Agriculture, Food and Environment, Applied Entomology Division, University of Catania, Catania, Italy; Department of Agriculture, Food and Environment, Applied Entomology Division, University of Catania, Catania, Italy; Department of Agriculture, Food and Environment, Applied Entomology Division, University of Catania, Catania, Italy; Department of Agriculture, Food and Environment, Applied Entomology Division, University of Catania, Catania, Italy; Department of Agriculture, Food and Environment, Agricultural Chemistry and Microbiology Division, University of Catania, Catania, Italy; Department of Agriculture, Food and Environment, Agricultural Chemistry and Microbiology Division, University of Catania, Catania, Italy; Department of Agriculture, Food and Environment, Agricultural Chemistry and Microbiology Division, University of Catania, Catania, Italy; Department of Agriculture, Food and Environment, Applied Entomology Division, University of Catania, Catania, Italy; Department of Agriculture, Food and Environment, Applied Entomology Division, University of Catania, Catania, Italy

**Keywords:** soil amendment, insect frass, plant growth, pest management

## Abstract

The global increase in human population is driving a growing demand for food and encouraging the exploration of alternative resources, including edible insects. The insect farming sector is expanding rapidly for both human consumption and animal feed, and one of its main byproducts is insect excreta, known as frass. This material has potential use as an organic soil amendment within a circular economy framework and may also influence pest population dynamics. This study investigated the effects of frass produced by *Tenebrio molitor* (Linnaeus) on plant growth and on the population density of the whitefly *Bemisia tabaci* (Gennadius). Frass was characterized for its physicochemical properties and applied at 3 doses to the growing substrate of tomato, sweet pepper and eggplant plants. Morphological and physiological traits such as plant height, number of leaves, leaf area, chlorophyll content, and plant biomass were evaluated. In addition, semi-field trials were conducted on artificially infested plants to assess general trends in whitefly population density in relation to the presence of frass. Overall, the use of frass as a soil amendment improved plant growth parameters across all tested species. The presence and dose of frass also influenced the general pattern of whitefly population density, with responses varying according to both host plant and amendment level.

## Riassunto

L’aumento globale della popolazione umana sta determinando una crescente domanda di cibo e incoraggiando la ricerca di risorse alternative, tra cui gli insetti commestibili. Il settore dell’allevamento di insetti, sia per il consumo umano che per l‘alimentazione animale, è in rapida espansione e genera come principale sottoprodotto da residui organici, noti come frass. Questo materiale ha un potenziale utilizzo come ammendante del suolo in un’ottica di economia circolare e può anche influenzare le dinamiche delle popolazioni di insetti fitofagi. Il presente studio ha valutato gli effetti del frass prodotto dal *Tenebrio molitor* (Linnaeus) sulla crescita delle piante e sulla densità della popolazione dell’aleirode *Bemisia tabaci* (Gennadius). Il frass è stato caratterizzato nelle sue proprietà fisico-chimiche e applicato, in tre dosi, al substrato di coltivazione di piante di pomodoro, peperone dolce e melanzana. Sono stati esaminati parametri morfologici e fisiologici quali altezza delle piante, numero di foglie, superficie fogliare, contenuto di clorofilla e biomassa. Inoltre, sono state condotte prove di semicampo su piante infestate artificialmente per valutare le tendenze generali della densità della popolazione dell’aleirode in funzione della presenza dell’ammendante. Nel complesso, l’impiego del frass come ammendante organico ha determinato un miglioramento dei principali parametri di crescita in tutte le specie vegetali testate. La presenza e la dose del materiale hanno inoltre influenzato la densità della popolazione dell’aleirode, con risposte variabili a seconda della pianta ospite e del livello di applicazione

## Introduction

The increasing demand for food, driven by the global population growth, poses significant challenges to crop production (Poveda 2021a) and creates the need for an estimated 70% increase in agricultural productivity (Molotoks et al. 2021, Van Dijk et al. 2021). This issue is further exacerbated by globalization and the impact of climate change, which contribute to the emergence and spread of new pests and pathogens (Fones et al. 2020, Hristov et al. 2020). In an effort to boost agricultural output, the widespread use of chemical inputs has become common in recent years, leading to serious negative consequences for the environment, biodiversity, and human health (Tripathi et al. 2020, Hossain et al. 2022).

In this context, a key challenge for agriculture is to identify alternatives to the widespread use of chemical inputs. Aligned with circular economy principles (Fuertes-Mendizábal et al. 2023), these strategies focus on utilizing sustainable soil amendments and pest control agents to promote environmental and agricultural sustainability (Hossain et al. 2022).

The development and use of new organic compounds that promote plant growth could significantly boost food production while safeguarding the environment (García-Fraile et al. 2015, Carvalho 2017, Menéndez et al. 2017, Shankar and Shikha 2017). In this context, the insect farming industry is rapidly gaining momentum worldwide (Blakstad et al. 2023), offering promising solutions for sustainable agricultural inputs (Zunzunegui et al. 2024) and efficient recycling of organic waste (Nyanzira et al. 2023). Insects produce excreta that are rich in nutrients readily absorbed by plants (Poveda et al. 2019). This material, combined with uneaten feed and insect exoskeleton fragments rich in chitin (Nyanzira et al. 2023), is collectively known as *frass*. As the most abundant by-product of insect farming, frass plays a key role in supporting circular economy practices (Nyanzira et al. 2023), with strong potential for agricultural applications (Blakstad et al. 2023, Fuertes-Mendizábal et al. 2023).

The yellow mealworm, *Tenebrio molitor* (Linnaeus) (Coleoptera: Tenebrionidae), was the first insect species approved by the European Union for use as human food (Toviho and Bársony 2022). Due to its life cycle and high nutritional value, *T. molitor* is considered one of the most promising species for large-scale insect farming (Arévalo et al. 2022). Recently, growing interest has emerged in using *T. molitor* frass as a valuable input in agriculture (Poveda 2021b, Barragán-Fonseca et al. 2022), particularly as an organic soil conditioner and plant fortifier. By supplying essential nutrients to the soil-along with chitin and beneficial microorganisms to crops (Poveda 2021b)—frass has the potential to enhance plant tolerance and resistance to both abiotic and biotic stresses (Blakstad et al. 2023, Zunzunegui et al. 2024). Depending on the applied dosage, frass can act as a biostimulant, promoting plant growth and development, or as an elicitor, triggering plant immune responses and supporting resilience under moderate stress conditions (Ferruzca-Campos et al. 2023).

Under natural conditions, the presence of frass enhances soil fertility thanks to its high concentrations of essential nutrients such as nitrogen (N), phosphorus (P), and potassium (K), as well as secondary elements like magnesium (Mg), copper (Cu), and zinc (Zn) (Fuertes-Mendizábal et al. 2023). This nutrient-rich profile makes frass a promising candidate to partially or fully replace mineral fertilizers.

Research has demonstrated its effectiveness in promoting the growth of various crops, including chard (Poveda et al. 2019), barley (Houben et al. 2020), ryegrass (Houben et al. 2021), and zucchini (Zim et al. 2022), while also reducing the need for chemical inputs (Houben et al. 2020). Additionally, frass has been shown to significantly increase the edible biomass of several vegetables-such as beetroot, arugula, lettuce, kale, nasturtium, cherry tomato, cucumber, and sweet corn-even under controlled greenhouse conditions (Fuertes-Mendizábal et al. 2023, Zunzunegui et al. 2024).

Plant growth promotion observed with frass application may also be linked to the presence of beneficial microorganisms that become active in the soil environment (Houben et al. 2020). These microbial communities have been shown to enhance plant tolerance to abiotic stresses such as salinity, drought, and flooding, as demonstrated in bean plants (Poveda et al. 2019).

Notably, frass contains various plant growth-promoting rhizobacteria as well as chitin-degrading bacteria like *Bacillus thuringiensis* (Berliner), *B. cereus* (Frankland and Frankland), and *Lysinibacillus sphaericus* (Meyer and Neide). These microorganisms are already used in commercial biocontrol products due to their antagonistic effects on a range of plant pests and pathogens (Barragán-Fonseca et al. 2022).

Chitin and chitosan derived from insect frass have been identified as effective elicitors in agriculture (Jamiołkowska 2020, Zheng et al. 2020, Meena et al. 2022, Poveda and Díez-Méndez 2023). These compounds can enhance plant resistance by triggering defense responses regulated by key plant hormones, including salicylic acid, jasmonic acid, and ethylene (Poveda 2020). Additionally, volatile fatty acids present in *T. molitor* frass have been found to act as natural repellents, discouraging larvae of the same species from aggregating in large numbers (Weaver et al. 1990).

Summarizing, in alignment with circular economy principles, *T. molitor* frass has emerged as a promising biological soil improver. Indeed, several studies have highlighted its positive effects on soil fertility (Carvalho 2017, Menéndez and Garcia-Fraile 2017, Shankar and Shikha 2017, Blakstad et al. 2023), although the research on its impact on different plant species is necessary to determine optimal application doses and timing tailored to specific crop requirements (Fuertes-Mendizábal et al. 2023). However, the research on frass effectiveness in enhancing crop yield and as biotic alleviator remain still limited.

Managing insect pests is a major challenge in agriculture; and whiteflies are among the most destructive plant pests worldwide, infesting a wide variety of crops across nearly all cultivated regions (Mound et al. Halsey 1978, Gerling 1990). One of the most problematic species is the sweetpotato whitefly, *Bemisia tabaci* (Gennadius) (Hemiptera: Aleyrodidae) (SPW), a complex of cryptic, morphologically indistinguishable species that cause both direct and indirect damage to numerous agricultural and ornamental plants (Dinsdale et al. 2010, Tay et al. 2012).

Within this species complex, the Mediterranean (MED) and Middle East–Asia Minor 1 (MEAM1) types are especially concerning due to their rapid reproduction, high invasiveness, global spread, broad host range, efficiency in virus transmission, and strong capacity to rapidly acquire resistance to many insecticides (EFSA Panel on Plant Health 2013). Studies specifically examining the impact of *B. tabaci* MED have shown significant reductions in key morphological and physiological traits of vegetable crops such as eggplant and tomato (Farina et al. 2022). These effects include stunted plant height, reduced dry biomass, and lower indirect chlorophyll content (ICC), even under controlled conditions.

Due to the significant pressure exerted by this pest on horticultural crops, developing innovative and sustainable control strategies has become a critical priority. In line with studies aimed at finding innovative and low impact solutions for pest control, and given the potential of strengthen plants for improving pest management in agriculture, a bio-oil was recently produced through pyrolysis of frass and tested on the larvae of *Plodia interpunctella* (Hübner) (Lepidoptera: Pyralidae) and *Tribolium castaneum* (Herbst) (Coleoptera: Tenebrionidae). The results showed a significant lethal effect on the first species, along with a strong repellent effect observed against both insect pests (Urrutia et al. 2023). But no studies are available so far on potential effects of frass or frass derivates on sap sucking insects and especially on *B. tabaci*.

Against this backdrop, the present study aimed to evaluate the effects of yellow mealworm frass as a plant growth promoter, assessing its impact under 3 different application doses on the morphological and physiological traits of tomato, eggplant, and sweet pepper plants. Additionally, the study explored its potential role as a phytofortificant in mitigating damage caused by the SPW. Incorporating frass in agricultural practices may offer a sustainable alternative to chemical inputs, helping to reduce production costs and minimize environmental impact.

## Materials and Methods

### Frass

*Tenebrio molitor* frass (MWF) was supplied in powdered form from a mealworm colony maintained in the Applied Entomology laboratories of the Department of Agriculture, Food and Environment (Di3A) at the University of Catania. The insects were reared under controlled environmental conditions (T = 27 ± 2 °C, RH = 65 ± 5%, and photoperiod of 14 L:10D) and fed a diet of wheat flour and carrots.

To collect the frass, larvae at various developmental stages were first separated using sieves of different mesh sizes. Once the larvae were completely removed, the remaining material was collected and stored in plastic containers for use at 4 °C, following the procedure used by Foscari (Foscari et al. 2024).

### Physicochemical Analysis of Frass

The frass was characterized for pH, electrical conductivity (EC), organic carbon, total Kjeldahl nitrogen (TKN), total phosphorus (TP) and potassium (K^+^).

To determine the pH of the frass, 10.0 g of sample was weighed into a 50 ml polyethylene bottle and 25 ml of distilled water was added to give a sample: water ratio of 1:2.5 (w/v) (Smith and Doran 1997). The mixture was shaken thoroughly for 30 min, left to stand for a further 30 min and then the pH was measured using an XS 80 Pro pH meter.

Electrical conductivity was measured using the same mixture employed for pH analysis with an XS Cond 7 VIO conductivity meter (Smith and Doran 1997).

Organic carbon content was determined following the method Springer and Klee (Astori et al. 1994). Briefly, 1.0 g of sample was placed in a 500 ml Erlenmeyer flask, followed by 10 ml of 0.167 M K_2_Cr_2_O_7_. The flask was gently swirled to disperse the soil, then 20 ml of concentrated H_2_SO_4_ was rapidly added. The mixture was subsequently swirled, first gently and then vigorously, for a total of 1 min. To minimize heat loss, the flask was left standing on an insulated surface in a fume hood for 30 min. Afterwards, 200 ml of distilled water was added and the suspension filtered using acid-resistant filter paper (Whatman No. 540). Three drops of o-phenanthroline indicator were then added and the solution was titrated with 0.5 M (NH_4_)_2_Fe(SO_4_)_2_·6H_2_O.

TKN was measured using in according to Nelson and Sommers (Nelson and Sommers 1980) with a few modifications. First, 5.0 g of sample was weighed into a test tube. Then, 2 catalyst tablets ST, 7 ml of concentrated H_2_SO_4_ were added and 5 ml of H _2_O_2_ 35%. The mixture was then digested at 420 °C for 30 min and cooled to 50 °C to 60 °C. The samples were then distilled using Method No. 26 of the VELP UDK 130 A system. During distillation, excess 35% NaOH was added to convert ${\text{NH}}_{4}^{+}$ to NH_3_, which was collected in a 4% H_3_BO_3_ receiving solution. To quantify the ammonia, 10 drops of Tashiro’s indicator solution (0.75 g/L methyl red sodium salt and 0.375 g/L methylene blue in 50% ethanol) were added to each sample. This was followed by titration with 0.2 N HCl until the endpoint was reached.

Total phosphorus (TP) was determined using the Olsen method (Olsen et al. 1954). In detail, 1.0 g of sample was weighed into a 50 ml Erlenmeyer flask. To ensure a colourless filtrate, 200 mg of activated charcoal was added to each flask. Then, 20 ml of extracting solution (NaHCO_3_ 0.5M) was added and the mixture shaken at 200 rpm for 30 min at room temperature. The extracts were then filtered through Whatman No. 42 filter paper. Color development was measured by spectrophotometry at 720 nm using a blank and standards prepared in the Olsen extracting solution (Jasco V730).

The potassium content in biomass samples was determined following wet acid digestion and subsequent analysis by flame photometry (Allen et al. 1974). Briefly, 0.5 to 1.0 g of oven-dried and finely ground biomass was accurately weighed into digestion tubes. Samples were digested using a 4:1 mixture of concentrated nitric acid (HNO_3_) and perchloric acid (HClO_4_). The mixture was heated gradually until a clear solution was obtained, indicating complete mineralization of organic matter. After cooling, the digest was quantitatively transferred to a volumetric flask and diluted to a final volume of 50 ml with deionized water. Potassium concentration was then measured using a calibrated flame photometer, with standard potassium solutions used to generate the calibration curve.

### Phytotoxicity Test of Frass

Tests on phytotoxic effects of frass treatments have been conducted on cress seeds (*Lepidium sativum* L.) and 2 germination indices were calculated, as described below.

The germination percentage (GP) was calculated for each treatment as the percentage of seeds that germinated relative to the total number of seeds sown (*n* = 20):


$$\mathrm{GP}\;=\frac{(\mathrm{Number}\;\mathrm{of}\;\mathrm{germinated}\;\mathrm{seeds})\;}{\left(\mathrm{Total}\;\mathrm{number}\;\mathrm{of}\;\mathrm{seeds}\;\mathrm{used}\;\mathrm{in}\;\mathrm{the}\;\mathrm{bioassay}\right)}\;\times \;100$$

The germination index (GI) was calculated to evaluate the effects of frass treatments on seed germination and to assess the overall response variability across different frass application doses, using the following formula:


$$\mathrm{GI}\;=\frac{\left(\mathrm{Nt}\;\times \;\mathrm{Lt}\right)}{\left(\mathrm{Nc}\;\times \;\mathrm{Lc}\right)}\;\times \;100$$

where *N*_t_ and *N*_c_ represent the mean number of germinated seeds in the treated (t) and control (c) groups, respectively, and *L*_t_ and *L*_c_ refer to the average radicle lengths of germinated seeds in the treated and control groups, respectively.

Phytotoxicity tests were performed using aqueous frass extracts at 3 concentrations: 2%, 4%, and 6% (v/v). To prepare the extracts, frass was mixed with sea sand (extra pure reagent grade), an inert substrate chosen to eliminate the potential influence of other growing media. The aqueous extracts were prepared following the ratio 1:2.5 (w/v).

### Plants

Young plants of tomato (*Solanum lycopersicum* L. cv. ‘Creativo’), eggplant (S*olanum melongena* L. cv. ‘Velia F1’), and sweet pepper (*Capsicum annuum* L. cv. ‘Altea’) were grown from seeds germinated in polystyrene trays in the nursery. Seedlings were then individually transplanted into 4-liter plastic pots containing a 1:1 (v/v) mixture of soil and peat.

At the time of transplanting, different fertilization treatments were applied, with 6 plants per treatment group, each representing a replicate:

At the time of transplanting, different fertilization treatments were applied at 60 plants/species (total of 180 plants):

- C-: negative control—soil and peat only, no mealworm frass;
- 2%: soil and peat amended with 2% (v/v) MWF;
- 4%: soil and peat amended with 4% (v/v) MWF;
- 6%: soil and peat amended with 6% (v/v) MWF;
- C+: positive control—soil, peat, and mineral fertilizer (Yara-Mila Nutriplus 11-15-15, applied at 9 g per plant).

Plants were maintained under these conditions for 14 d before *B. tabaci* release.

### Plant Measurements

To assess the effects of frass on the growth of tomato, eggplant, and sweet pepper, plant height (*ht*), number of leaves (*ln*), ICC, leaf area (*la*), and the fresh (*fwt*) and dry weight (*dwt*) of the plant (roots and shoots) were measured on the plants arranged to conduct the choice test (see par. Choice test), which more realistically reproduces natural growth conditions. Plant height, expressed in centimeters, was measured with a ruler; *fwt* was expressed in grams, cutting and weighing shoots and roots with a high-precision balance (ORMA BC 1000, Orma srl, Milan, Italy; resolution 0.1 g). Regarding *dwt*, the biomass was oven-dried (Thermo Fisher Scientific, Langenselbold, Germany, Heratherm OGS100) at 65 °C, until a constant weight was reached in 3 d and, finally, weighed and expressed in grams, as well. The ICC measurements were performed using a Soil Plant Analysis Development (SPAD-502, Minolta, Sakai, Osaka, Japan) chlorophyll meter, expressed in SPAD units, on 3 leaves per plant, which were at the principal growth stage of leaf development (ie tomato stage 19, sweet pepper stage 19, eggplant stage 16), according to the BBCH scale. The plant leaf area, expressed in cm^2^, was determined by ImageJ software (Wayne Rasband-Services Branch, National Institute of Mental Health, Bethesda, Maryland, United States), which processed the photos shot by a digital camera (PIXPRO FZ45, Eastman Kodak Company, Rochester, New York, United States).

Non-destructive measurements, ie *ht*, *ln*, and ICC, were recorded at 14, 28, and 49 d after transplanting (DAT), *la* was determined at 28, 35, and 49 DAT, while *fwt* and *dwt* were evaluated only at the end of experiment (49 DAT).

In this study, only the results obtained at 49 DAT are presented and discussed, whereas data from earlier measurements are reported in the Supplementary Materials (Supplementary Figs S1–S12).

### Bemisia tabaci

The rearing population of *B. tabaci* (SPW) was collected from a greenhouse-grown eggplant crop in southeastern Sicily (province of Ragusa, 36.97134 lat.; 14.424505 long.) and maintained on eggplant plants in the insectarium of the Department of Agriculture, Food and Environment (University of Catania, Italy) in insect-proof BugDorm cages (MegaView Science, Taichung, Taiwan), under controlled environmental conditions (*T* = 25 ± 2 °C, RH = 65 ± 5%, and photoperiod of 14 L:10D h).

### Molecular Identification of *B. tabaci*

Before running the experiment, DNA was extracted from approximately 30 SPW adults collected from the laboratory colony to confirm that it consistently consisted of *B. tabaci* MED. To this aim, the total DNA was extracted from single individuals (De Barro and Driver 1997, Walsh et al. 2013). The mitochondrial cytochrome oxidase I (mt COI) gene (about 710 bp) was amplified using the primers LCO1490 and HCO2198 (Folmer et al., 1994, Gautam et al. 2020, Song et al. 2021). For each specimen, the 10 μl reaction volume contained 5 μl of FailSafe 2× PreMixes buffers (Lucigen, Middleton, Wisconsin, United States), 3.75 μl of DNA, 0.25 μl of taq polymerase, and 0.5 μl of each forward and reverse primer. The PCR was completed with initial denaturation at 96 °C for 5 min, followed by 35 cycles, each consisting of denaturation for 45 s at 96 °C, annealing for 60 s at 45 °C, with final extension for 1 min at 72 °C, followed by final extension for 10 min, at 72 °C. PCR-amplified products (10 μl) were visualized with 0.9% agar-gel electrophoresis (5 μl), and those with the target fragment were carefully chosen for sequencing. Positively amplified DNA (5 μl) was purified and sequenced by BMR genomics.

### Semi-Field Trials

Semi-field trials were carried out in the experimental greenhouse at the Azienda Agraria Sperimentale (37.407367 lat.; 15.060784 long.) of the University of Catania (Italy) from October to December 2024. The trials included both choice and no-choice tests on all 3 plant species mentioned above, once they had developed 4 fully expanded leaves; the choice test evaluated SPW oviposition and colonization preferences, while the no-choice test assessed how suitable the treated plants were for supporting the development of different SPW life stages.

### Choice Test

The preference of *B. tabaci* among different plant species exposed to various treatments was assessed through the choice test. The experiment followed a completely randomized block design with the 3 plant species and 5 treatments (C-, MWF 2%, MWF 4%, MWF 6%, C+). Plants were irrigated daily and randomly arranged in metal tables (3.5 m × 1.5 m) covered with an anti-insect net, with each representing an experimental unit. In total, 15 treatments (ie 5 treatments per plant species) and 6 replicates per treatment were considered.

Six hundred adults of SPW, previously collected from the insectarium, were released within each experimental unit and after 24, 48, and 72 h their number/cm^2^ was counted on 3 leaves/plant from 6 to 8 am, to determine their settling preference. Moreover at 28, 35, and 49 DAT, oviposition, and colonization preferences were assessed by recording the number of eggs, nymphs and subpupae of the whitefly on 3 leaves/plant, using a microscope (Olympus mod. SZX-ILLK200).

### No-Choice Test

With the no-choice test, the different treated plant species were singly isolated and exposed to the whitefly to assess the plant’s suitability in supporting the development of the different instars of the insect.

The test was carried out in a completely randomized design with the 3 plant species, each treated with the aforementioned different applications (C-, 2%, 4%, 6%, C+) and representing a replicate. Plants were irrigated daily and individually arranged in metal cylinders (20 cm diameter, 90 cm height) covered with an anti-insect net, carrying out a total of 15 treatments (5 treatments per plant species) and 6 replicates per treatment

Forty SPW adults, previously collected from the insectarium, were released per replicate and, at 28 and 49 DAT, the number of eggs, nymphs, and subpupae on 3 leaves/plant were counted, when present.

### Statistical Analysis

Statistica 7.0 software was adopted for the analysis of the following data: pH and E.C. values on different growing substrates, germination index, morphological and physiological parameters of the plants, and data relating to SPW infestation. A 1-way ANOVA followed by Tukey’s HSD post hoc test (*P *< 0.05) was applied for normally distributed and homoscedastic data, whereas the nonparametric Kruskal–Wallis *H* test followed by Dunn’s post hoc test (*P* < 0.05) was used for non-normally distributed or heteroscedastic data. For the choice test, the number of SPW adults per cm^2^ was assessed separately at 24, 48, and 72 h after infestation. Likewise, the numbers of eggs, nymphs, and subpupae per cm^2^ were analyzed separately at 28, 35, and 49 d after transplanting (DAT). At the end of the experiment (49 DAT), the total number of specimens was also assessed. In the no-choice test, the same analytical procedure was applied, with the numbers of SPW eggs, nymphs, and subpupae per cm^2^, separately evaluated at 28 and 49 DAT.

## Results

### Physicochemical Properties of Frass and Treated Substrates

The chemical composition of the frass revealed a high organic carbon content (56.02 ± 0.01%) and a total nitrogen content of 3.81 ± 0.02%, resulting in a relatively low C: N ratio (∼14.7:1). Frass also contained 0.49 ± 0.04% potassium (K^+^), 0.13 ± 0.01% phosphorus (P), 9.68 ± 0.15% moisture, and 3.41 ± 0.17% ash content. The frass also showed an acidic pH (5.66 ± 0.02) and a high electrical conductivity (E.C) of 6225 ± 545.0 µS/cm (Table 1).

**Table 1.** Chemical and physical characteristics of frass used during the experiments

Moisture (%)	Ash (%)	O.C. (%)	K^+^ (%)	N tot (%)	P (%)	pH	E.C. µS/cm
**9.68 ± 0.15**	3.41 ± 0.17	56.02 ± 0.01	0.49 ± 0.04	3.81 ± 0.02	0.13 ± 0.01	5.66 ± 0.02	6,225 ± 545.0

Values are expressed as mean ± SE and reported as percentage (%). The parameters measured include moisture and ash content, organic carbon (O.C.), total nitrogen (N tot), potassium (K^+^), and phosphorus (P), pH, and electrical conductivity (E.C.).

The amendment of the growing substrates with frass modified both pH and E.C. values. Specifically, in control soil (C−), pH decreased following frass incorporation; conversely, the addition of frass to sea sand resulted in a significant increase in pH, independent of application rate (*F *= 8.77, df = 7.10, *P *≤ 0.001). Interestingly, mixing the frass to the negative control, the E.C. values increased significantly, as it was obtained using the mineral fertilizer (C+) (*F* = 1107.13, df = 7.10, *P *≤ 0.001) (Table 2).

**Table 2.** pH and E.C. of the experimental soil and frass, and frass-based substrates

	pH	E.C. µS/cm
**C- (negative control)**	5.51 ± 0.15ab	144.8 ± 0.9c
**C+ (positive control)**	5.50 ± 0.25ab	375.0 ± 12.5b
**Soil + 2% Frass**	5.15 ± 0.04b	349.5 ± 1.5b
**Soil + 4% Frass**	4.93 ± 0.10b	341 ± 8.0b
**Soil + 6% Frass**	5.2 ± 0.70b	385.5 ± 1.5b
**Sea sand + 2% Frass**	6.17 ± 0.11a	392 ± 10.0b
**Sea sand + 4% Frass**	6.04 ± 0.02a	624 ± 24.0ab
**Sea sand + 6% Frass**	6.04 ± 0.005a	826 ± 2.0a

Values are reported as mean ± SE. Treatments included untreated soil, soil amended with frass at 2%, 4%, and 6%, and sea sand amended with frass at the same application rates. Different letters indicate significant differences (Tukey’s test, *P* < 0.05).

### Phytotoxicity Test

**Fig. 1.**
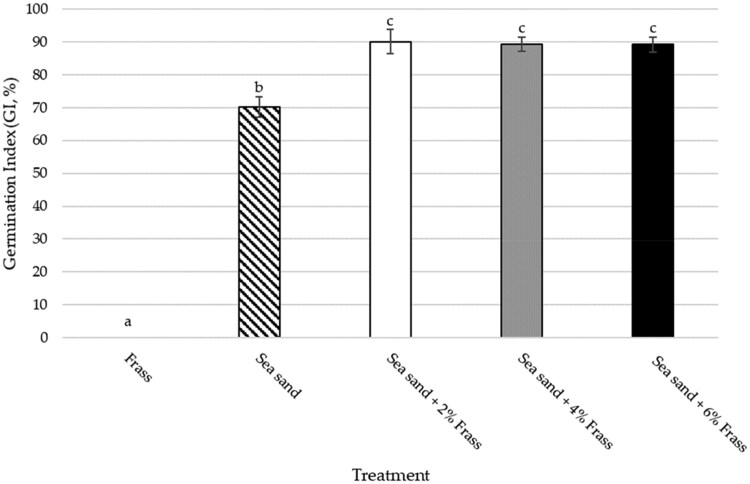
Germination index of seeds (*L. sativum)* treated with different concentrations of frass. Data (± SE) about treatment performance are the means of 3 replicates. Different letters indicate significant differences (Tukey’s test, *P *< 0.001).

As shown in Fig. 1, the percentage of germinated seeds varied significantly among the treatments (*F *= 281.50, df = 4,10, *P *≤ 0.001). The frass alone exhibited the lowest germination rate, with an average close to 0%, indicating strong inhibitory effects when frass is used alone. In contrast, the sea sand alone showed a considerably higher germination rate (around 70%).

All treatments involving frass mixed with sea sand resulted in high germination percentages, ranging from approximately 85% to 90%, with no significant differences among them. These values are higher than the sand alone, suggesting that when frass is appropriately diluted with soil, it can enhance seed germination rather than inhibit it.

### Plant Measurements

The application of MWF significantly affected the vegetative growth and physiological parameters of tomato (*Solanum lycopersicum* L. cv. ‘Creativo’), sweet pepper (*Capsicum annuum* cv. ‘Altea’), and eggplant (*Solanum melongena* L. cv. ‘Velia F1’), with different responses depending on the applied dose.

#### Growth and Physiological Parameters of Tomato Plants

At 49 DAT, tomato plants treated with frass exhibited improved growth performance, particularly in leaf number (*ln*), leaf area (*la*), ICC, and plant biomass (*fwt* and *dwt*), except plant height (*ht*), which was not significantly affected.

**Fig. 2.**
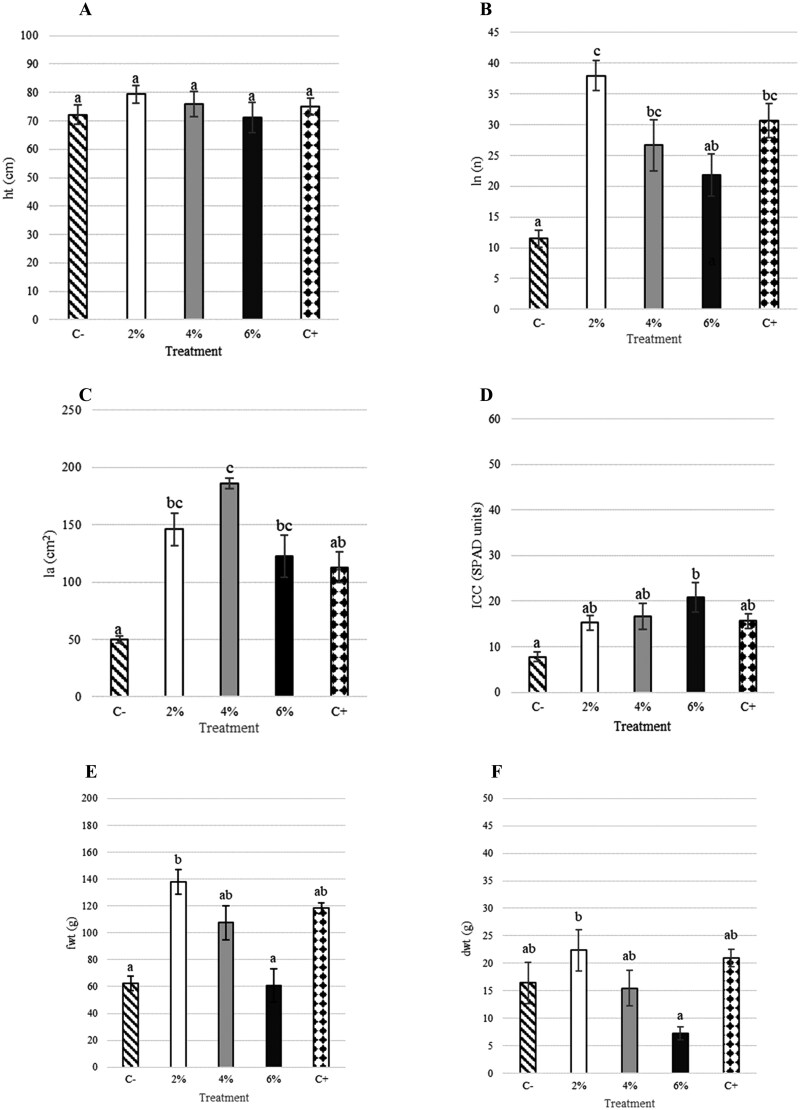
Effect of MWF application on tomato plant growth. Mean values (± SE) of plant height ht (A), leaf number ln (B), leaf area la (C), chlorophyll indirect content ICC (D), plant fresh weight fwt (E) and plant dry weight dwt (F) of tomato plants measured 49 d after transplanting. Treatments: C-: untreated control; 2%: MWF at 2%; 4%: MWF at 4%; 6%: MWF at 6%; C+: positive control (mineral commercial fertilizer). Different letters indicate significant differences (Tukey’s test, *P *< 0.05).

In terms of leaf number, the 2% frass treatment (38.00 ± 2.45 leaves), followed by C+ and 4% frass treatment (30.67 ± 2.80 and 26.67 ± 4.18 leaves, respectively), determined a significantly positive effect when compared with the negative control (11.50 ± 1.38 leaves) (*F *= 10.89, df = 4,25, *P *≤ 0.001); this positive effect decreased significantly anyway as the doses increased (Fig. 2B). Plants treated with 4% frass also displayed a statistically significant effect on the *la* value (185.74 ± 4.55 cm^2^) compared to both untreated plants (49.81 ± 2.86 cm^2^) and positive control (112.78 ± 13.48 cm^2^) (*F *= 16.79, df = 4,25, *P *< 0.001) (Fig. 2C). ICC increased in frass-treated plants, with the highest values recorded in the 6% treatment (20.81 ± 3.22 SPAD units), significantly differing from the negative control (7.8 ± 1.12 SPAD units) (*F *= 4.37, df = 4,25, *P *= 0.008) (Fig. 2D).

Regarding plant biomass, the 2% frass treatment significantly increased *fwt* (137.69 ± 9.15 g) compared to the negative control (62.48 ± 5.43 g) and 6% frass treatment (60.89 ± 12.24 g) (*F *= 16.32, df = 4,25, *P *< 0.001) (Fig. 2E). A similar trend was observed for *dwt*, with the 2% frass treatment differing significantly from the 6% treatment (7.26 ± 1.16 g) (*F *= 4.65, df = 4,25, *P *< 0.001) and showing the highest value (22.38 ± 3.78 g) compared to the remaining other treatments (Fig. 2F).

#### Growth and Physiological Parameters of Sweet Pepper Plants

From comparison with C-, it emerged how MWF applications led to interesting improvements across almost all considered parameters of sweet pepper plants.

**Fig. 3.**
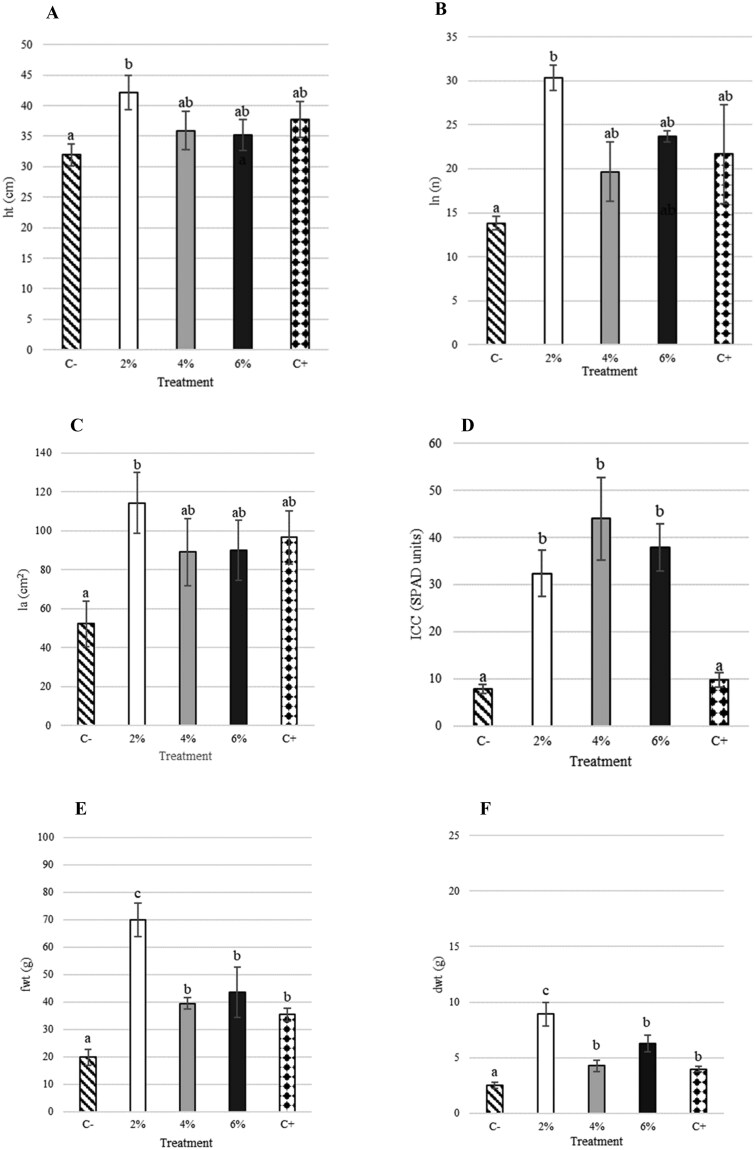
Effect of MWF application on sweet pepper plant growth. Mean values (± SE) of plant height ht (A), leaf number ln (B), leaf area la (C), chlorophyll indirect content ICC (D), plant fresh weight fwt (E) and plant dry weight dwt (F) of pepper plants measured 49 d after transplanting. Treatments: C-: untreated control; 2%: MWF at 2%; 4%: MWF at 4%; 6%: MWF at 6%; C+: positive control (mineral commercial fertilizer). Different letters indicate significant differences (Tukey’s test, *P *< 0.05).

Indeed, the 2% frass treatment significantly increased *ht* (*F *= 2.92, df = 4,25, *p *= 0.04), *ln* (*F* = 5.89, df = 4,25, *P *= 0.002), and *la* (*F *= 3.48, df = 4,25, *P *= 0.022), reaching 42.17 ± 2.84 cm, 30.33 ± 1.47 leaves, and 114.23 ± 15.59 cm^2^, respectively, compared with 31.92 ± 1.84 cm, 13.83 ± 0.74 leaves, and 52.16 ± 11.72 cm^2^ in the negative control (Fig. 3A–C).

Regarding ICC (Fig. 3D), all frass treatments exhibited significantly higher mean values than the negative and positive control (*F *= 15.78, df = 4,25, *P *< 0.001) Finally, all frass treatments significantly enhanced plant biomass (Fig. 3E and F). The 2% treatment exhibited the highest fresh (69.85 ± 6.03 g) and dry weights (8.92 ± 1.04 g), significantly higher than all other treatments, and particularly different from the negative control (*F *= 15.74, df = 4,25, *P *< 0.001 and *F *= 22.31, df = 4,25, *P *< 0.001, respectively).

#### Growth and Physiological Parameters of Eggplant Plants

**Fig. 4.**
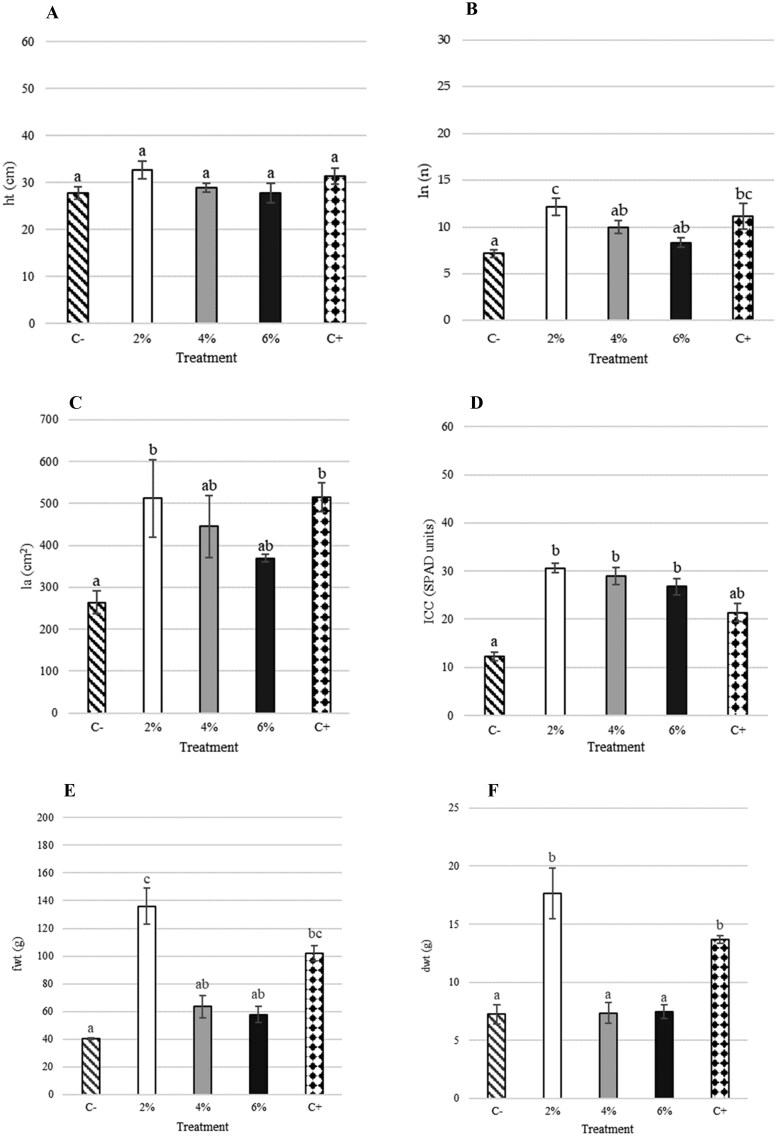
Effect of MWF application on eggplant plant growth. Mean values (± SE) of plant height ht (A), leaf number ln (B), leaf area la (C), chlorophyll indirect content ICC (D), plant fresh weight fwt (E) and plant dry weight dwt (F) of eggplant plants measured 49 d after transplanting. Treatments: C-: untreated control; 2%: MWF at 2%; 4%: MWF at 4%; 6%: MWF at 6%; C+: positive control (mineral commercial fertilizer). Different letters indicate significant differences (Tukey’s test, *P *< 0.05).

On eggplant all measured parameters were significantly affected by frass application, with the exception of *ht* (Fig. 4A).

Regarding *ln*, the 2% treatment exhibited the highest mean value (12.17 ± 0.91 leaves), followed by C+ (11.17 ± 1.4 leaves), significantly exceeding the negative control (7.17 ± 0.40 leaves) (*F *= 5.66, df = 4,25, *P *= 0.002) (Fig. 4B). As for *la*, the 2% frass treatment (512.31 ± 92.73), along with the positive control (515.28 ± 33.84), showed the highest mean values, both significantly greater than the negative control (264.28 ± 27.53) (*F *= 3.50, df = 4,25, *P *= 0.021) (Fig. 4C). For ICC the frass-treated plants recorded the highest SPAD values (31.96 ± 0.93), significantly differing from the negative control (12.71 ± 0.84) (*F *= 11.02, df = 4,25, *P *< 0.001) (Fig. 4D). Regarding plant biomass, *fwt* was significantly influenced by the lowest frass application (*F *= 45.74, df = 4,25, *P *≤ 0.001): the 2% frass treatment led to the highest mean *fwt* (136.05 ± 13.01 g), followed by C+ (102.05 ± 8.63 g), and was notably greater than *fwt* of untreated plants (Fig. 4E). A similar result was observed for *dwt*, in fact the 2% frass treatment showed the highest value (17.66 ± 2.20 g), along with the positive control (13.71 ± 0.52 g), which was significantly different from the negative control (7.23 ± 0.80 g) (*F *= 21.50, df = 4,25, *P *≤ 0.001) (Fig. 4F).

### Molecular Identification of *B. tabaci*

Molecular identification confirmed that the individuals used in the experiment belonged to *B. tabaci* MED, showing more than 99% sequence identity with reference mtCOI sequences in the NCBI database. All sequences were identical to each other and were deposited in GenBank under accession number PX692955.

### Choice Test

At 24, 48, and 72 h after the release of *B. tabaci* adults, no significant differences were observed in the number of whitefly adults present on the leaves regardless both the treatments and the 3 crop species (*F *= 0.66, df = 4,25, *P *= 0.79), therefore indicating that the presence of frass did not affect the whitefly settling preferences.

Concerning the oviposition preference on tomato plants, at 28 DAT (Table 3), no significant differences were found among treatments (*F *= 1.46, df = 4,25, *P *= 0.24). Otherwise, a significant difference in the number of eggs/cm^2^ was observed on sweet pepper (*F *= 15.48, df = 4,25, *P *≤ 0.001), with the highest mean number for the 2% treatment and the lowest in the C- and C+ treatments. Similarly, on eggplants the highest number of eggs/cm^2^ was recorded when 2% of frass was incubated in the growing substrate, demonstrating a significant difference in the oviposition preference of the whitefly (*F *= 9.01, df = 4,25, *P *≤ 0.001).

**Table 3.** Oviposition and colonization preferences of *B. tabaci* at 28 DAT in the choice test

Plant	Treatment	Eggs/cm^2^	Nymphs/cm^2^
**T**	C-	0.24 ± 0.07a	0.13 ± 0.05a
**T**	2%	0.26 ± 0.06a	0.42 ± 0.11ab
**T**	4%	0.26 ± 0.05a	0.28 ± 0.07ab
**T**	6%	0.23 ± 0.05a	0.47 ± 0.11b
**T**	C+	0.10 ± 0.02a	0.18 ± 0.04ab
**P**	C-	0.17 ± 0.04ab	0.20 ± 0.04ab
**P**	2%	1.00 ± 0.22c	1.05 ± 0.15c
**P**	4%	0.57 ± 0.12bc	0.46 ± 0.24bc
**P**	6%	0.25 ± 0.04abc	0.35 ± 0.34abc
**P**	C+	0.10 ± 0.02a	0.08 ± 0.06a
**E**	C-	0.24 ± 0.06a	0.30 ± 0.07a
**E**	2%	1.22 ± 0.29b	0.93 ± 0.16b
**E**	4%	0.26 ± 0.08a	0.43 ± 0.07a
**E**	6%	0.55 ± 0.16a	0.66 ± 0.09a
**E**	C+	0.24 ± 0.06a	0.19 ± 0.03a

Mean number of eggs and nymphs (± SE) on tomato (T), sweet pepper (P), and eggplant (E) grown on frass-treated substrates (2%, 4%, 6%), untreated control (C−), and mineral fertilizer control (C+). Different letters indicate significant differences (Tukey’s test, *P *< 0.05).

Again, the presence of the frass significantly affected the number of nymphs/cm^2^, on tomato (*F *= 3.80, df = 4,25, *P *= 0.02), sweet pepper (*F *= 11.75, df = 4,25, *P *≤ 0.001), and eggplant (*F *= 11,89, df = 4,25, *P *≤ 0.001) crops. In particular, the 2% treatment allowed to record the highest mean number, with 1.05 nymphs/cm^2^ and 0.93 nymphs/cm^2^ on sweet pepper and eggplant respectively (Table 3).

At 35 DAT, nymphs were detected on all tested plants with variable density across plant species and treatments. Indeed, no significant differences were observed in their number on tomato plants (*F *= 0.87, df = 4,25, *P *= 0.49), whereas a significantly higher number of nymphs was recorded on sweet pepper plants in the 6% and 2% frass treatments, followed by the 4% treatment (*F *= 9.90, df = 4,25, *P *≤ 0.001) (Table 4). Finally, on eggplant plants, a significantly higher number of nymphs was recorded in the untreated control, followed by the 2% frass treatment (*F *= 10.25, df = 4,25, *P *≤ 0.001) (Table 4).

**Table 4.** Colonization preferences of *B. tabaci* at 35 DAT in the choice test

Plant	Treatment	Nymphs/cm²
**T**	C-	0.38 ± 0.07a
**T**	2%	0.36 ± 0.10a
**T**	4%	0.24 ± 0.06a
**T**	6%	0.29 ± 0.07a
**T**	C+	0.40 ± 0.07a
**P**	C-	0.24 ± 0.04ab
**P**	2%	0.91 ± 0.15c
**P**	4%	0.88 ± 0.24abc
**P**	6%	1.38 ± 0.34c
**P**	C+	0.16 ± 0.06a
**E**	C-	1.88 ± 0.29c
**E**	2%	1.71 ± 0.18bc
**E**	4%	1.04 ± 0.12ab
**E**	6%	0.66 ± 0.12a
**E**	C+	1.11 ± 0.10ab

Mean number of nymphs (± SE) on tomato, sweet pepper, and eggplant grown on frass-treated substrates (2%, 4%, 6%), untreated control (C−), and mineral fertilizer control (C+). Different letters indicate significant differences (Tukey’s test, *P*< 0.05).

At 49 DAT (ie 35 d after the insect release), eggs, nymphs, and subpupae were present on all plants and their numbers were not affected by the presence of the frass on tomato (eggs: *F *= 1.29, df = 4,25, *P *= 0.30; nymphs: *F *= 1.41, df = 4,25, *P *= 0.26; subpupae: *F* = 2.14, df = 4,25, *P *= 0.11) (Table 5), and sweet pepper plants (eggs: *F* = 1.13, df = 4,25, *P *= 0.36; nymphs: *F* = 1.54, df = 4,25, *P *= 0.22; subpupae: *F *= 0.85, df = 4,25, *P *= 0.5) (Table 5). In contrast, significant differences were detected in eggplant, where both the mean number of eggs and the number of subpupae varied significantly among treatments (*F *= 7.80, df = 4,25, *P *≤ 0.001 and *F *= 7.47, df = 4,25, *P *≤ 0.001, respectively) likely due to newly emerged adults of *B. tabaci*. However, no significant differences were found in the number of nymphs (*F *= 3.16, df= 4,25, *P *= 0.03) (Table 5).

Looking at the data on the total number of specimens recorded at the end of the trial, frass treatments did not influence pest density on tomato (*F *= 1.34, df = 4,25, *P *= 0.28) and sweet pepper plants (*F *= 1.28, df = 4,25, *P *= 0.30) (Table 5). In contrast, eggplant plants were positively affected by the presence of frass, as well as by the C+ treatment, and a low pest density occurred in all these treatments (*F *= 5.00, df = 4,25, *P *= 0.004) (Table 5).

**Table 5.** Colonization preferences of *B. tabaci* at 49 DAT in the choice test

Plant	Treatment	Eggs/cm²	Nymphs/cm²	Subpupae/cm²	Total instars/cm²
**T**	C-	0.14 ± 0.02a	0.37 ± 0.07a	0.01 ± 0.01a	1.05 ± 0.06a
**T**	2%	0.12 ± 0.01a	0.17 ± 0.03a	0.00 ± 0.00a	0.57 ± 0.04a
**T**	4%	0.18 ± 0.02a	0.27 ± 0.05a	0.04 ± 0.02a	0.99 ± 0.06a
**T**	6%	0.15 ± 0.04a	0.33 ± 0.10a	0,01 ± 0.01a	0.97 ± 0.14a
**T**	C+	0.18 ± 0.02a	0.22 ± 0.06a	0.00 ± 0.00a	0.81 ± 0.08a
**P**	C-	0.02 ± 0.02a	0.27 ± 0.13a	0.22 ± 0.10a	0.71 ± 0.29a
**P**	2%	0.06 ± 0.04a	0.24 ± 0.11a	0.12 ± 0.06a	0.49 ± 0.21a
**P**	4%	0.20 ± 0.12a	0.69 ± 0.23a	0.24 ± 0.10a	1.29 ± 0.49a
**P**	6%	0.13 ± 0.06a	0.46 ± 0.29a	0.24 ± 0.11a	0.92 ± 0.42a
**P**	C+	0.08 ± 0.03a	0.11 ± 0.04a	0.06 ± 0.03a	0.33 ± 0.09a
**E**	C-	0.38 ± 0.06 b	1.05 ± 0.20a	1.12 ± 0.20 b	2.54 ± 0.47 b
**E**	2%	0.28 ± 0.05 b	0.57 ± 0.06a	0.33 ± 0.07a	1.17 ± 0.15a
**E**	4%	0.28 ± 0.05 b	0.57 ± 0.08a	0.33 ± 0.10a	1.17 ± 0.21a
**E**	6%	0.50 ± 0.12 b	0.56 ± 0.12a	0.43 ± 0.10a	1.49 ± 0.28ab
**E**	C+	0.03 ± 0.02a	0.84 ± 0.12a	0.61 ± 0.10ab	1.48 ± 0.24ab

Mean number of eggs, nymphs, subpupae and total instars (± SE) on tomato (A), sweet pepper (B), and eggplant (C) grown on frass-treated substrates (2%, 4%, 6%), untreated control (C−), and mineral fertilizer control (C+). Different letters indicate significant differences (Tukey’s test, *P *< 0.05).

### No-Choice Test

At 28 DAT, the highest number of eggs on tomato plants was recorded in the C+ treatment, with a mean of 0.31 eggs/cm^2^ (*F *= 27.57, df = 4,25, *P *≤ 0.001) (Table 6), while on sweet pepper, the 6% treatment was the most infested, with 1.40 eggs/cm^2^ (*F *= 19.22, df = 4,25, *P *≤ 0.001) (Table 6). Instead, in eggplant, the C− treatment exhibited the highest egg density (1.11 eggs/cm^2^) and differed significantly from the other treatments (*F *= 7.92, df = 4,25, *P *≤ 0.001).

**Table 6.** Effect of treatments on *B. tabaci* oviposition and development at 28 DAT in the no-choice test

Plant	Treatment	Eggs/cm^2^	Nymphs/cm^2^	Total instars/cm^2^
**T**	C-	0.19 ± 0.01b	0.05 ± 0.01a	0.24 ± 0.02a
**T**	2%	0.12 ± 0.02ab	0.08 ± 0.01ab	0.20 ± 0.03a
**T**	4%	0.14 ± 0.01ab	0.11 ± 0.02b	0.26 ± 0.02a
**T**	6%	0.09 ± 0.02a	0.20 ± 0.02c	0.40 ± 0.03b
**T**	C+	0.31 ± 0.02c	0.13 ± 0.02b	0.44 ± 0.03b
**P**	C-	0.28 ± 0.08a	0.24 ± 0.06a	0.52 ± 0.11a
**P**	2%	0.81 ± 0.05b	0.68 ± 0.15ab	1.53 ± 0.18b
**P**	4%	0.64 ± 0.10ab	0.87 ± 0.09b	1.53 ± 0.19b
**P**	6%	1.40 ± 0.13c	1.42 ± 0.17c	2.83 ± 0.15c
**P**	C+	0.46 ± 0.12ab	0.51 ± 0.08ab	0.98 ± 0.15ab
**E**	C-	1.11 ± 0.18 b	0.41 ± 0.13ab	1.63 ± 0.28b
**E**	2%	0.49 ± 0.09a	0.45 ± 0.10b	1.00 ± 0.23ab
**E**	4%	0.45 ± 0.09a	0.29 ± 0.04ab	0.74 ± 0.12a
**E**	6%	0.63 ± 0.06ab	0.44 ± 0.08b	1.09 ± 0.14ab
**E**	C+	0.34 ± 0.09a	0.11 ± 0.0a	0.44 ± 0.12a

Mean number (± SE) of eggs, nymphs and total instars (± SE) on tomato (T), sweet pepper (P), and eggplant (E) recorded at 28 DAT in the no-choice test. Treatments: C-: untreated control; 2%: MWF at 2%; 4%: MWF at 4%; 6%: MWF at 6%; C+: positive control (mineral commercial fertilizer). Different letters in the same column indicate significant differences (Tukey’s test, *P *< 0.05).

Nymph density at 28 DAT also differed significantly among treatments. On tomato, the 6% treatment was the most infested and differed significantly from the other treatments (*F *= 13.95, df = 4,25, *P *≤ 0.001) (Table 6). On sweet pepper, the 6% treatment was again the most infested, significantly differing from the other treatments (*F *= 13.68, df = 4,25, *P *≤ 0.001) (Table 6), while on eggplant, the 2% and 6% treatments showed the highest nymph density (*F *= 2.87, df = 4,25, *P *= 0.044) compared to the lowest values recorded on C+ plants (Table 6).

At 49 DAT, eggs, nymphs, and subpupae were present on all plants, as observed in choice test at the same time. In tomato crops (Table 7), a significant difference was detected in the number of eggs (*F *= 10.26, df = 4,25, *P *≤ 0.001), with the 2% frass treatment showing the highest number.

**Table 7.** Effect of treatments on *B. tabaci* oviposition and development at 49 DAT in the no-choice test

Plant	Treatment	Eggs/cm²	Nymphs/cm²	Subpupae/cm²	Total instars/cm²
**T**	C-	0.10 ± 0.01a	0.66 ± 0.09a	0	0.76 ± 0.10a
**T**	2%	0.21 ± 0.02c	0.74 ± 0.07a	0.01 ± 0.01a	0.96 ± 0.08a
**T**	4%	0.13 ± 0.03ab	0.59 ± 0.07a	0.04 ± 0.02a	0.77 ± 0.09a
**T**	6%	0.06 ± 0.01a	0.51 ± 0.06a	0.01 ± 0.00a	0.58 ± 0.06a
**T**	C+	0.15 ± 0.02ab	0.42 ± 0.13a	0.01 ± 0.01a	0.58 ± 0.15a
**P**	C-	0.32 ± 0.11a	1.02 ± 0.10a	0.50 ± 0.22a	2.01 ± 0.30a
**P**	2%	0.31 ± 0.07a	1.37 ± 0.22ab	0.95 ± 0.52a	2.67 ± 0.68a
**P**	4%	0.59 ± 0.09ab	1.84 ± 0.21b	0.34 ± 0.22a	2.81 ± 0.35a
**P**	6%	0.84 ± 0.11b	1.98 ± 0.18b	0.71 ± 0.20a	3.64 ± 0.25a
**P**	C+	0.73 ± 0.10b	1.55 ± 0.20ab	1.24 ± 0.63a	4.00 ± 1.10a
**E**	C-	0.26 ± 0.07a	1.59 ± 0.22b	2.22 ± 0.37b	4.34 ± 0.68b
**E**	2%	0.45 ± 0.13a	0.81 ± 0.05a	0.73 ± 0.30a	2.15 ± 0.34a
**E**	4%	0.53 ± 0.12a	0.49 ± 0.11a	0.36 ± 0.18a	1.40 ± 0.37a
**E**	6%	0.35 ± 0.09a	0.77 ± 0.13a	0.56 ± 0.22a	1.71 ± 0.38a
**E**	C+	0.37 ± 0.08a	0.93 ± 0.15a	0.48 ± 0.27a	1.83 ± 0.36a

Mean number (± SE) of eggs, nymphs and total instars (± SE) on tomato (T), sweet pepper (P), and eggplant (E) recorded at 49 DAT in the no-choice test. Treatments: C-: untreated control; 2%: MWF at 2%; 4%: MWF at 4%; 6%: MWF at 6%; C+: positive control (mineral commercial fertilizer). Different letters in the same column indicate significant differences (Tukey’s test, *P *< 0.05).

In the case of sweet pepper, the 6% frass treatment, together with C+ showed the highest number of *B. tabaci* eggs, with significant differences compared to C- and 2% treatments (*F *= 6.09, df= 4,25, *P *≤ 0.002). Instead, about nymphs, the 6% treatment together with the 4%, exhibited the highest number, significantly different from the untreated plants (*F *= 4.11, df = 4,25, *P *= 0.01) (Table 7).

Consistent with the results of the choice test at 49 DAT, eggplant plants—unlike the other species—exhibited a higher abundance of nymphs and subpupae (*F *= 7.93, df = 4,25, *P *≤ 0.001 and *F *= 7.65, df = 4,25, *P *≤ 0.001, respectively), with significant differences among treatments detected for both developmental stages (Table 7). The untreated plants (C-) constantly showed the highest density of *B. tabaci* developmental stages (*F *= 7.05, df = 4,25, *P *≤ 0.001) (Tables 5 and 7).

## Discussion

The results of the present investigation outline that MWF represents an effective soil conditioner for the tested Solanaceae crops, also elucidating the insect’s host preference and development under semi-field conditions.

The chemical characterization of the frass revealed appreciable contents of macronutrients, including carbon (C), nitrogen (N), phosphorus (P), and potassium (K), as well as a moderate amount of ash. These elements, especially N, P, and K, are essential for plant development and indicate that, following mineralization processes, frass could directly contribute to fulfil the nutritional requirements of several crops.

Regarding the role of MWF as a plant growth promoter, the results obtained demonstrate that frass application significantly enhanced key morphological and physiological traits across tomato, sweet pepper and eggplant.

The observed growth-promoting effects are likely linked to the nutritional composition of MWF and/or the contribution of associated microbiota (Houben et al. 2021, Poveda 2021b, Foughar et al. 2024). In fact, frass is a nutrient-rich organic amendment containing essential macro- (N, P, K) and micronutrient, thereby enhancing plant growth and soil health (Poveda 2021a, Amorim et al. 2024). In particular, the combination, in the frass used in this study, of a low C/N ratio and high organic matter content may have stimulated soil microbial activity, accelerating nitrogen mineralization and improving nutrient availability, which ultimately supported the enhanced vegetative growth observed in treated plants (Houben et al. 2021).

Frass was not phytotoxic and has improved germination. This stimulatory effect supports the hypothesis that frass may act similarly to natural compounds with hormone-like activity, exhibiting biostimulant effects at low doses, while potentially inhibiting growth at higher concentrations. Such behavior is consistent with other organic materials known to contain allelochemicals or phytohormone analogs (Fragalà et al. 2023).

Among the tested concentrations, the 2% dose generally resulted in significant improvements in plant performance across all crops, enhancing most of the considered growth parameters, and suggesting it as a potentially optimal rate under the experimental conditions. Similar positive effects of frass on plant growth were reported in the seedling production of *Allium cepa* L., *Beta vulgaris* L., and *Brassica rapa* L., where low doses of MWF improved growth and biomass (Baldacchino and Lamaj 2025). Likewise, studies on lettuce plants demonstrated the potential of frass as an organic fertilizer, supplying macronutrients and enhancing aerial biomass at low application amounts (Fuertes-Mendizábal et al. 2023). The 4% dose also showed beneficial effects, particularly on tomato and sweet pepper, although the response was more variable compared to the consistent effect observed at 2%. Similar positive effects were reported for other crops, such as zucchini, where the application of 4% frass improved vegetative growth parameters and increased yield by 62% compared to the control (Zim et al. 2022). Conversely, the 6% dose, while still effective in some cases, tended to show a detrimental impact on growth parameters, suggesting a possible threshold effect. Such disparities in effects among crops and doses suggest that multiple factors affect the plant response to frass application, including the quality and origin of the frass, as well as the optimal application doses for each crop species. These factors could also alter the physical properties of the soil, as an excess of frass may cause compaction or waterlogging, thereby limiting oxygen diffusion and the availability of essential nutrients to plant roots. In addition, frass applications may modify soil microbial activity, pH and salinity, which could contribute to the inhibitory effects on plant growth observed at higher doses (Gebremikael et al. 2022). Further investigation is required, as excessive doses may negatively affect plant performance, inducing growth inhibition and/or physiological stress (Zhang et al. 2012, Liu et al. 2019, Chia et al. 2024).

The findings from the choice test showed that SPW adults exhibit variable oviposition preferences depending on the treatment and host plant species. At the beginning of the experiment, the 2% MWF treatment resulted in the highest number of laid eggs on eggplant and sweet pepper, indicating that lower percentage of applied frass may strongly attract and enhance colonization by SPW, as also found for *Bactrocera oleae* (Gmelin) on olive trees, where all formulations based on frass proved effective in attracting adults (Koufakis et al. 2025).

Nymph numbers were highest on plants receiving the 2% and 6% MWF applications, suggesting that nitrogen supplied by these treatments enhances whitefly development, in agreement with previous studies reporting increased immature stages, fecundity, survival, and developmental rate under higher nitrogen levels on various host plants (Godfrey et al. 1999, Bi et al. 2001, Hasanuzzaman et al. 2018).

The no-choice test, in which SPW was forced to infest each single plant, provided additional insights into its oviposition and developmental capacity on treated and untreated plants. As in the choice test, the 2% and 6% frass treatments resulted in the highest numbers of eggs and nymphs, indicating once again that the amount of nitrogen associated with these applications can promote whitefly population growth and support pest development (Jauset et al. 2000, Bi et al. 2001, Hasanuzzaman et al. 2018). This trend further reinforces the patterns observed in the choice test, where different frass doses had variable effects on SPW population growth, depending also on the plant species. Specifically, on eggplant, SPW consistently reached the highest densities on untreated plants, with significantly greater numbers of eggs and nymphs. This may be related to plant nutrition, as nutrient-rich plants, while generally more attractive to pests, exhibit greater inducible defense potential, allowing them to synthesize anti-insect metabolites and activate compensatory growth, following the pest attack (Li et al. 2024). Moreover, the observed effects may also be linked to the activation of systemic plant defenses by frass application, as insect frass can act as an elicitor of systemic resistance. Indeed, Zunzunegui (2025) reported that field application of frass produced by the larvae of *Hermetia illucens* L. activated systemic defenses, reducing infestation by SPW and other pests. In addition, chitin-derived products, including chitosan, demonstrate to be toxic to plant pests, induce plant defenses, and stimulate beneficial microbial activity, with effective control reported for sap-sucking insects (Sharp 2013).

The species-specific response observed in our study is supported by multiple research reporting contrasting effects of frass on different plant species. While frass can induce plant defense mechanisms, these responses may not be effective against all pests, depending on the specific plant–pest system, and in some cases may even suppress other defense mechanisms (Ray et al. 2015, Liu et al. 2019, Wantulla et al. 2023).

The valorization of MWF as a soil improver and potential bioprotectant fits well within circular economy models by transforming insect farming by-products into valuable agricultural inputs, thereby reducing waste and enhancing resource efficiency (Van Huis and Oonincx 2017, Poveda 2021a). Its dual functionality could contribute to more sustainable crop production systems by decreasing dependence on synthetic agrochemicals and improving soil health.

However, the variable pest management effects observed indicate that frass cannot yet be reliably recommended as a standalone biocontrol agent against *B. tabaci* MED, as well as a standard mitigation tool for all crops.

Based on the results obtained in this study, MWF can be considered a valuable organic amendment that enhances plant growth to tomato, sweet pepper, and eggplant, especially when used at the lowest application rates (ie 2%). On the other hand, MWF effects on oviposition and density of immature stages of the whitefly varied among plant species. Overall, these findings highlight the potential of *T. molitor* frass as a sustainable input to improve crop performance and modulate *B. tabaci* dynamics, with outcomes depending on both dose and host plant.

Further studies should evaluate plant species- or cultivar-specific responses to frass application, elucidate its biochemical and microbiological mechanisms, and assess its effects on other pests and natural enemies. Microbiological characterization could help to identify compounds responsible for fertilizing effects and for modulating pest attraction and development, thereby supporting reduced fertilizer use, enhanced soil fertility, and circular economy aims in a sustainable agriculture vision.

## Supplementary Material

toag057_Supplementary_Data
