# Supplementary material for: Potential use of mealworm frass as soil amendment and pest management tool in circular economy
Source: J Econ Entomol. 2026 Mar 19;119(3):2072–86. doi: 10.1093/jee/toag057 (PMC13268529; doi:10.1093/jee/toag057)
Supplement: toag057_Supplementary_Data [file toag057_supplementary_data.docx]

Supplementary Materials: Potential use of mealworm frass as soil amendment and pest management tool in circular economy

Luca Maria Girgenti, Marco Di Domenico*, Rania Rachdi, Alessia Farina, Giuseppe Eros Massimino Cocuzza, Emanuele La Bella, Ferdinando Fragalà, Andrea Baglieri, Carmelo Rapisarda, Pompeo Suma.

**Figure S1. Ongoing vegetative growth observed in tomato treatments.** The graphs illustrate the variation in mean plant height (ht) of tomato (*Solanum lycopersicum* L. cv. ‘Creativo’) across different treatments from Day 0 to Day 49 (DAT).

| 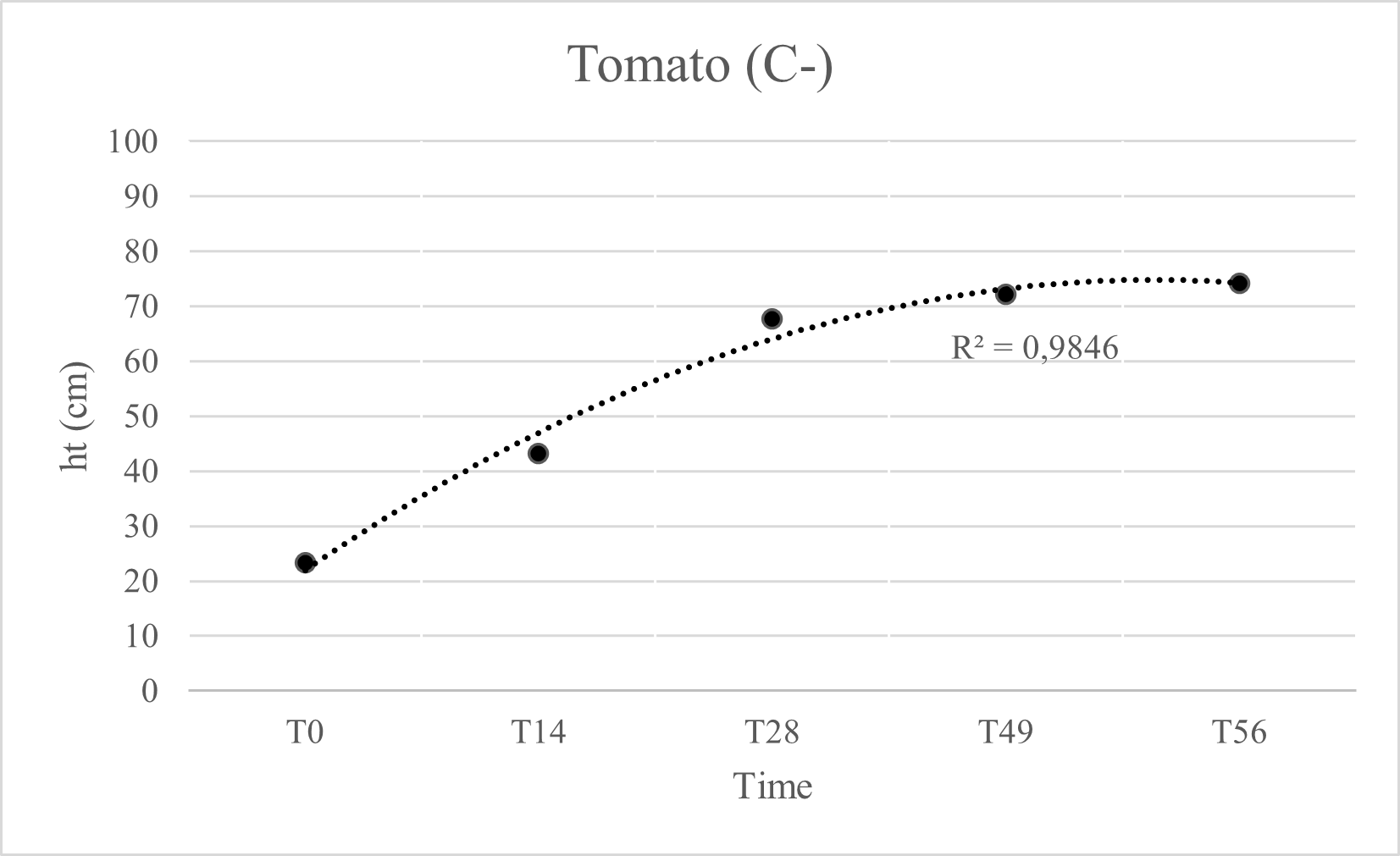 | 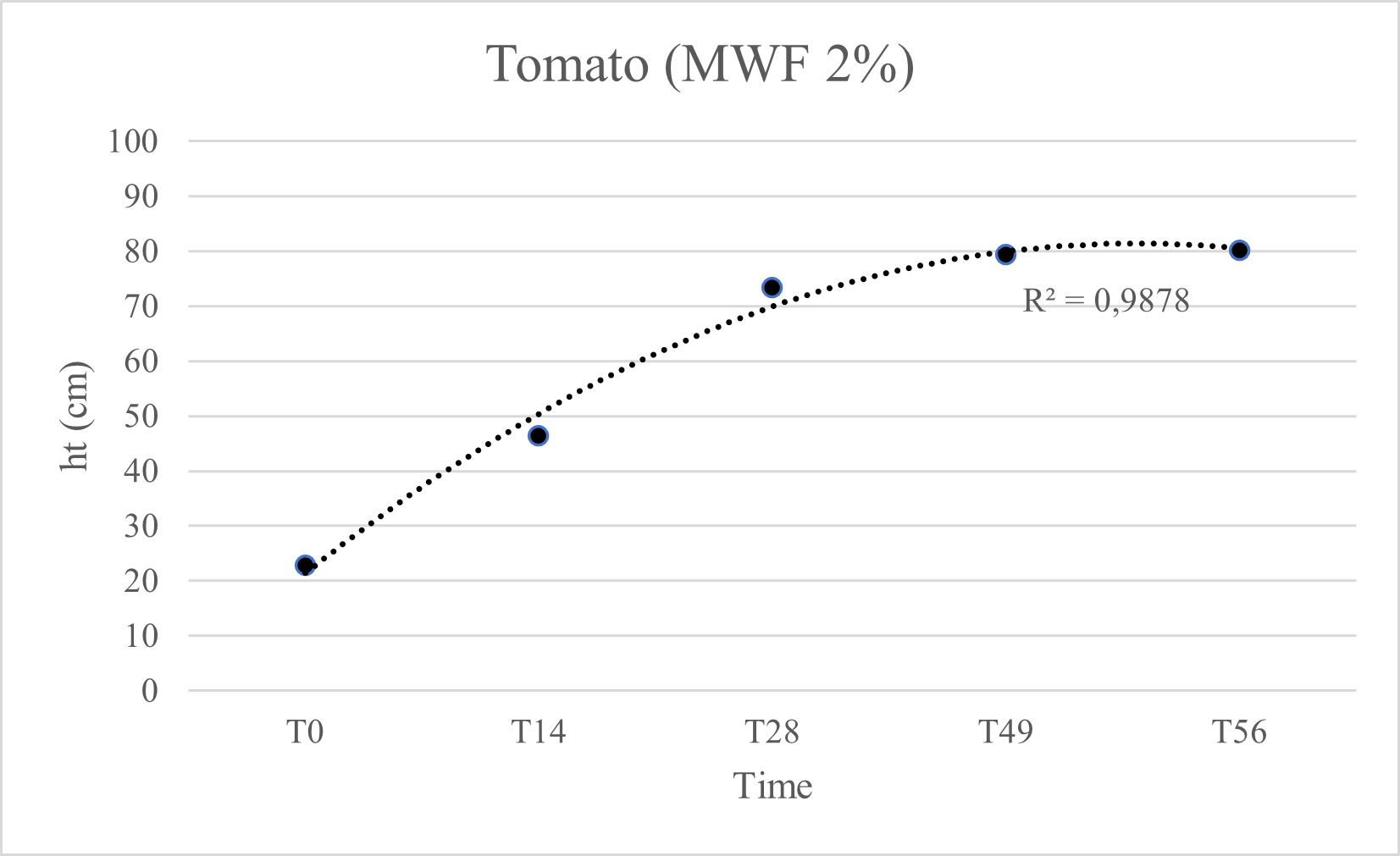 |
| --- | --- |
| 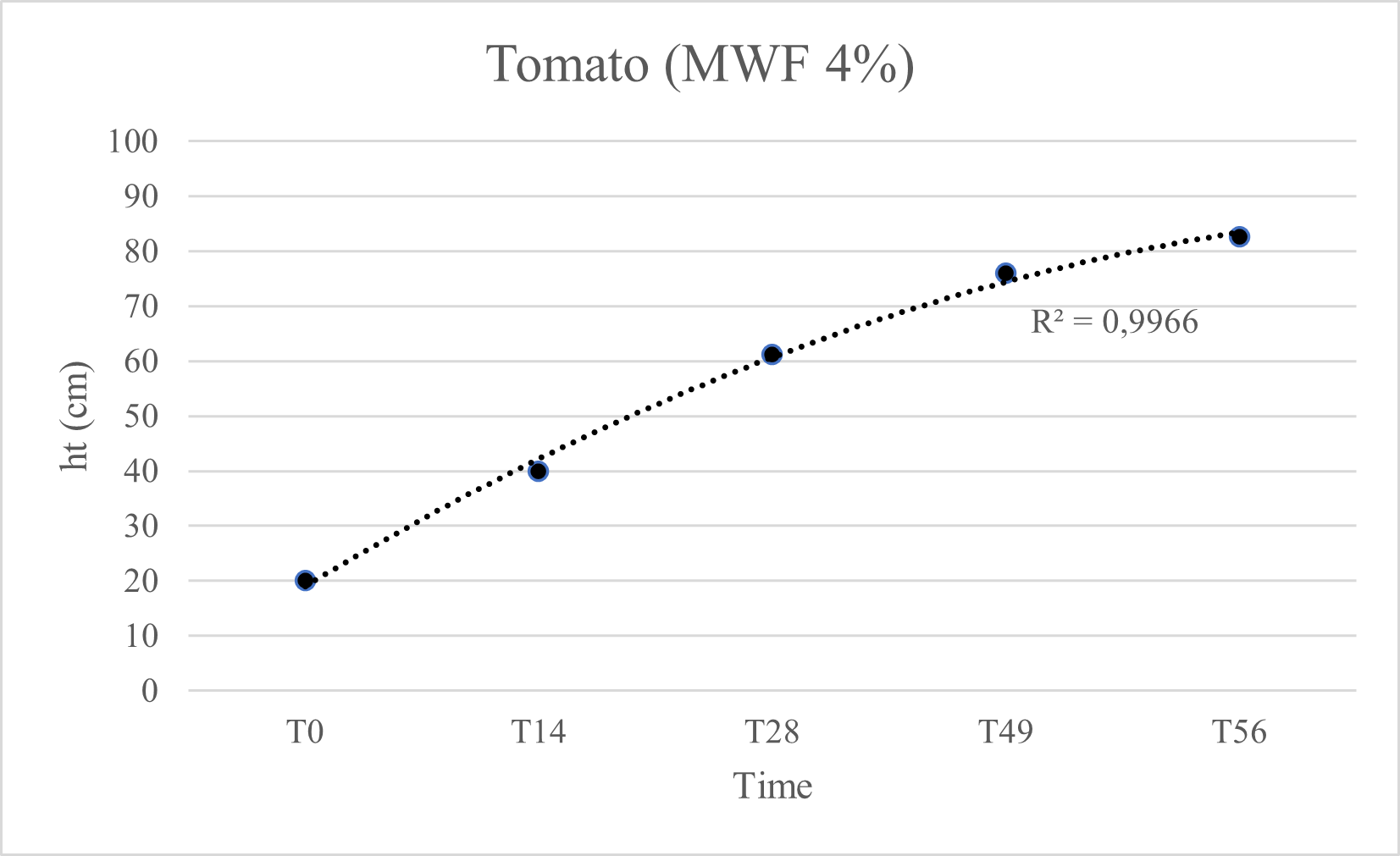 | 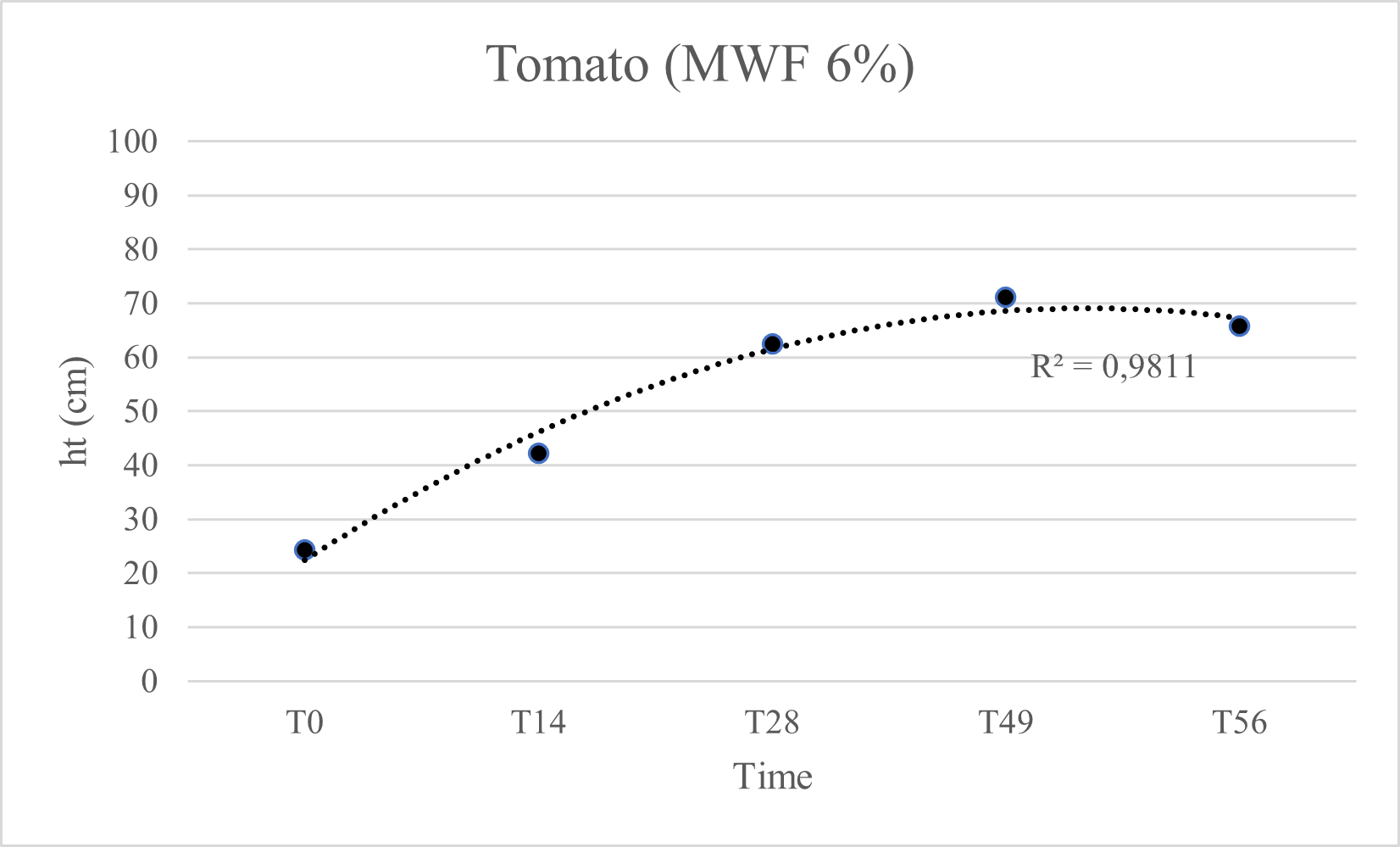 |
| 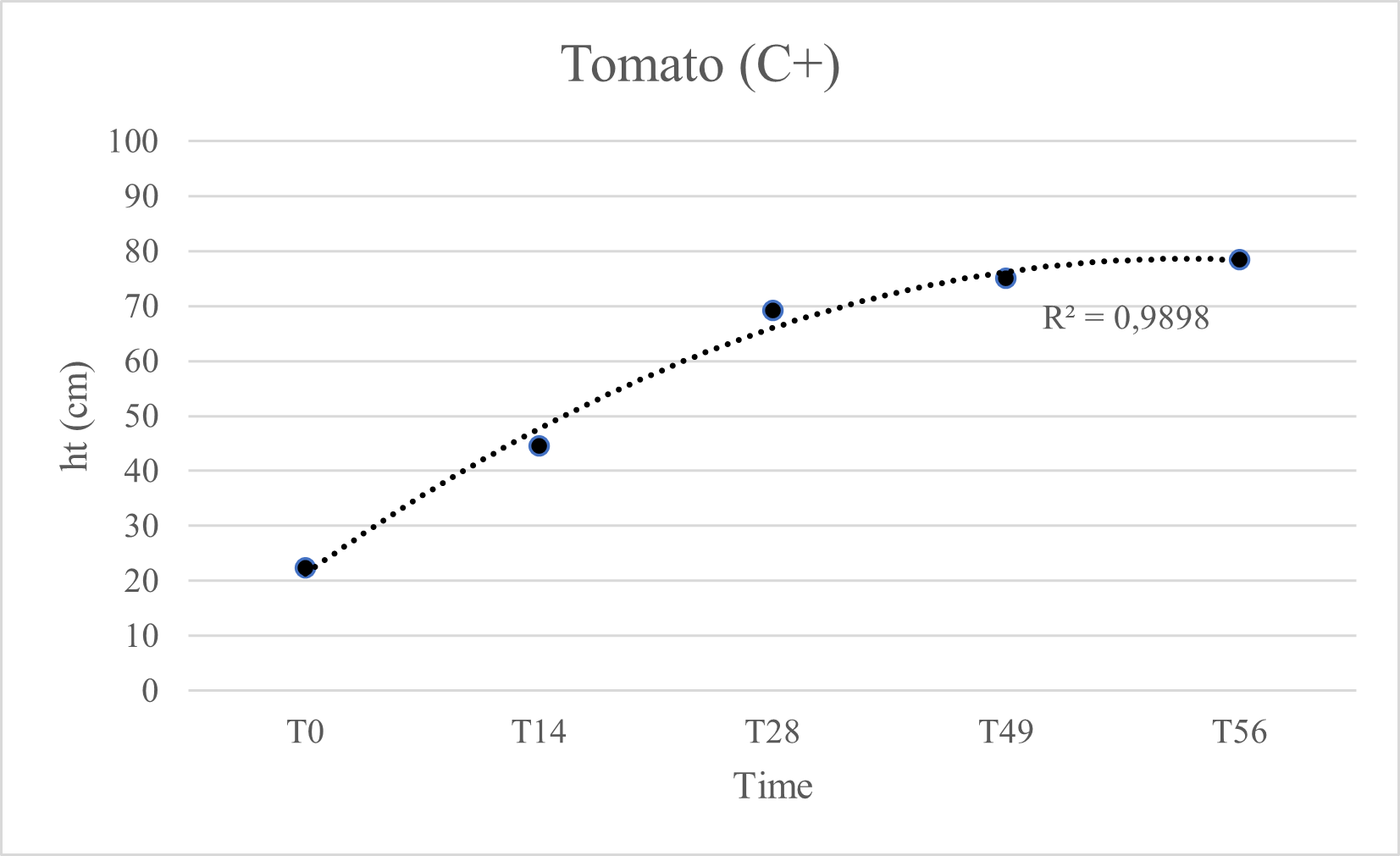 | |

**Figure S2. Ongoing vegetative growth observed in sweet pepper treatments.** The graphs illustrate the variation in mean plant height (ht) of sweet pepper (*Capsicum annuum* L. cv. ‘Altea’) across different treatments from Day 0 to Day 49 (DAT).

| 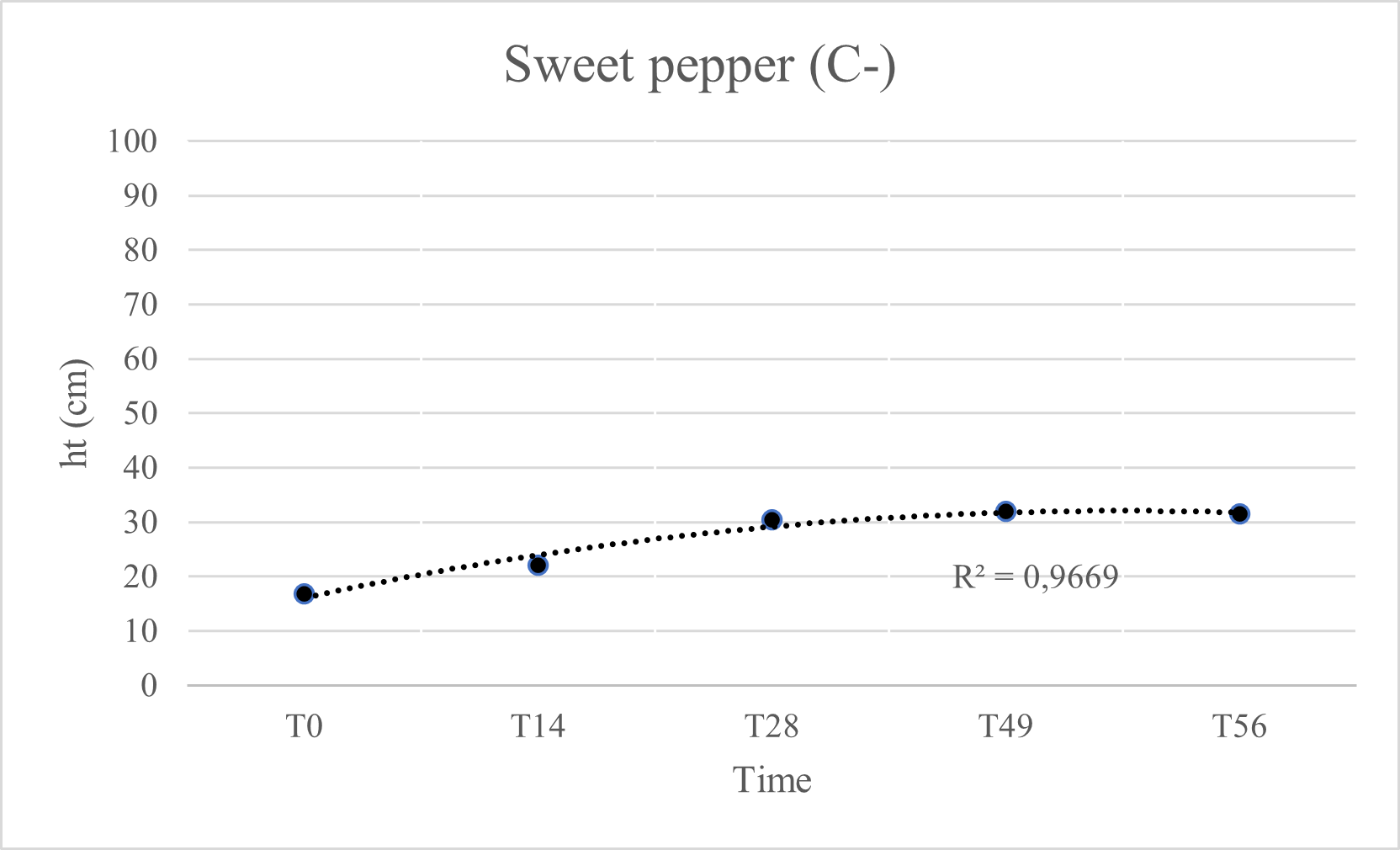 | 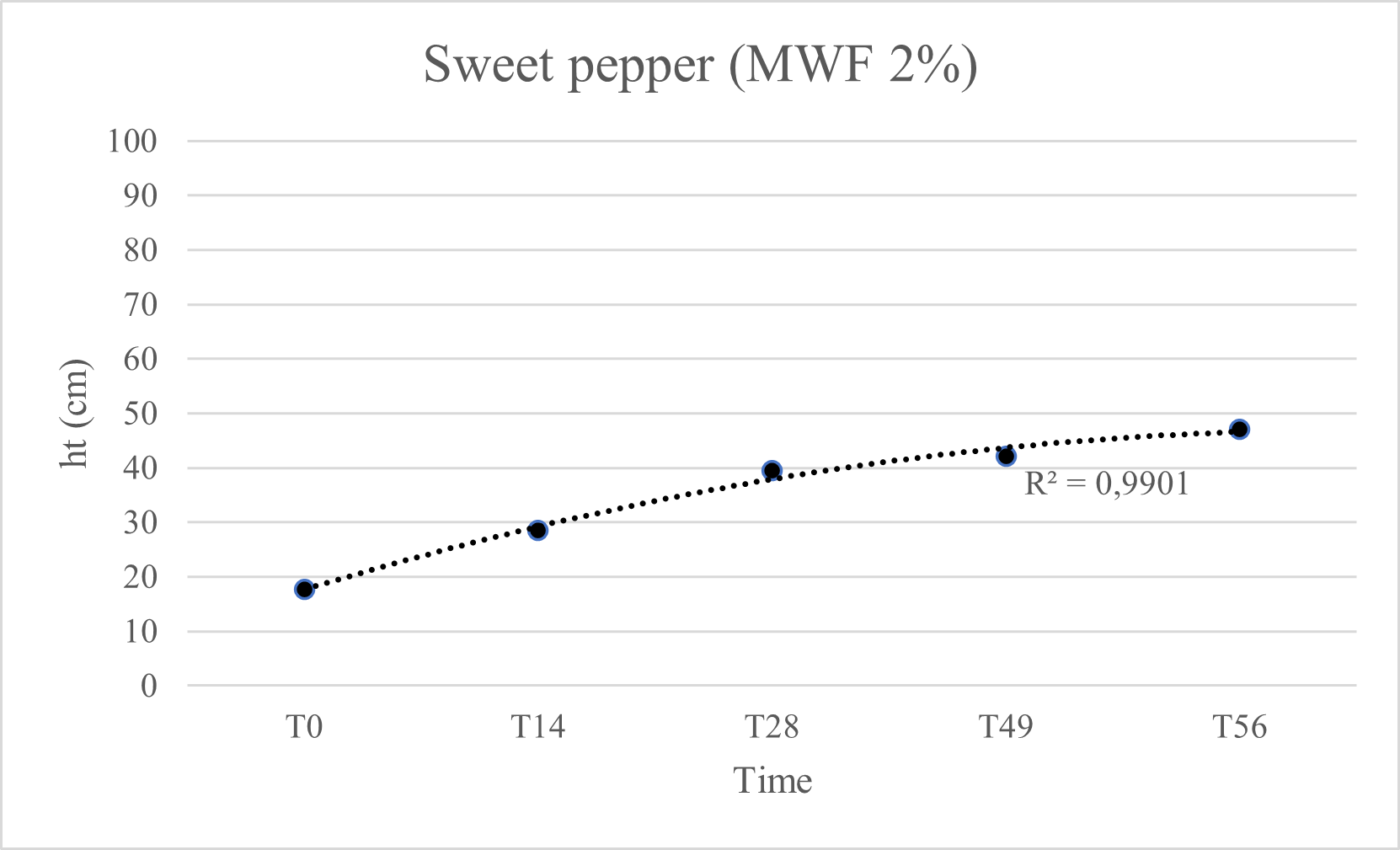 |
| --- | --- |
| 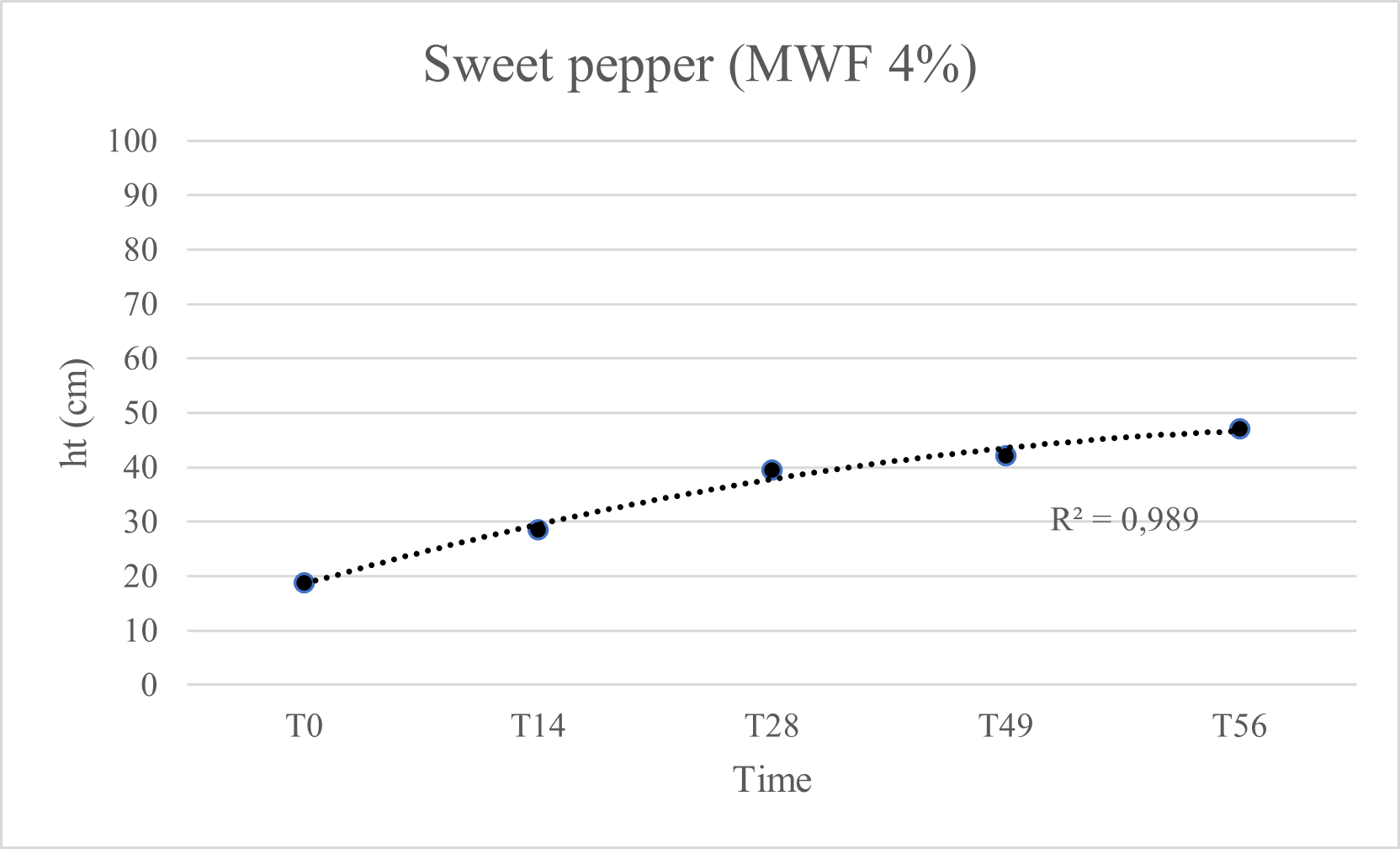 | 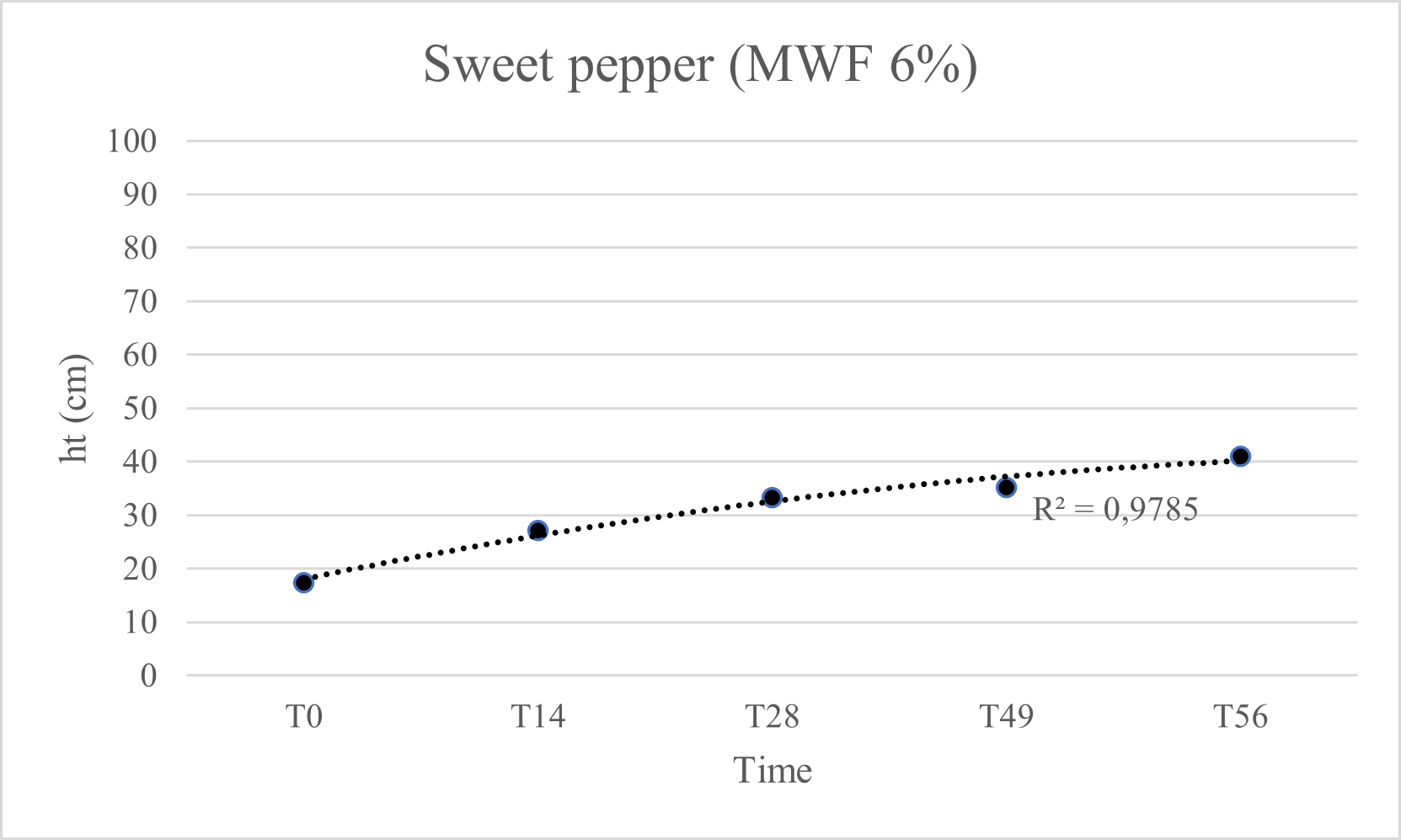 |
| 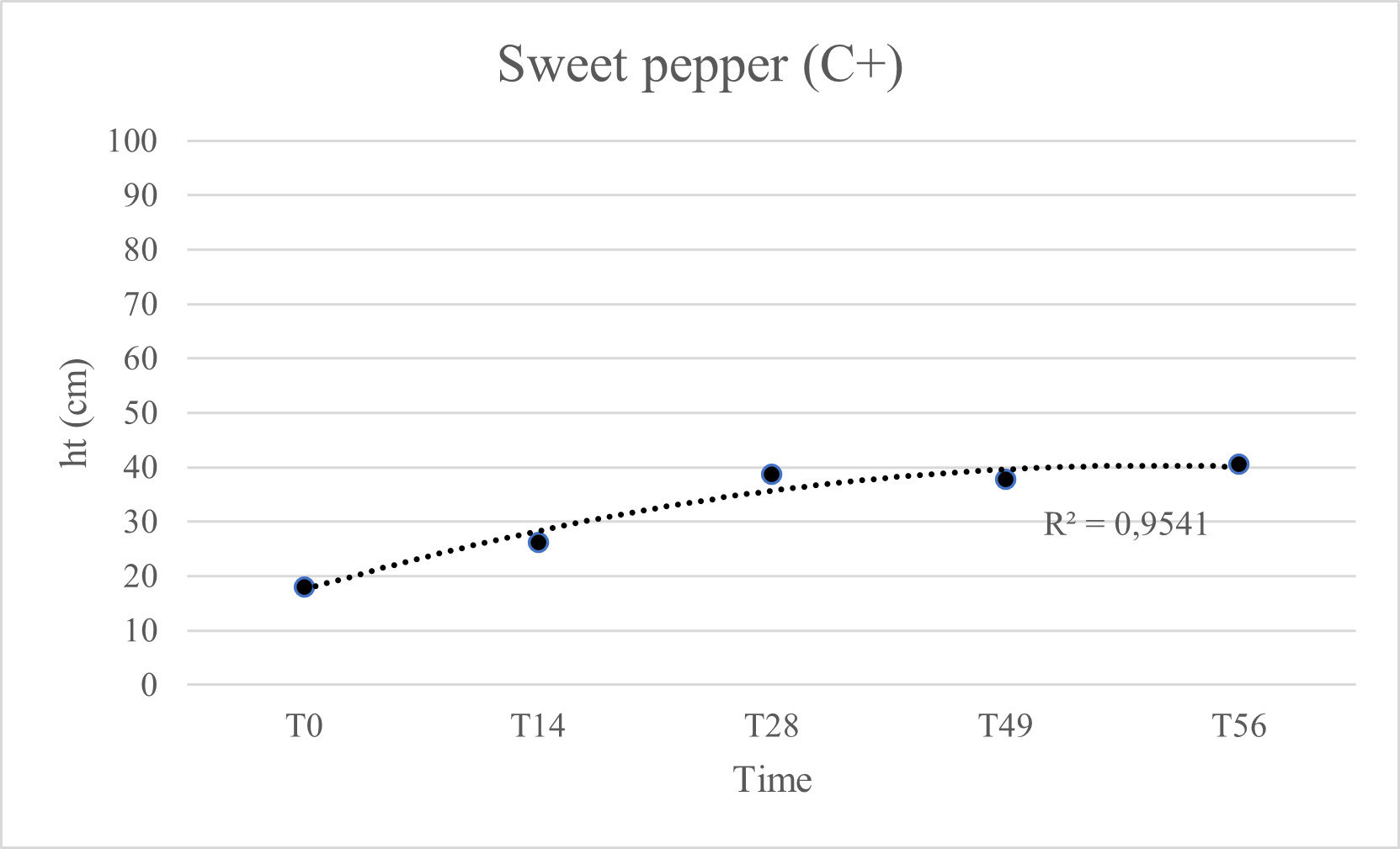 | |

**Figures S3. Ongoing vegetative growth observed in eggplant treatments.** The graphs illustrate the mean variation in mean plant height (ht) of eggplant (*Solanum melongena* L. cv. ‘Velia F1’) across different treatments from Day 0 to Day 49 (DAT) period from Day 0 to Day 49 (DAT).

| 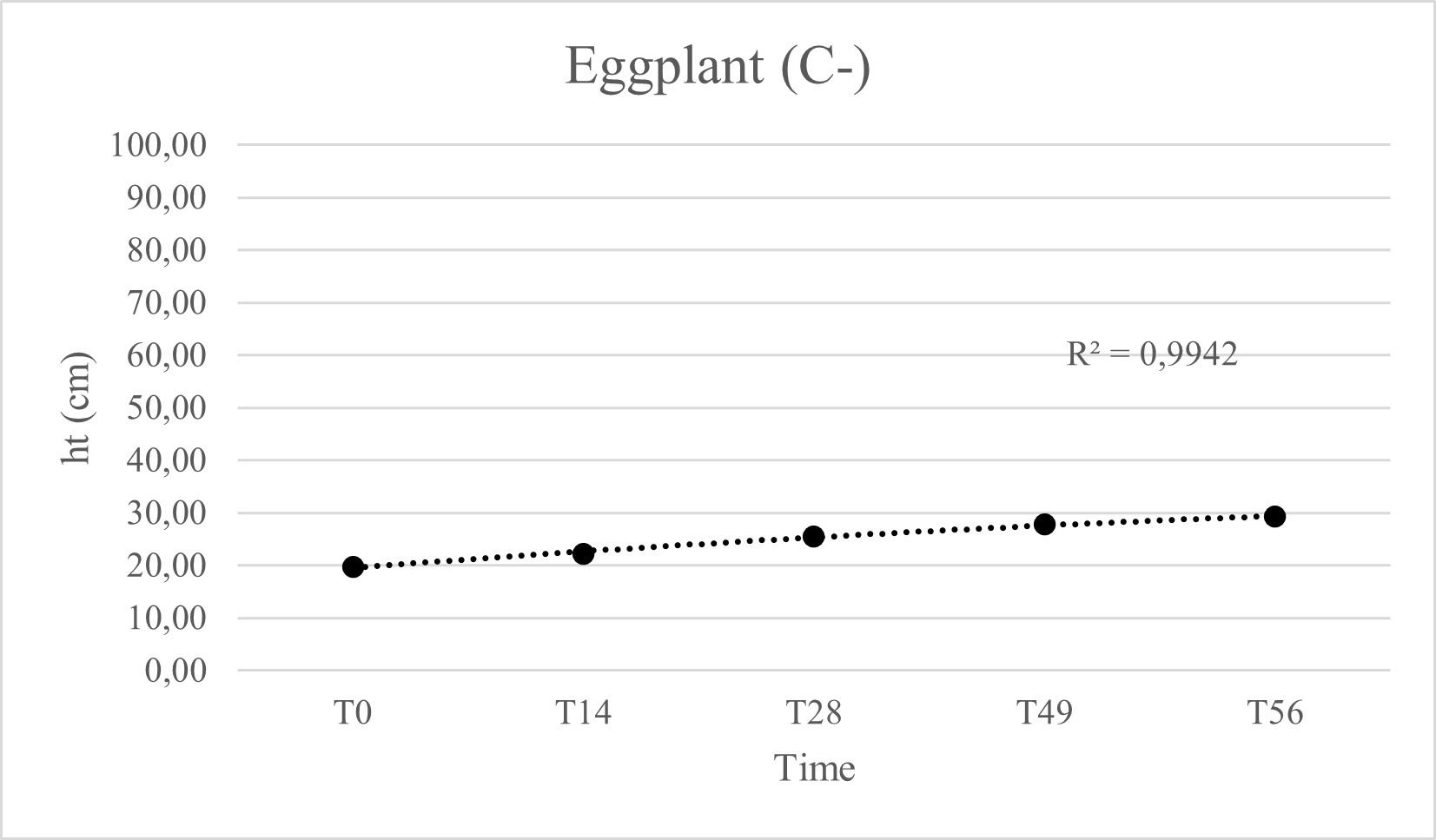 | 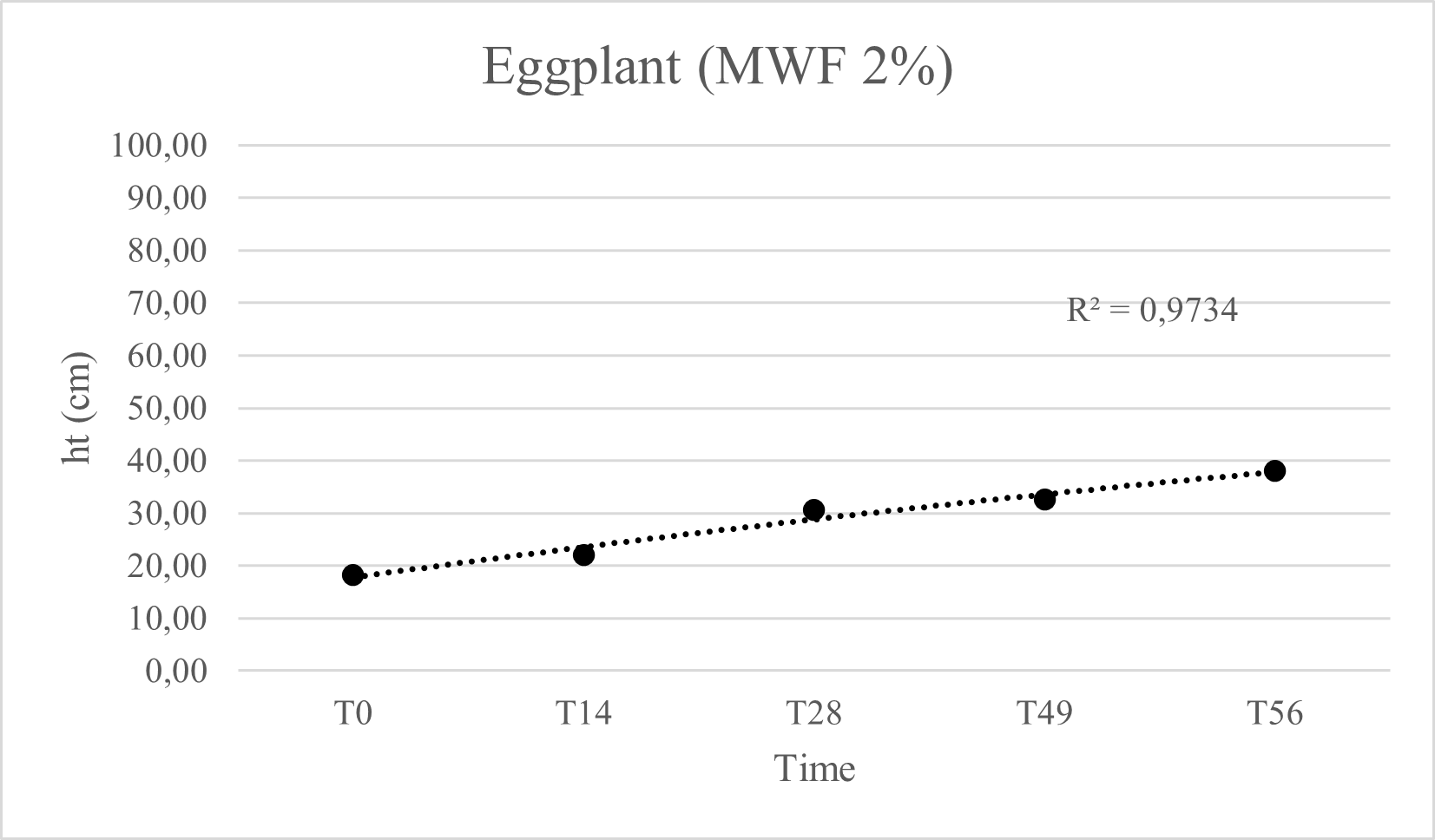 |
| --- | --- |
| 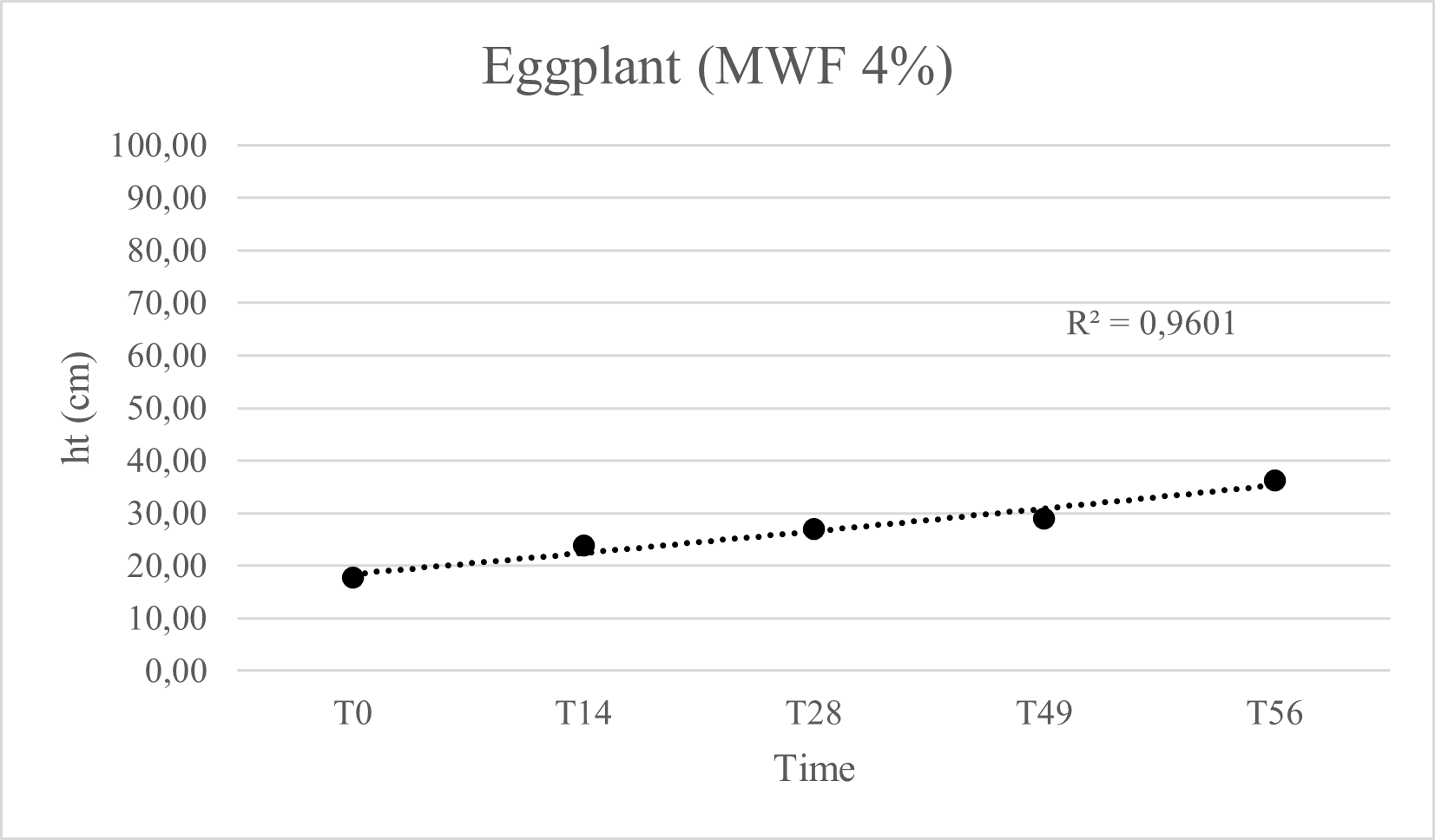 | 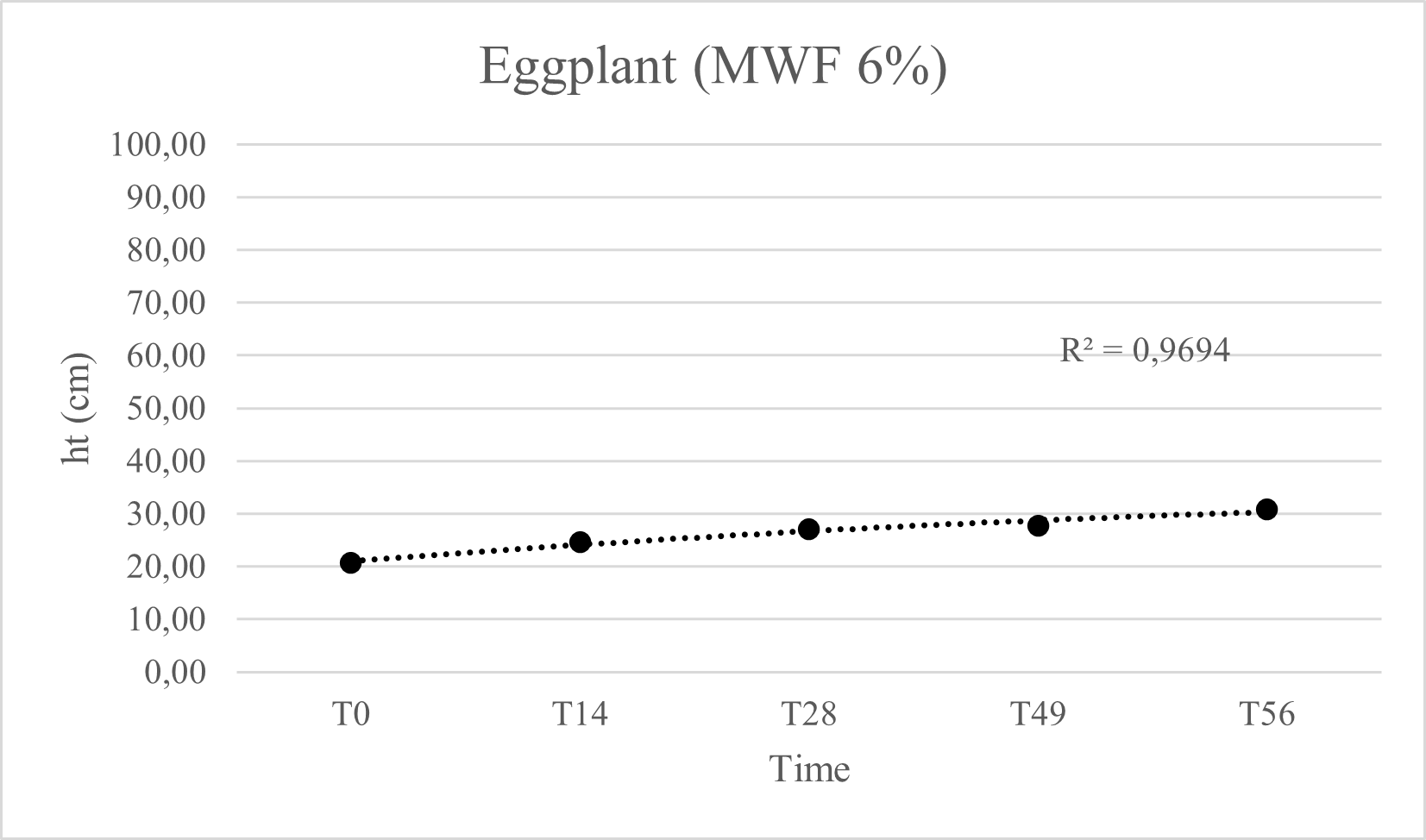 |
| 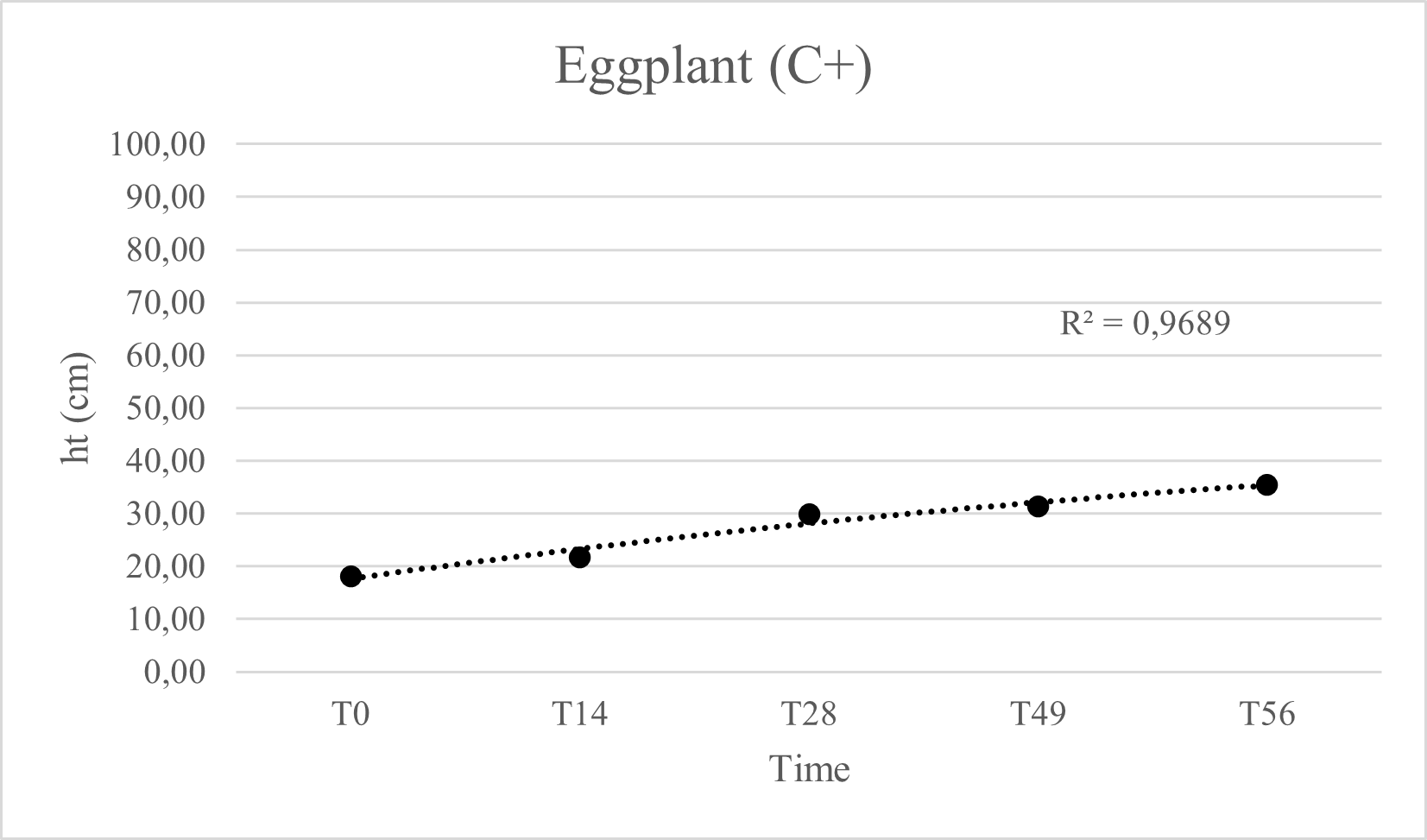 | |

**Figure S4. Gradual leaves development in tomato treatments.** The graphs illustrate the mean changes in number of leaves (ln) of tomato (*Solanum lycopersicum* L. cv. ‘Creativo’) under different treatments from Day 0 to Day 49 (DAT), highlighting the progressive leaf development throughout the experimental period.

| 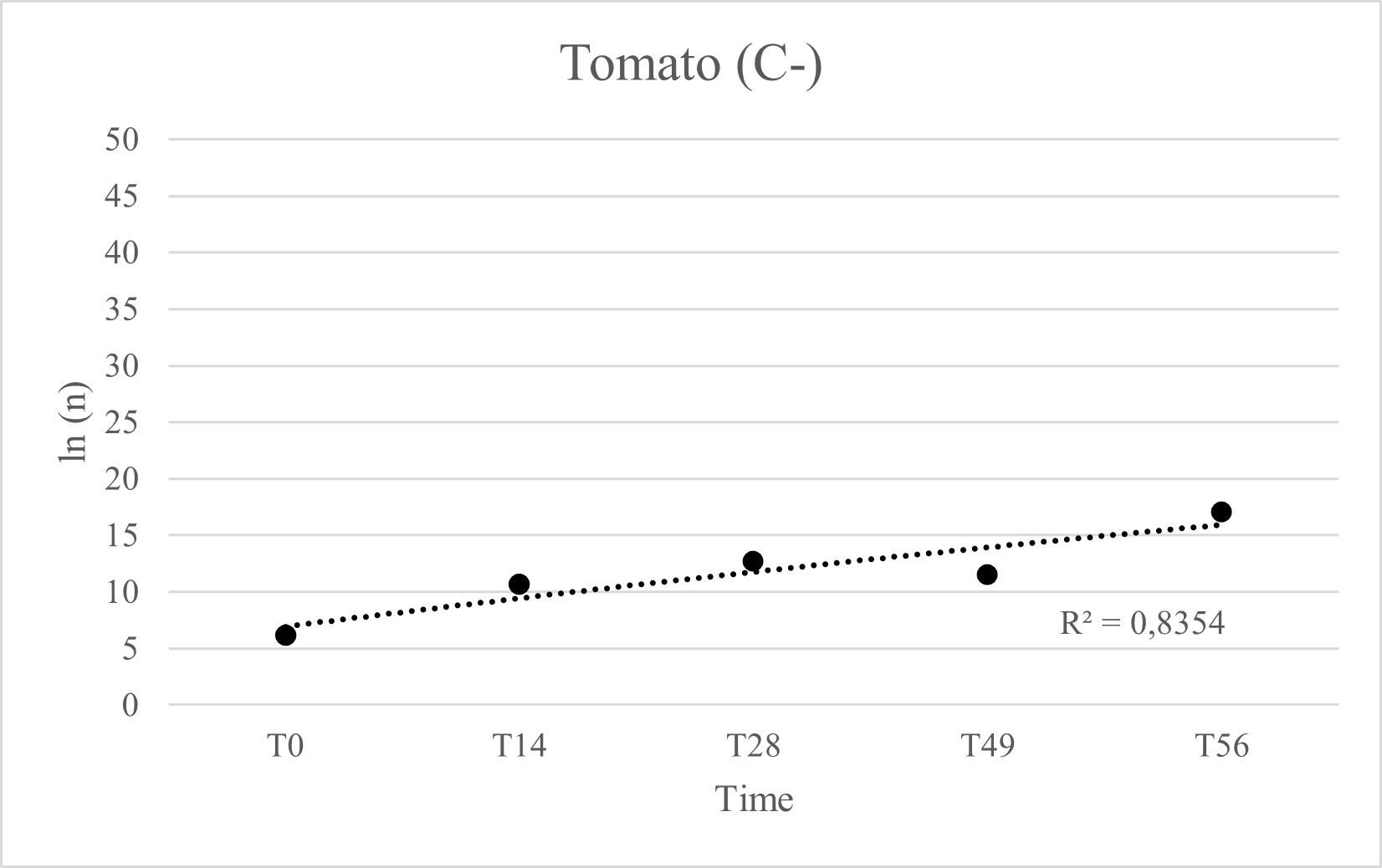 | 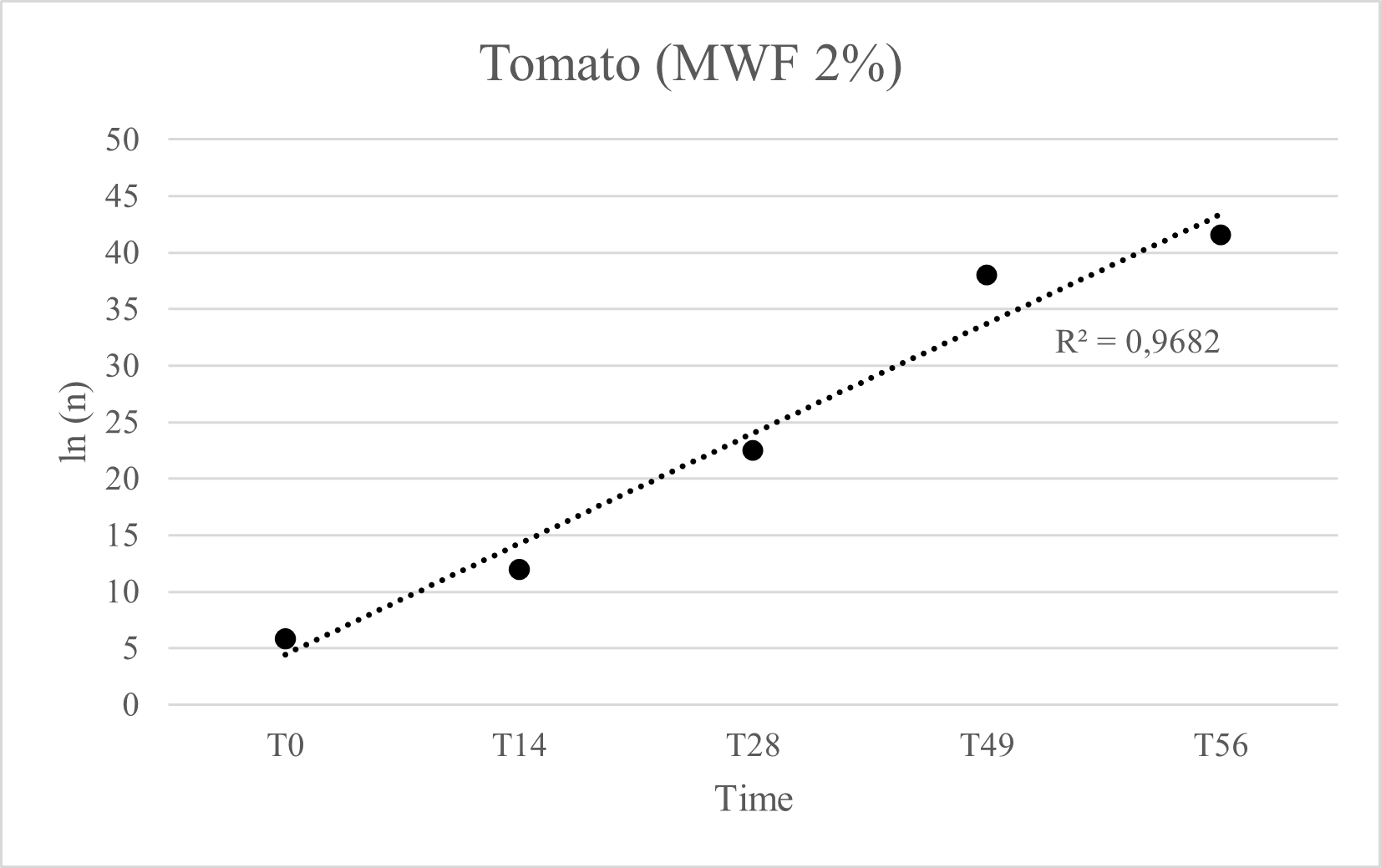 |
| --- | --- |
| 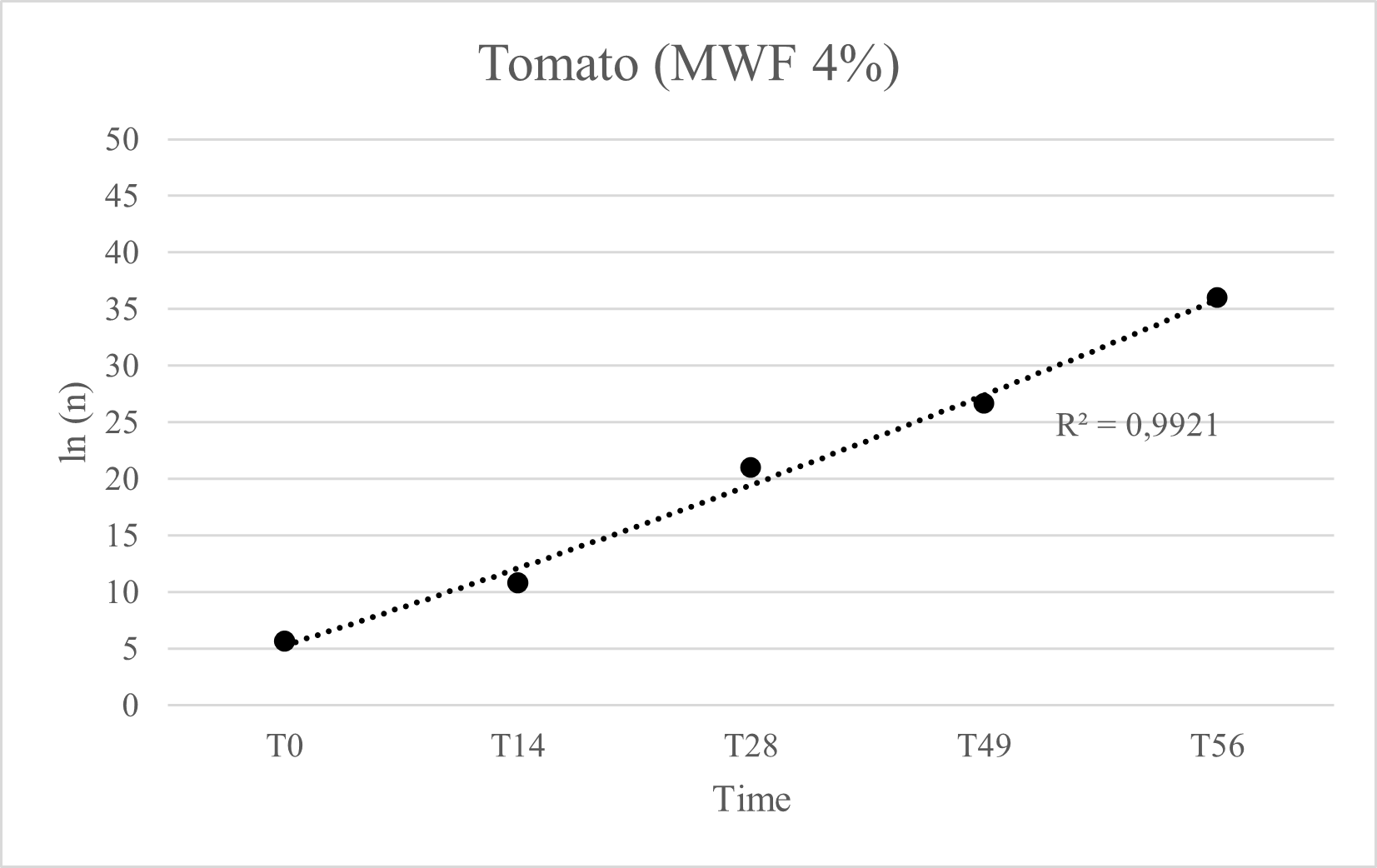 | 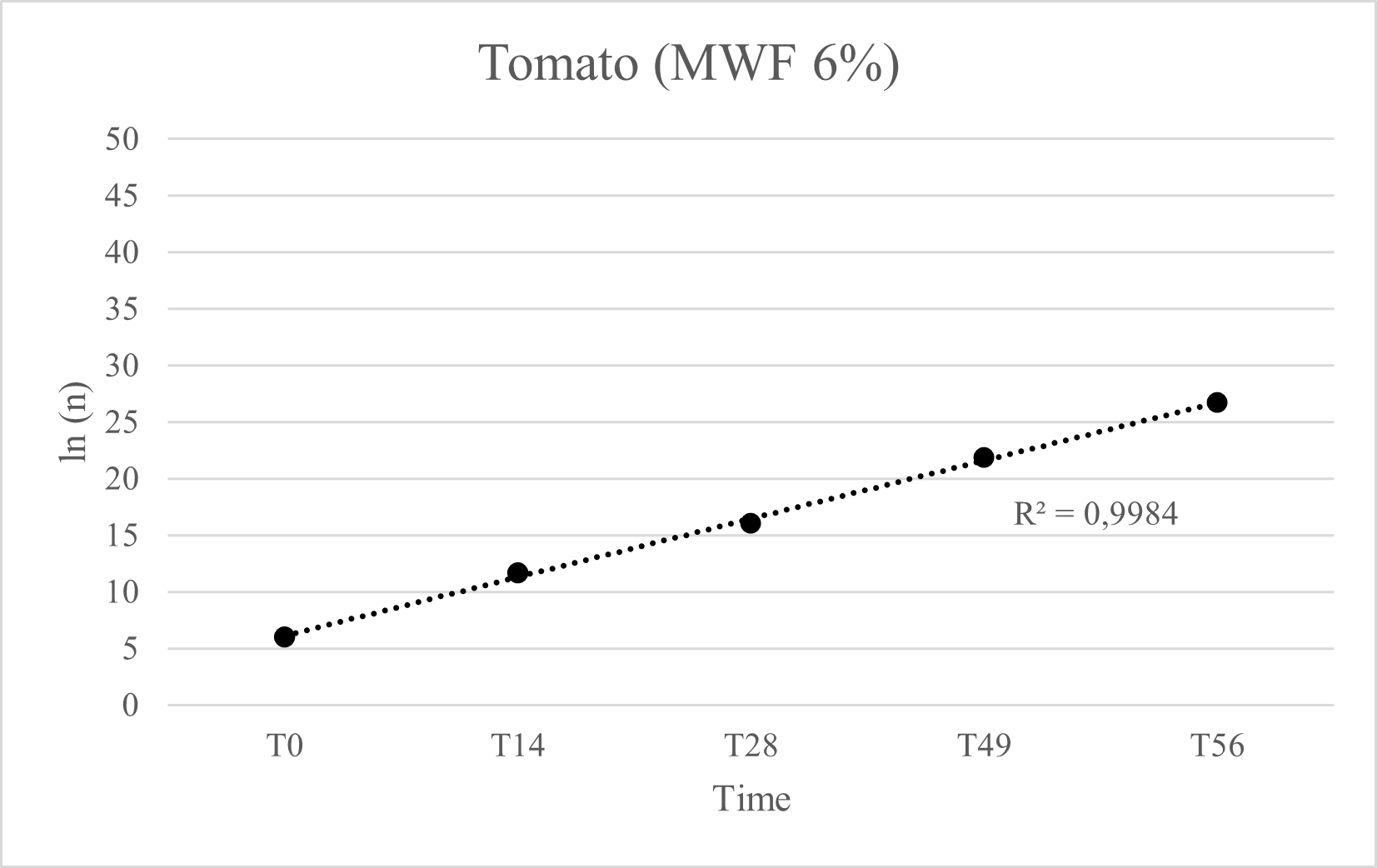 |
| 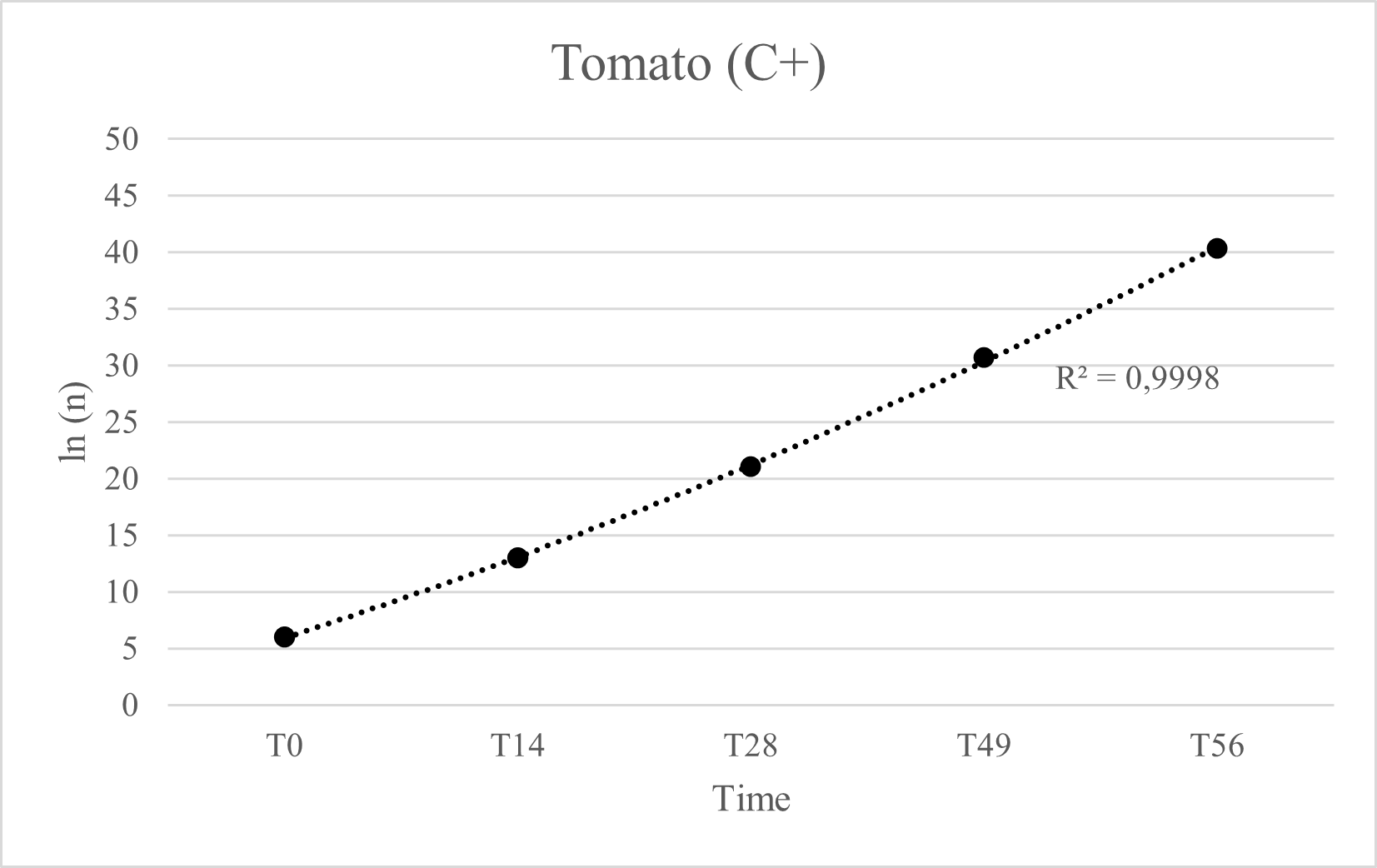 | |

**Figure S5. Gradual leaves development in sweet pepper treatments:** The graphs illustrate the mean changes in number of leaves (ln) of sweet pepper (*Capsicum annuum* L. cv. ‘Altea’) under different treatments from Day 0 to Day 49 (DAT), highlighting the progressive leaf development throughout the experimental period.

| 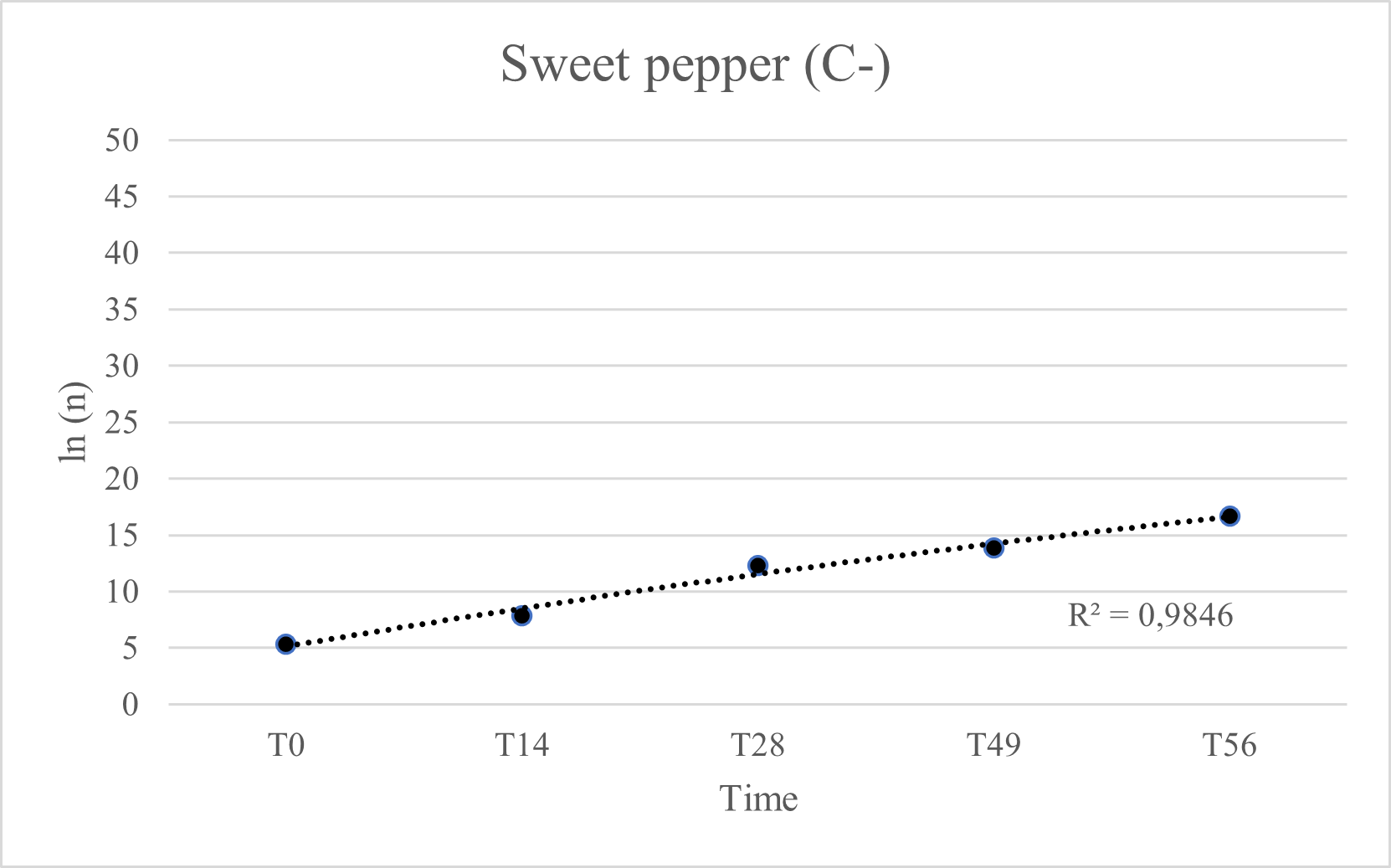 | 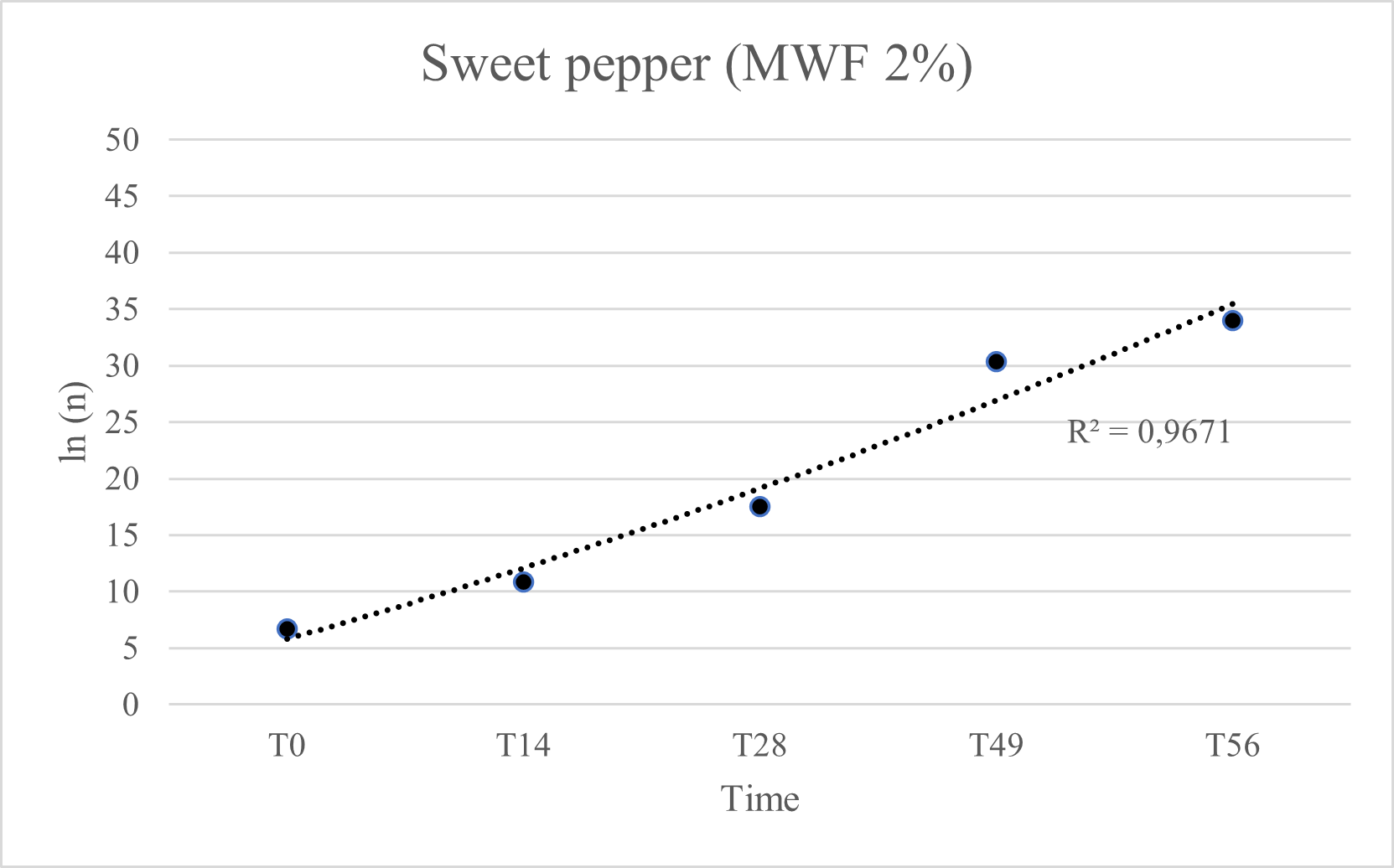 |
| --- | --- |
| 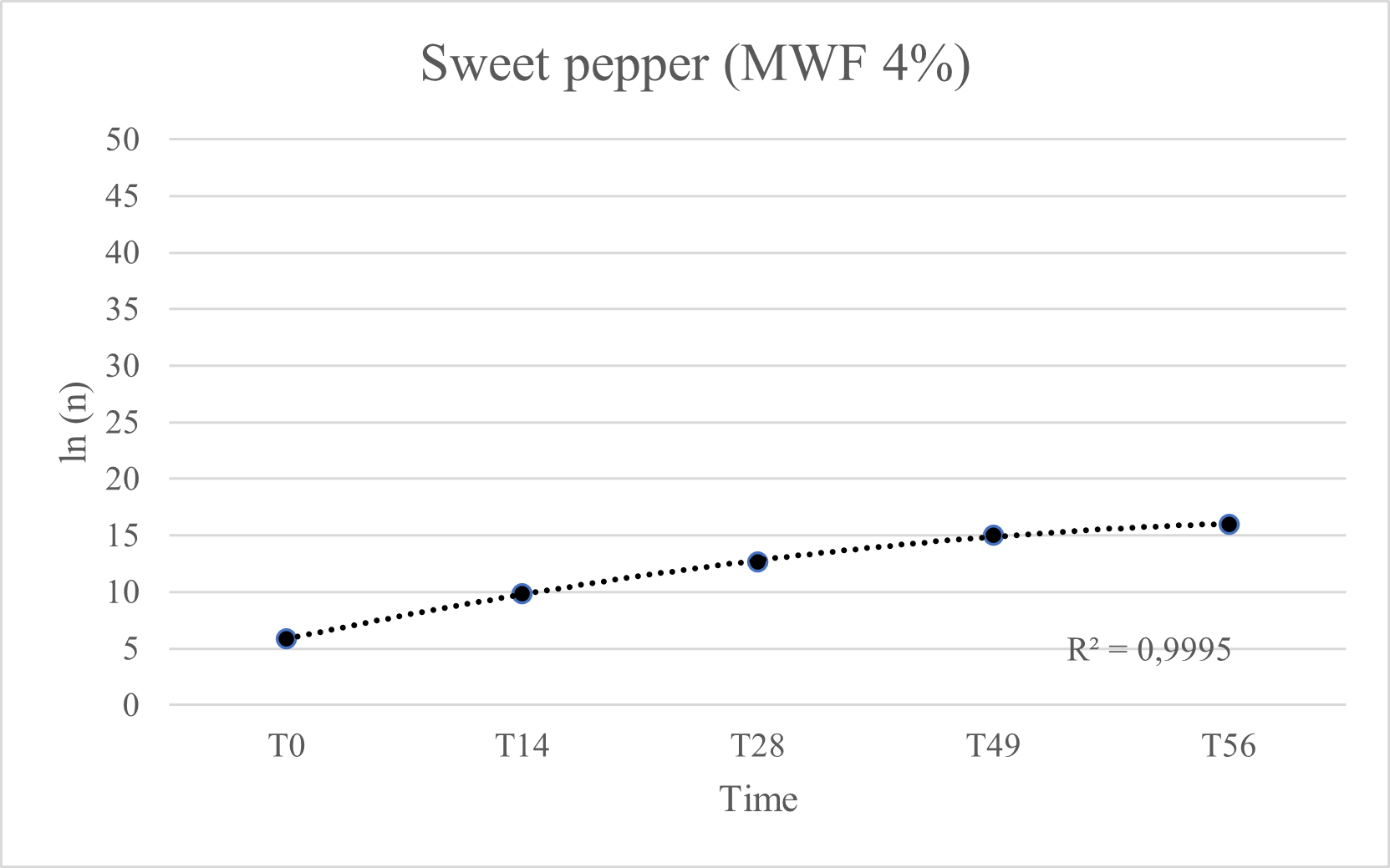 | 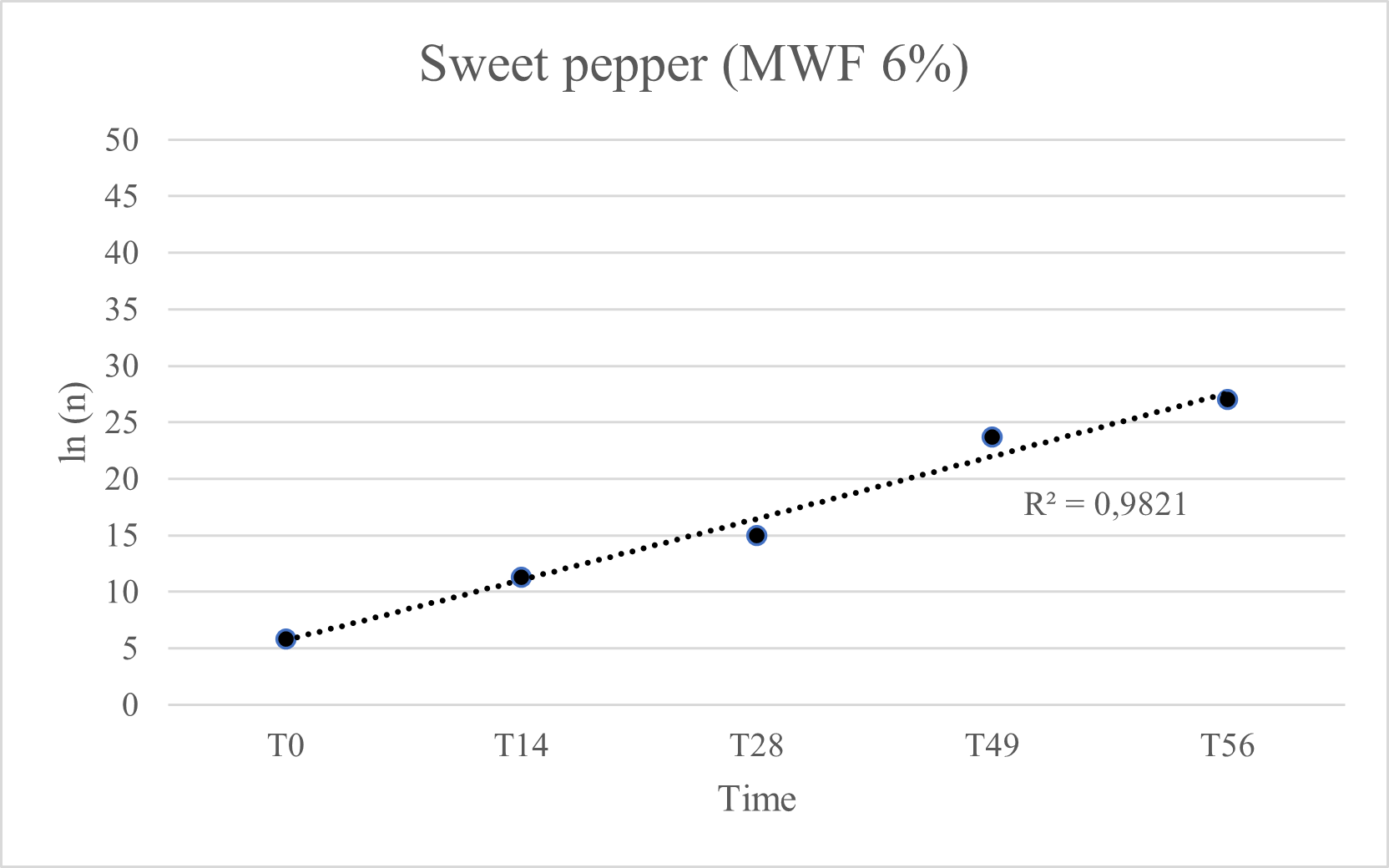 |
| 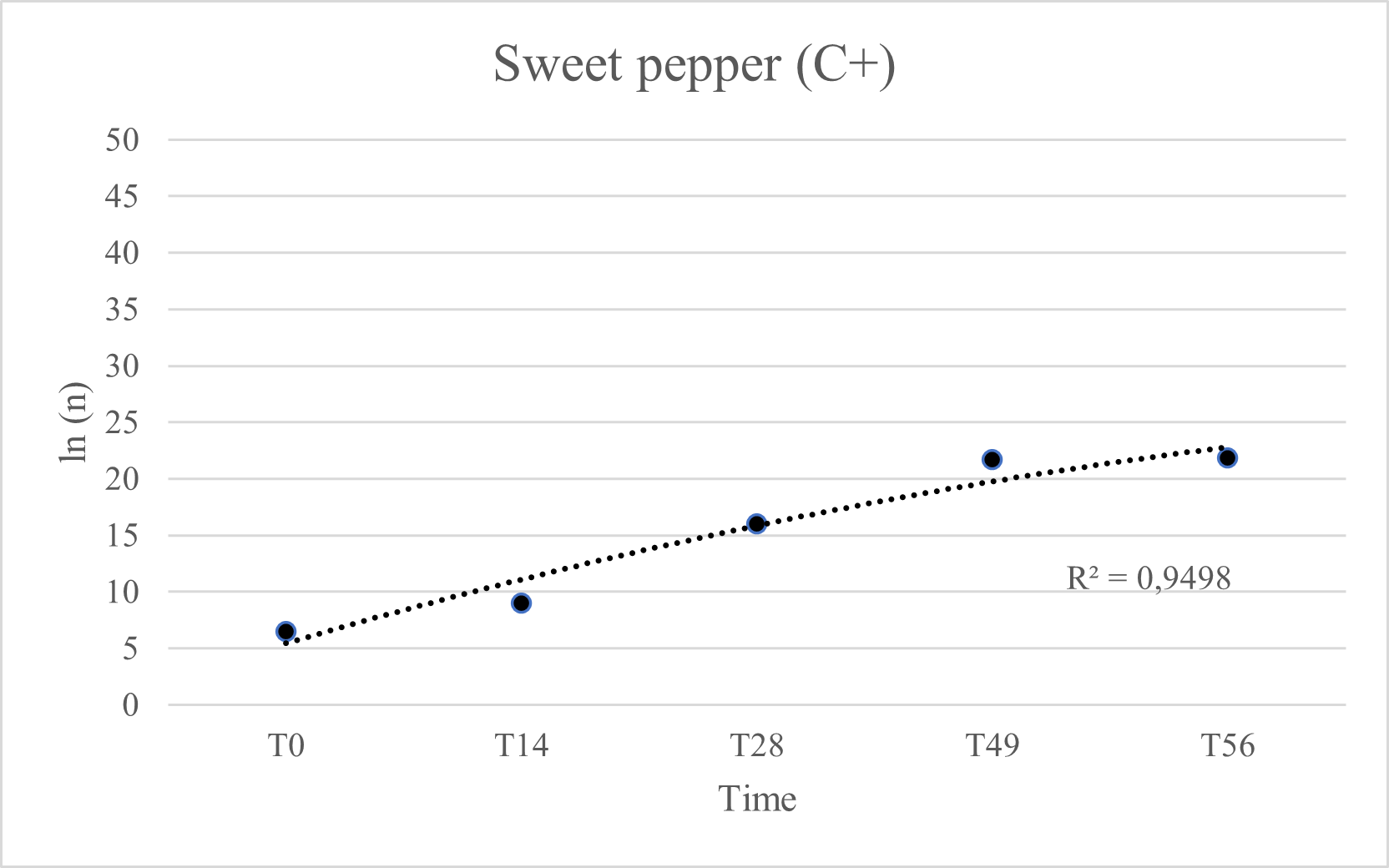 | |

**Figure S6. Gradual leaves development in eggplant treatments.** The graphs illustrate the mean changes in number of leaves (ln) of sweet pepper (*Solanum melongena* L. cv. ‘Velia F1’) under different treatments from Day 0 to Day 49 (DAT), highlighting the progressive leaf development throughout the experimental period.

| 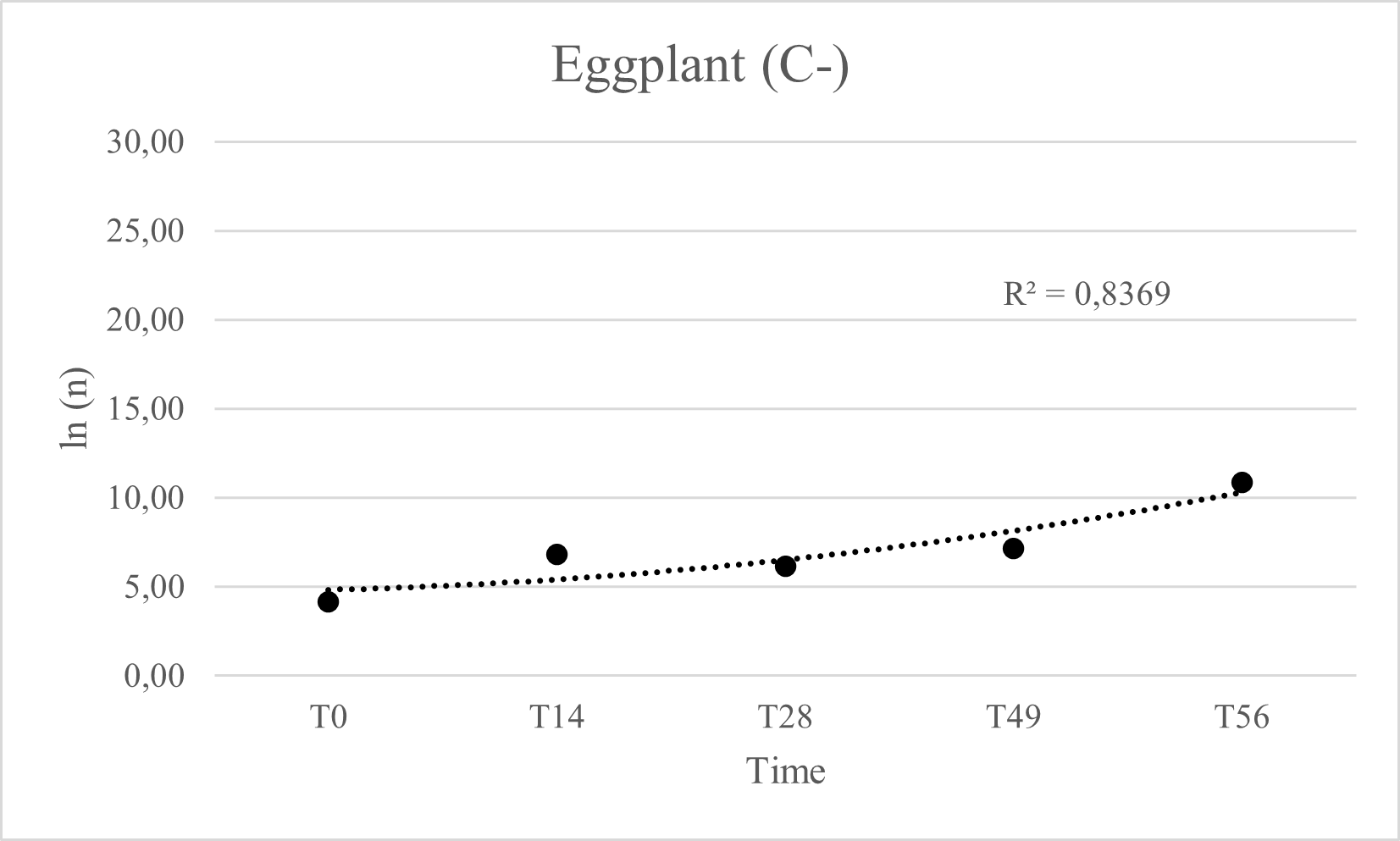 | 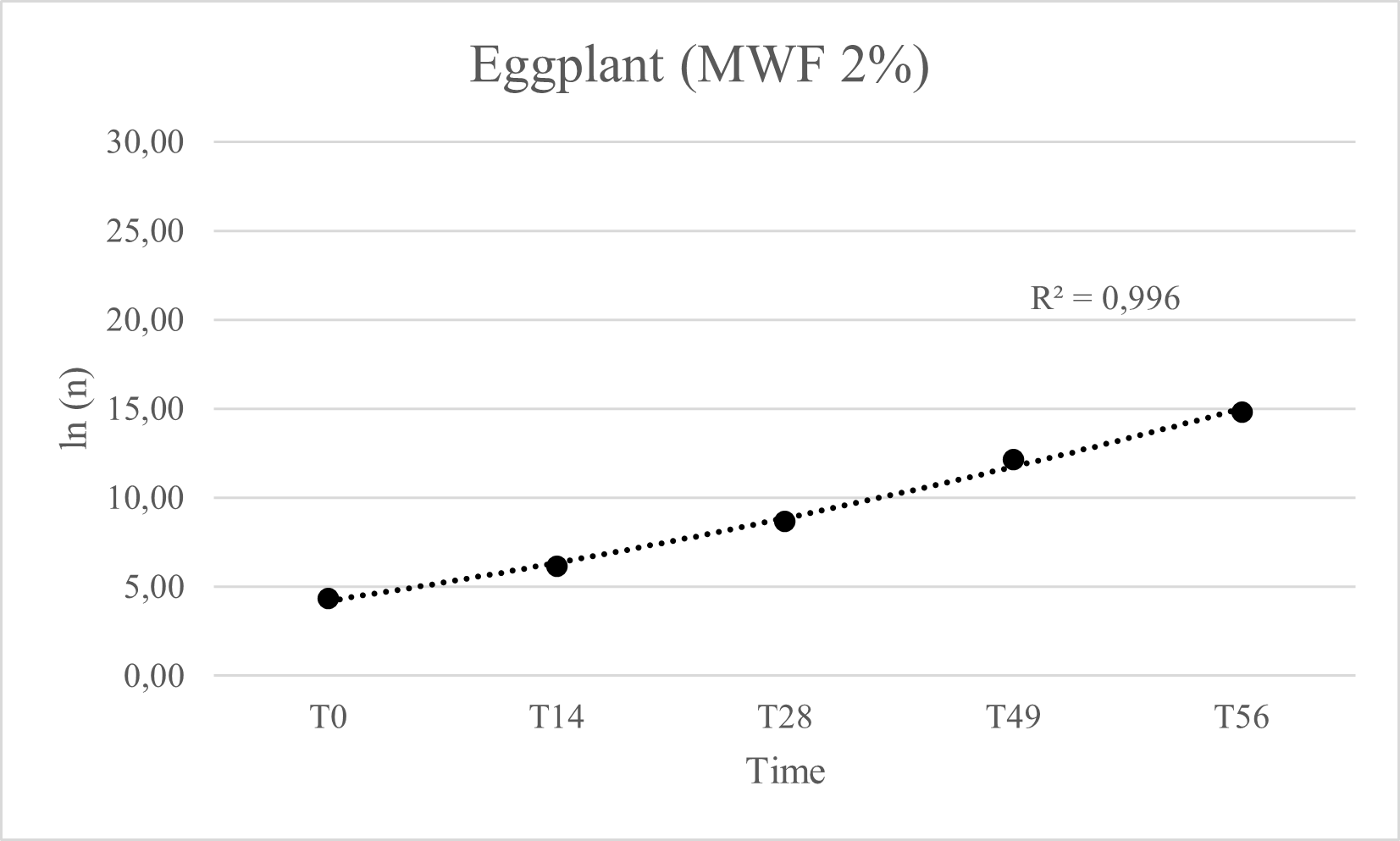 |
| --- | --- |
| 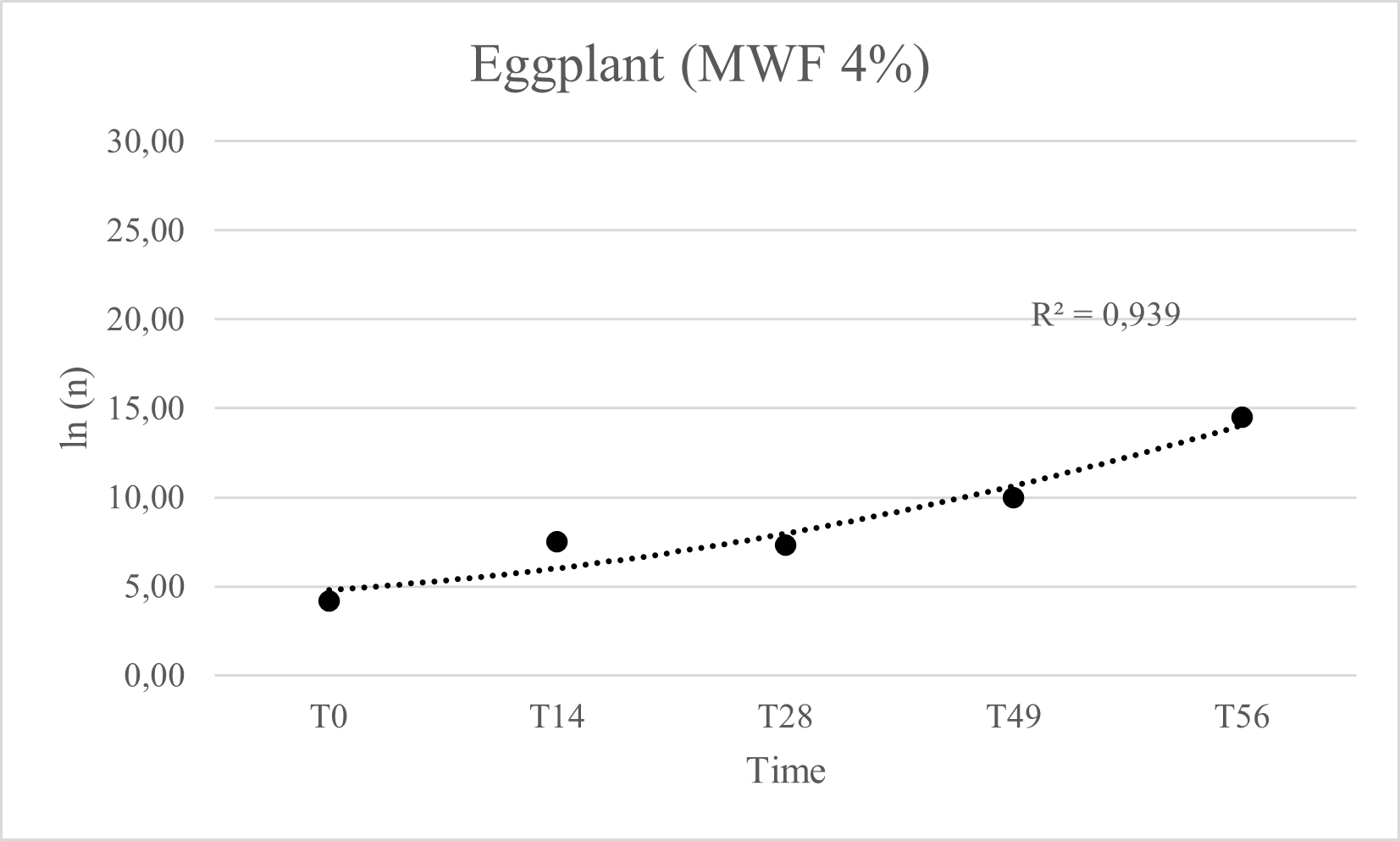 | 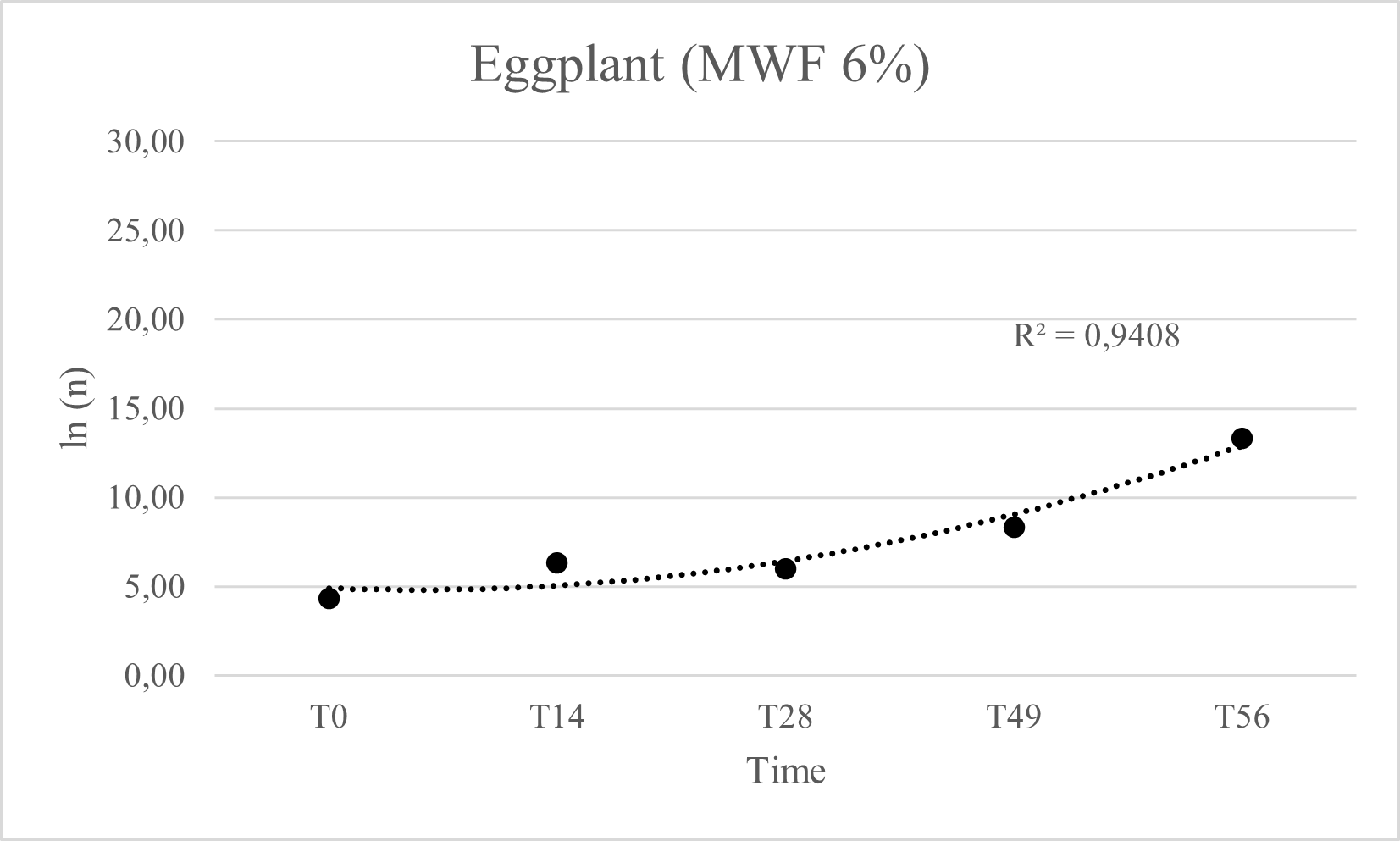 |
| 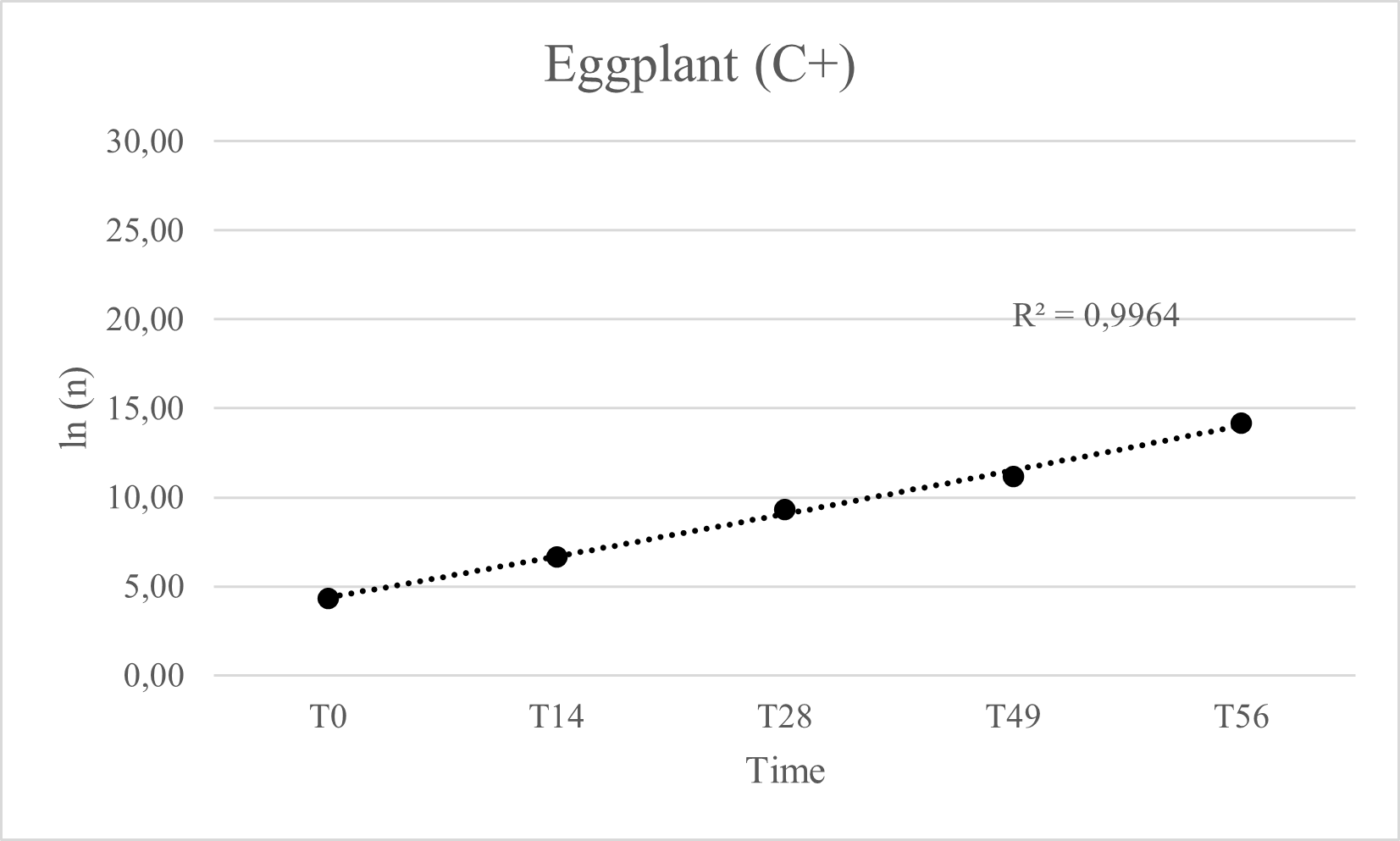 | |

**Figure S7. Dynamic changes in indirect chlorophyll content observed in tomato treatments**. The graphs illustrate the mean variation in indirect chlorophyll content (ICC, measured in SPAD units) of tomato (*Solanum lycopersicum* L. cv. ‘Creativo’) across different treatments during the experimental period from Day 14 to Day 49 (DAT).

| 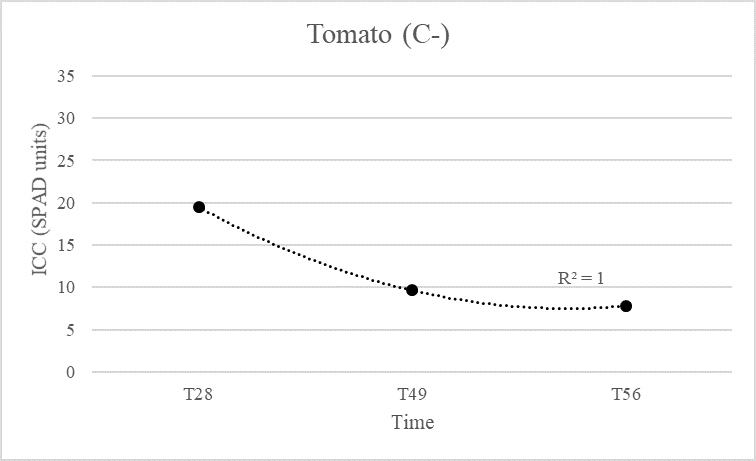 | 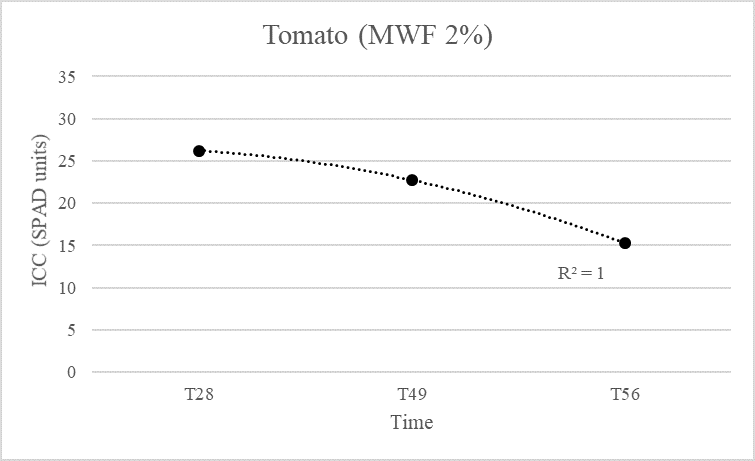 |
| --- | --- |
| 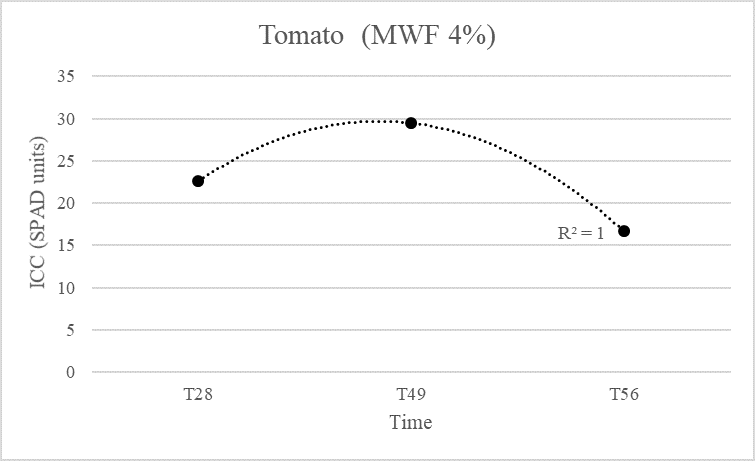 | 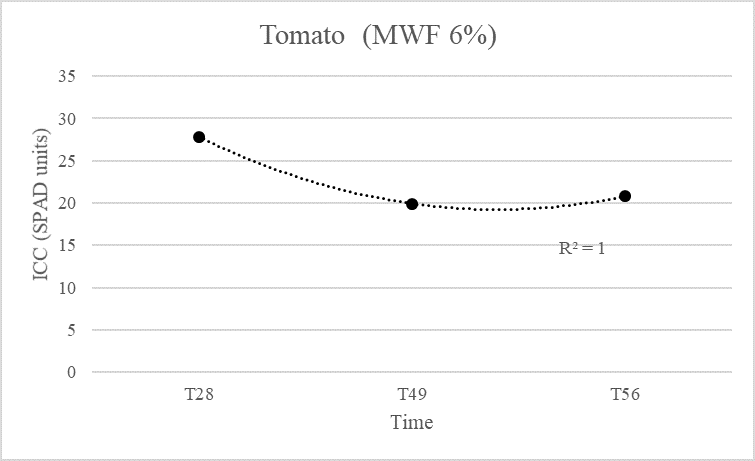 |
| 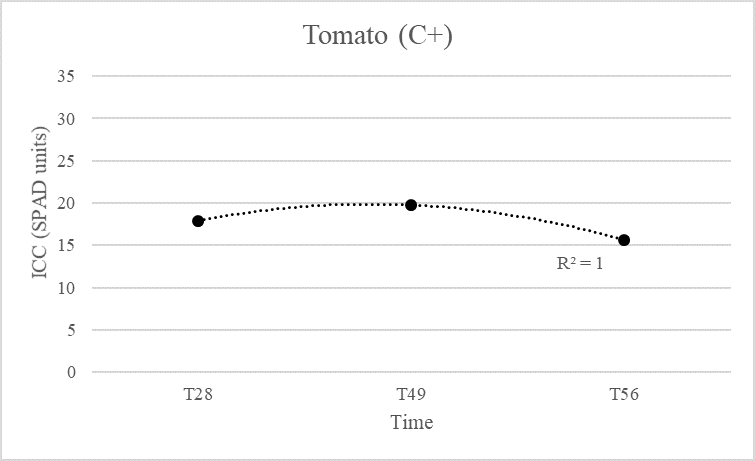 | |

**Figure S8. Dynamic changes in indirect chlorophyll content observed in sweet pepper treatments**. The graphs illustrate the mean variation in indirect chlorophyll content (ICC, measured in SPAD units) of sweet pepper (*Capsicum annuum* L. cv. ‘Altea’) across different treatments during the experimental period from Day 14 to Day 49 (DAT).

| 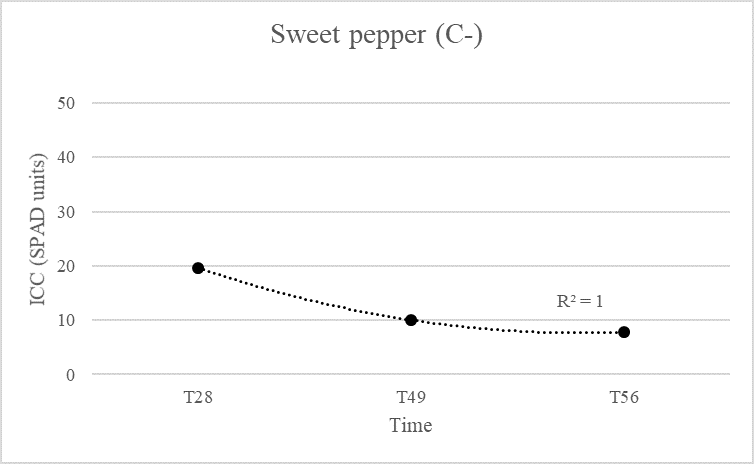 | 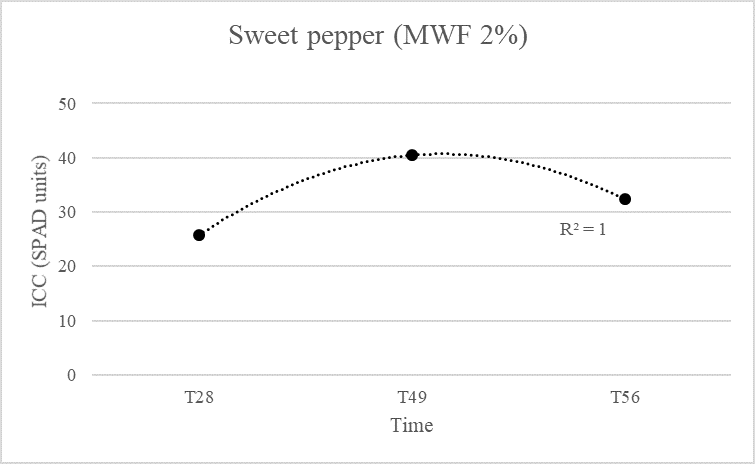 |
| --- | --- |
| 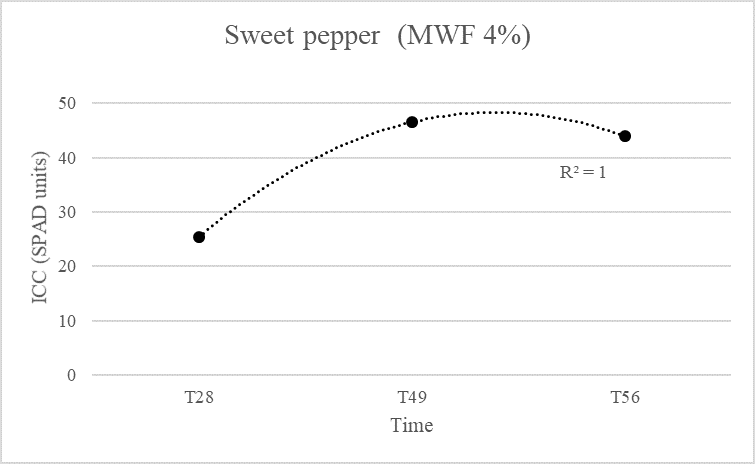 | 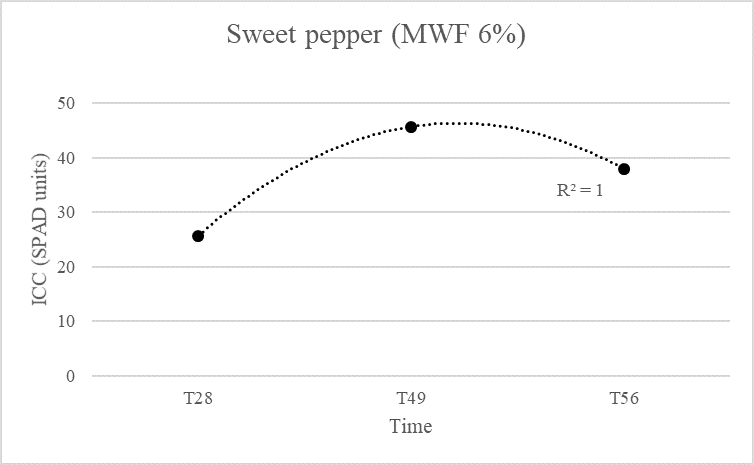 |
| 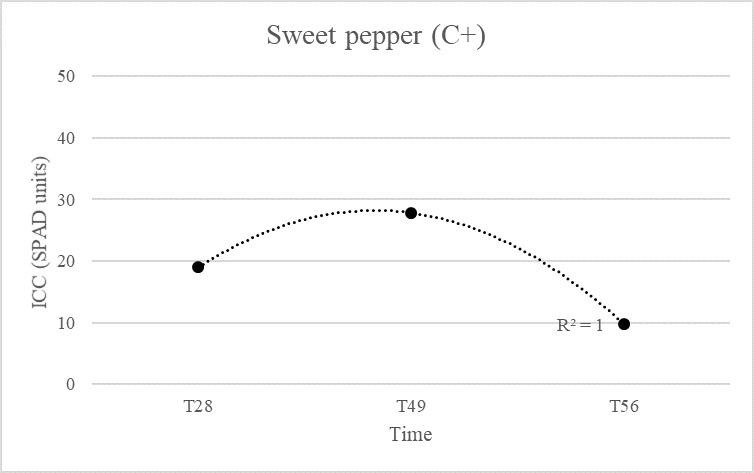 | |

**Figure S9. Dynamic changes in indirect chlorophyll content observed in eggplant treatments**. The graphs illustrate the mean variation in indirect chlorophyll content (ICC, measured in SPAD units) of eggplant (*Solanum melongena* L. cv. ‘Velia F1’) across different treatments during the experimental period from Day 14 to Day 49 (DAT).

| 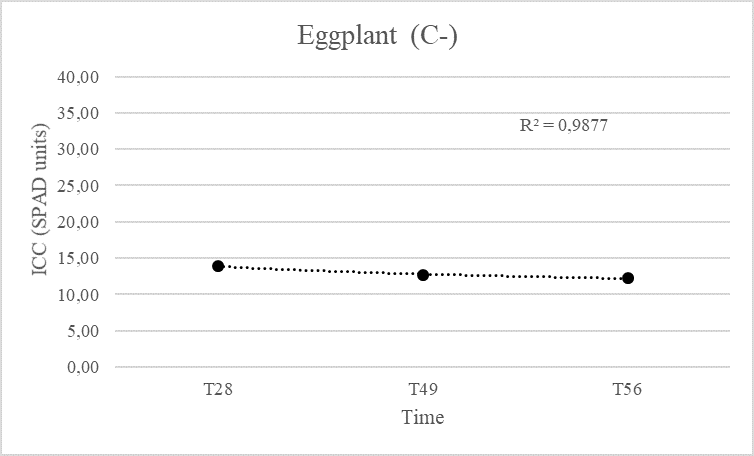 | 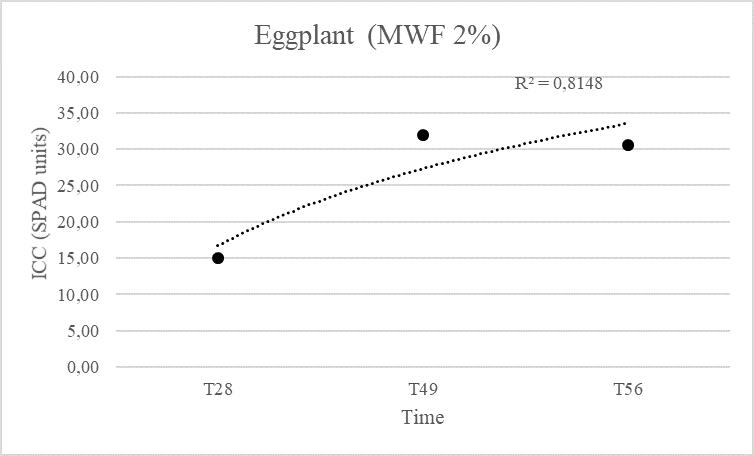 |
| --- | --- |
| 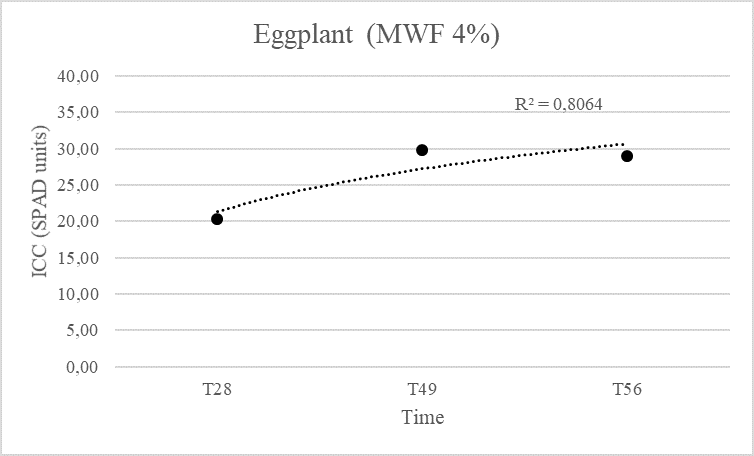 | 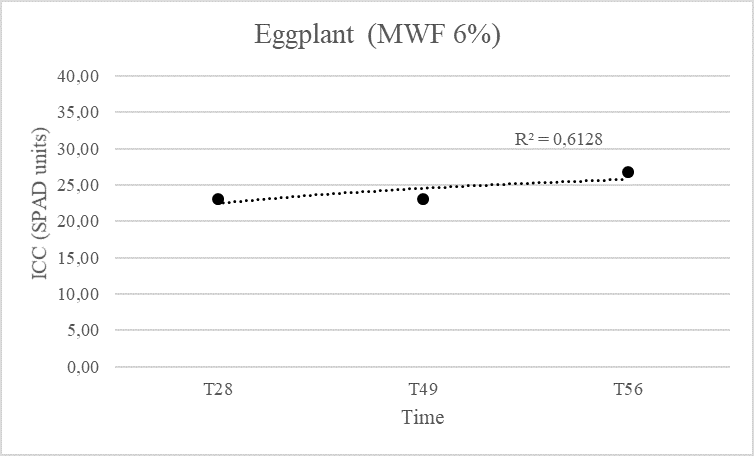 |
| 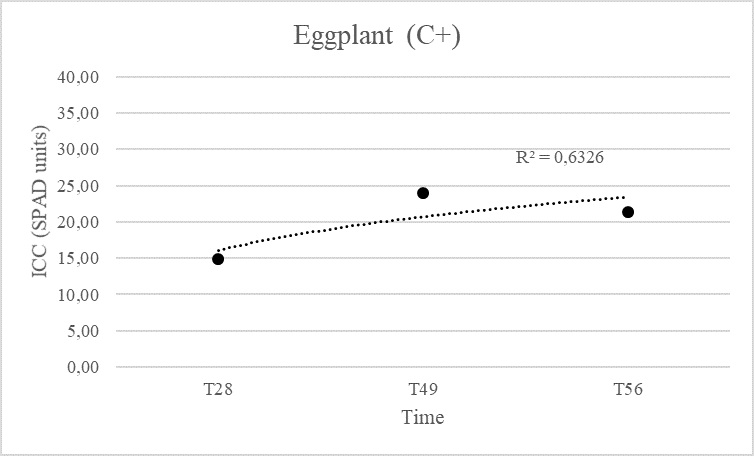 | |

**Figure S10. Progressive development of leaf area observed in tomato treatments**. The graphs illustrate the mean variation in leaf area (la) in cm^2^ of tomato (*Solanum lycopersicum* L. cv. ‘Creativo’) across different treatments during the experimental period from Day 14 to Day 49 (DAT).

| 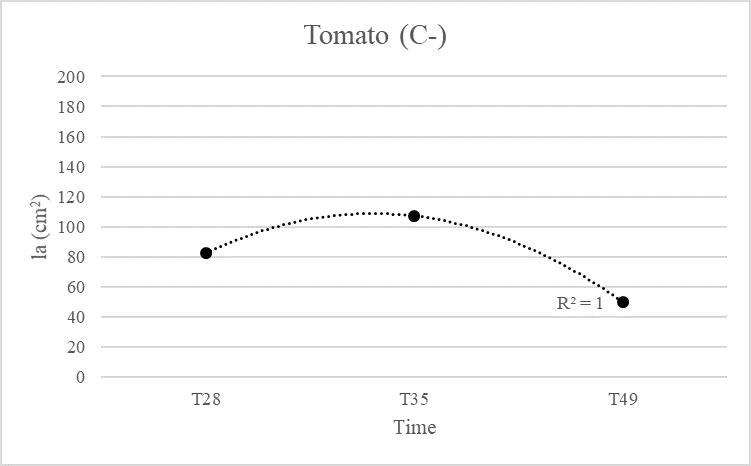 | 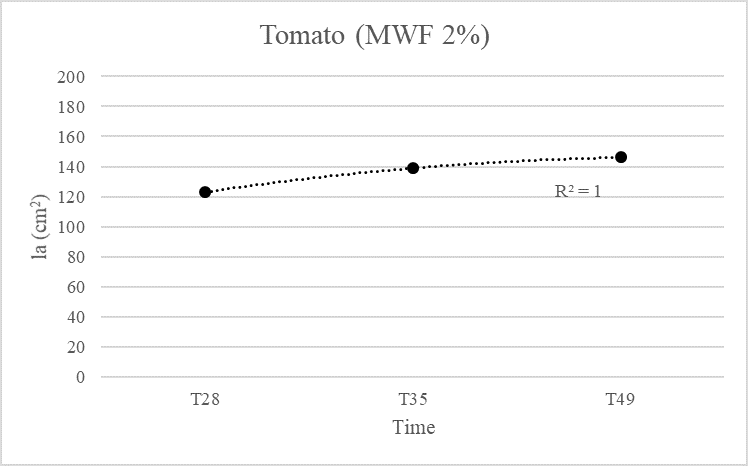 |
| --- | --- |
| 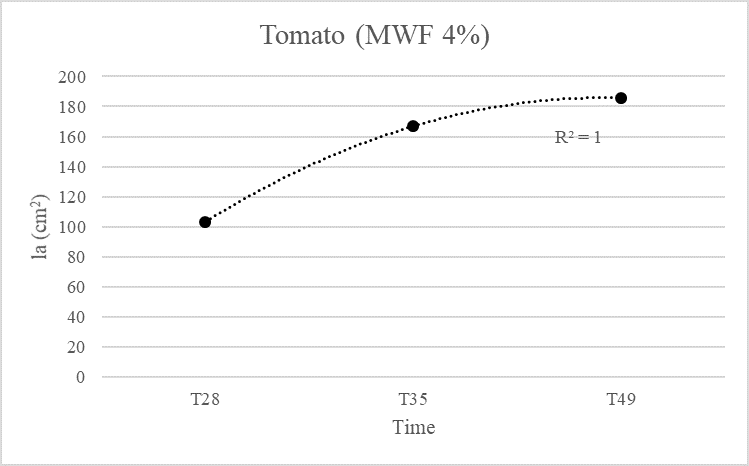 | 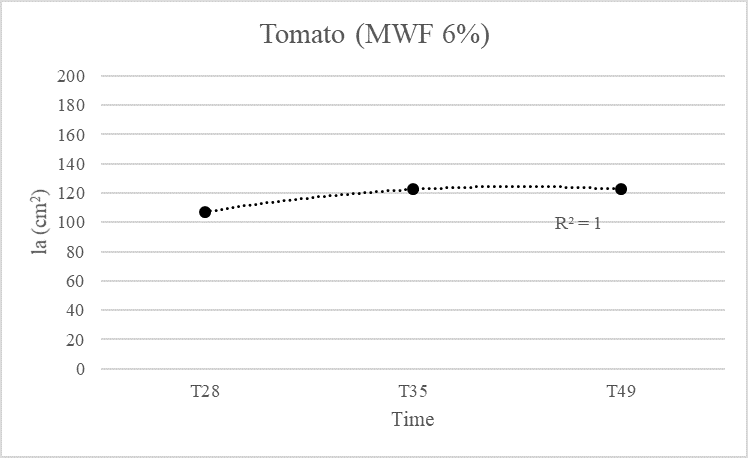 |
| 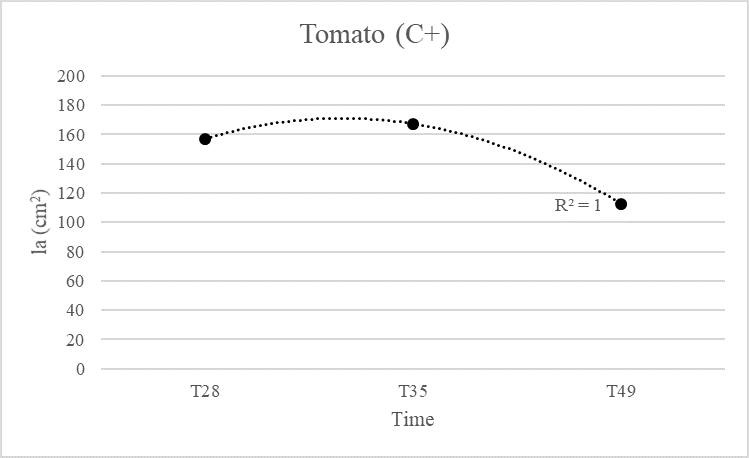 | |

**Figure S11. Progressive development of leaf area observed in sweet pepper treatments**. The graphs illustrate the mean variation in leaf area (la) in cm^2^ of sweet pepper (*Capsicum annuum* L. cv. ‘Altea’) across different treatments during the experimental period from Day 14 to Day 49 (DAT).

| 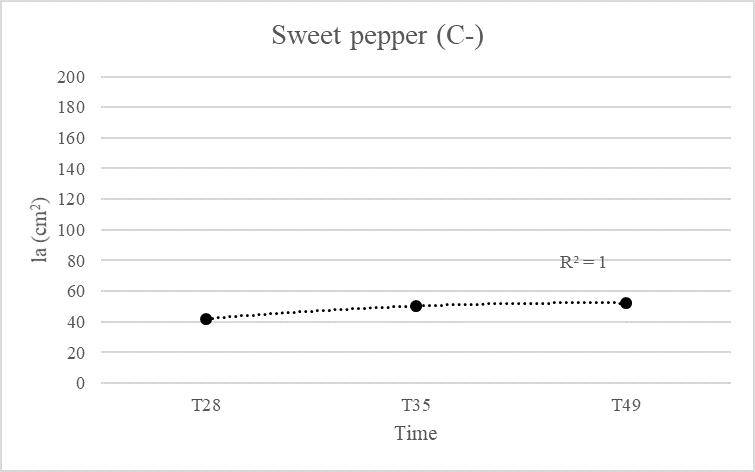 | 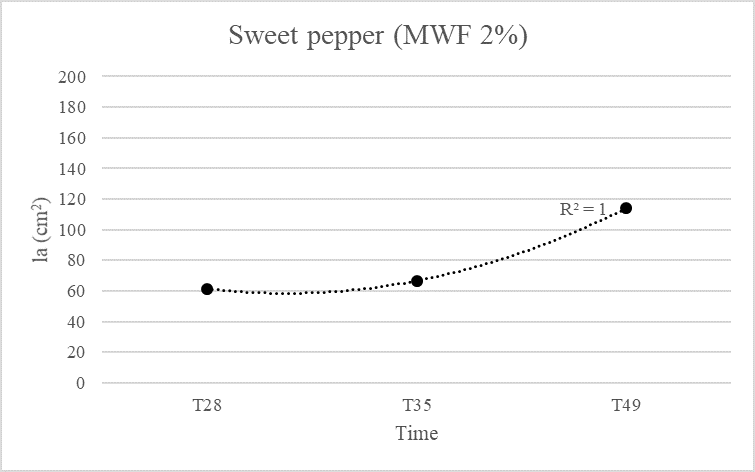 |
| --- | --- |
| 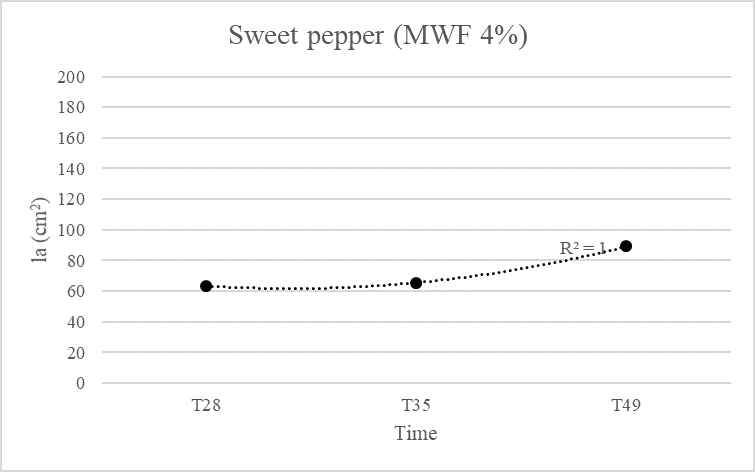 | 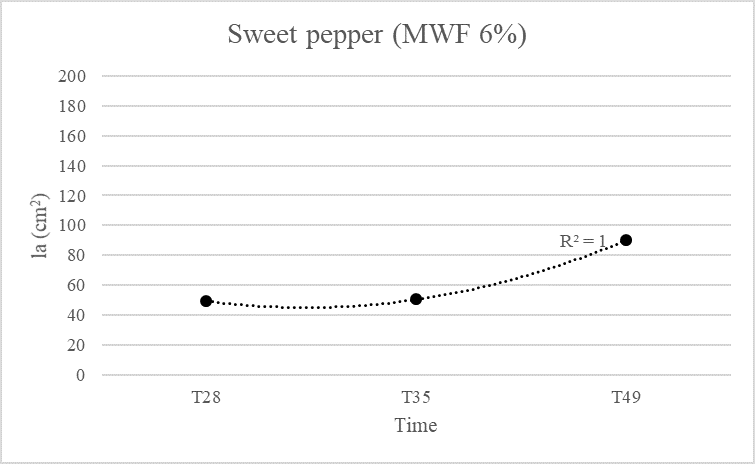 |
| 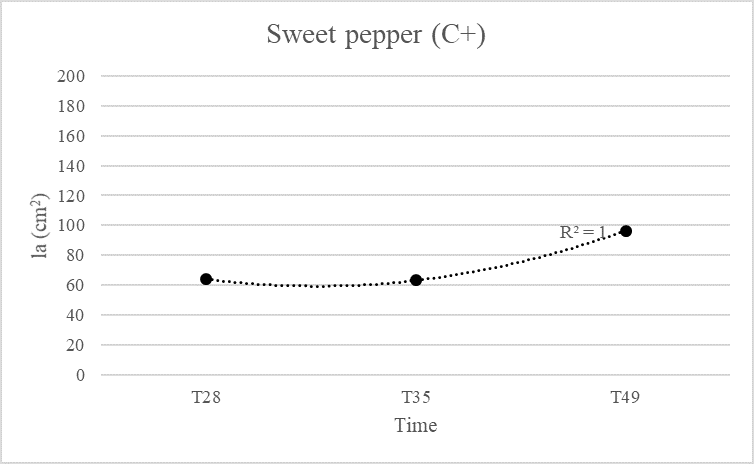 | |

**Figure S12. Progressive development of leaf area observed in eggplant treatments**. The graphs illustrate the mean variation in leaf area (la) in cm^2^ of eggplant (*Solanum melongena* L. cv. ‘Velia F1’) across different treatments during the experimental period from Day 14 to Day 49 (DAT).

| 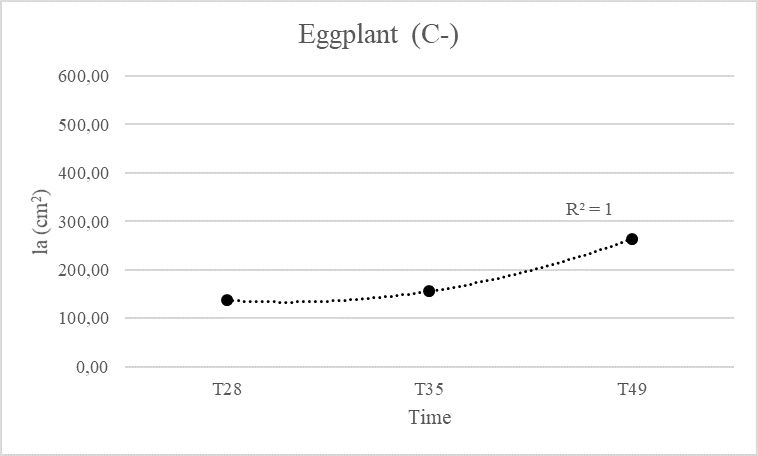 | 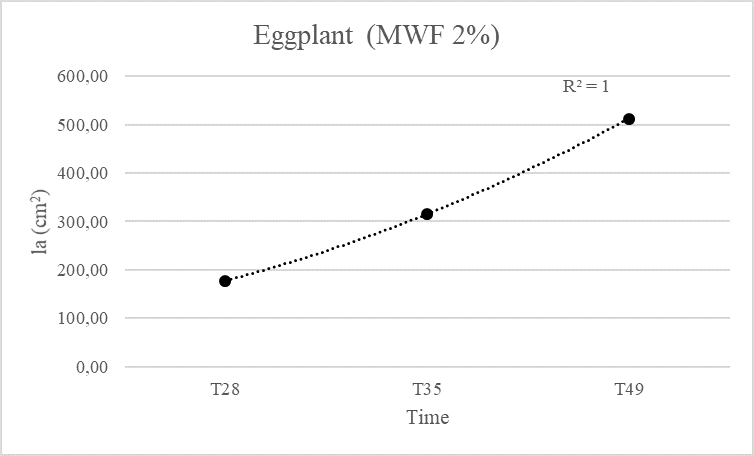 |
| --- | --- |
| 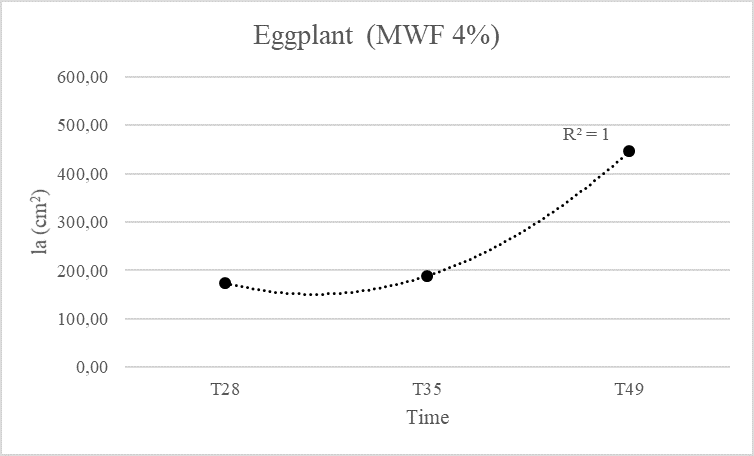 | 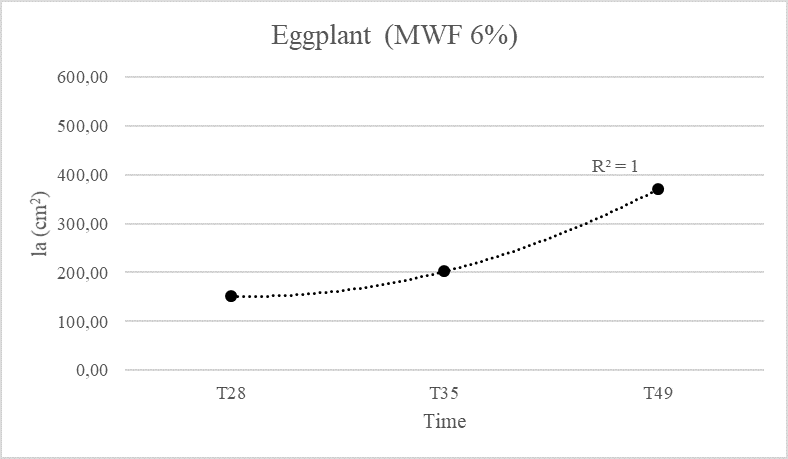 |
| 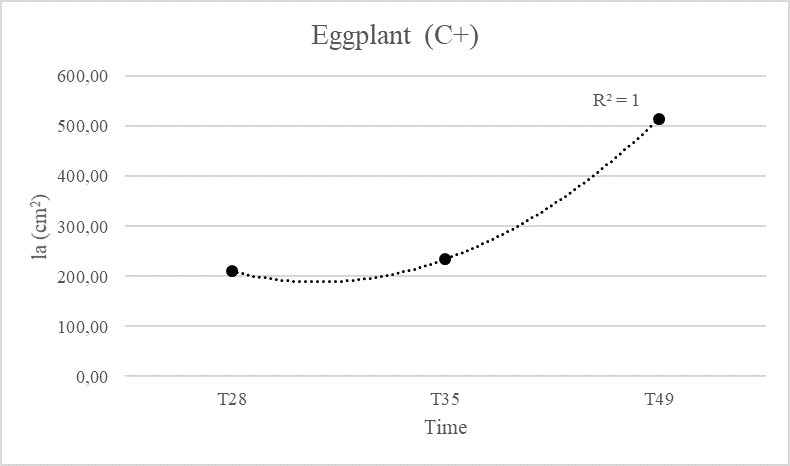 | |
